# Supplementary material for: Role of endoscopic ultrasonography in the diagnostic work-up of idiopathic acute pancreatitis (PICUS): study protocol for a nationwide prospective cohort study
Source: BMJ Open. 2020 Aug 20;10(8):e035504. doi: 10.1136/bmjopen-2019-035504 (PMC7440829; doi:10.1136/bmjopen-2019-035504)
Supplement: Supplementary data [file bmjopen-2019-035504supp001.pdf]

Additional file 1: Table S1 Drugs associated with acute pancreatitis

<table>
<tr><th colspan="5">Drugs associated with acute pancreatitis</th></tr>
<tr><td>Acetaminophen</td><td>Cisplatin</td><td>Hydrochlorothiazide</td><td>Methyldopa</td><td rowspan="2">Pentavalent antimony compounds</td></tr>
<tr><td>Asparaginase</td><td>Cytarabine</td><td>Interferon alpha</td><td>Metronidazole</td></tr>
<tr><td>Azathioprine</td><td>Didanosine</td><td>Itraconazole</td><td>Octreotide</td><td>Phenformin</td></tr>
<tr><td>Bortezomib</td><td>Enalapril</td><td>Lamivudine</td><td>Olanzapine</td><td>Simvastatin</td></tr>
<tr><td>Capecitabine</td><td>Erythromycin</td><td>Mercaptopurine</td><td>Opiates</td><td>Steroids</td></tr>
<tr><td>Carbamazepine</td><td>Estrogens</td><td>Mesalazine</td><td>Oxyphenbutazone</td><td>Sulfasalazine</td></tr>
<tr><td>Cimetidine</td><td>Furosemide</td><td>Olsalazine</td><td>Pentamidine</td><td>co-trimoxazole</td></tr>
</table>

Drugs with a definite association with acute pancreatitis (1, 2)

References

1. Nitsche C, Maertin S, Scheiber J, Ritter CA, Lerch MM, Mayerle J. Drug-induced pancreatitis. Curr Gastroenterol Rep. 2012;14(2):131-8.
2. Lankisch PG, Apte M, Banks PA. Acute pancreatitis. The Lancet. 2015;386(9988):85-96.
