# Supplementary material for: Role of endoscopic ultrasonography in the diagnostic work-up of idiopathic acute pancreatitis (PICUS): study protocol for a nationwide prospective cohort study
Source: BMJ Open. 2020 Aug 20;10(8):e035504. doi: 10.1136/bmjopen-2019-035504 (PMC7440829; doi:10.1136/bmjopen-2019-035504)
Supplement: Supplementary data [file bmjopen-2019-035504supp002.pdf]

Additional file 2: Relevant definitions

Acute pancreatitis: an acute inflammation of the pancreatic parenchyma, diagnosed when at least two of the three following characteristics are present (1):

1. Clinical features of acute pancreatitis, such as upper abdominal pain
2. Elevated serum amylase or lipase levels of at least three times the upper limit of normal (ULN)
3. Signs of acute pancreatitis on imaging

Note: no value of the required serum amylase or lipase level is provided as every participating center has a local laboratory, which is why each center may use different normal range values.

Idiopathic acute pancreatitis is considered to be present if no etiology is found in standard work-up, according to the IAP/APA evidence-based guidelines on management of acute pancreatitis (2), which comprises at least the following tests:

1. A detailed personal and family history, including questions on:
    a. Alcohol use
    b. Recent endoscopic retrograde cholangiopancreaticography (ERCP)
    c. Recent start of or changes in use of drugs associated with acute pancreatitis
    d. Recent major abdominal trauma
    e. Recent abdominal surgery
    f. Familial pancreatitis
    g. Hereditary pancreatitis

    h. Cystic fibrosis related pancreatitis

2. Laboratory tests, including:
    a. Blood serum triglycerides level on admission
    b. Blood serum calcium level, corrected for the serum albumin level, on admission
    c. Blood serum alanine transaminase (ALT) level on admission
3. Imaging via transabdominal ultrasound, magnetic resonance imaging (MRI) or magnetic resonance cholangiopancreaticography (MRCP) after clinical recovery

Note: side branch or mixed type intraductal papillary mucinous neoplasms (IPMN) without dilatation of the pancreatic duct will not be considered to be a causative factor for the pancreatitis episode.

Note: if the imaging is not able to discriminate between gall bladder polyps or concrements, lesions smaller than 10 mm will not be considered an exclusion criterion. Lesions above 10 mm, irrespective of whether they are a polyp or a concrement, are an immediate indication for cholecystectomy, and will be excluded from PICUS.

Alcoholic pancreatitis: pancreatitis caused by an excess intake of alcohol, diagnosed when biliary etiology is not demonstrated by standard work-up and the patient has indicated (either by direct or indirect personal history or by findings during physical examination) to have drank at least five units of alcohol in the 24 hours prior to start of abdominal complaints (or in asymptomatic acute pancreatitis: prior to diagnosis) (3-5)

Biliary pancreatitis: pancreatitis caused by biliary stones, microlithiasis or sludge, diagnosed when one of the following features is present:

1. A transient elevated ALT level of more than two times the ULN at diagnosis of acute pancreatitis, in the absence of ALT elevating comorbidity (6)
2. Signs of presence of gallstones, microlithiasis or sludge on imaging, defined as follows:
    a. Gallstones, microlithiasis and/or biliary sludge, either in the gall bladder, ductus cysticus, intrahepatic bile ducts or in the common bile duct (CBD), and/or
    b. A CBD of more than eight mm in patients 75 years old or younger or more than ten mm in patients older than 75 years at diagnosis of acute pancreatitis (7)

Note: no value of the required serum ALT level is provided as the normal range values depend on the sex of the patient and as every participating center has a local laboratory, which is why each center may use different normal range values.

Chronic pancreatitis**:** a chronic inflammation of the pancreatic parenchyma, defined as typical clinical history of chronic pancreatitis (such as recurrent pancreatitis or abdominal pain, except for primary painless pancreatitis) and one or more of the following (8):

1. Pancreatic calcifications
2. Moderate or marked ductal lesions, defined as two or more of the following abnormal features on transabdominal ultrasound, computed tomography (CT) or MRI/MRCP, according to the Cambridge classification (9):
    a. Main pancreatic duct abnormalities, either enlargement or increased echogenicity of the duct wall (mandatory)
    b. Pancreatic enlargement

    c. Cavities
    d. Duct irregularities including intraductal fillings defects, calculi or duct obstruction
    e. Focal acute pancreatitis
    f. Parenchymal heterogeneity
    g. Irregularities of pancreatic head or body contour
3. Moderate or marked ductal lesions, defined as five or more of the following abnormal features on endoscopic ultrasonography (EUS):
    a. Enlarged gland size
    b. Cysts
    c. Echo-poor lesions (focal areas of reduced echogenicity)
    d. Echo-rich lesions (more than three mm in diameter)
    e. Accentuation of lobular pattern (e.g., echo-poor normal parenchyma surrounded by hyperechoic strands)
    f. Increased duct wall echogenicity
    g. Irregularity of the main pancreatic duct (e.g., with narrowing of the duct)
    h. Dilation of the main pancreatic duct
    i. Visible side branches (e.g., with dilation)
    j. Calcification (of the pancreatic duct)
4. Marked and persistent exocrine insufficiency defined as pancreatic steatorrhea markedly reduced by enzyme supplementation
5. Typical histology of an adequate histological specimen

Note: during initial diagnostic work-up during admission ‘marked and persistent exocrine insufficiency’ cannot be evaluated properly. Therefore this part of the definition of chronic pancreatitis will not be applicable during standard work-up. However, if the patient does show marked and persistent exocrine insufficiency during follow-up (either during the outpatient clinic visit after repeat transabdominal ultrasound or after the EUS), this will be considered to be diagnostic for chronic pancreatitis. The same is applicable for histology of an adequate histological specimen: this is not part of standard work-up, however, if a typical histological specimen is obtained during follow-up, this will be considered to be diagnostic for chronic pancreatitis.

Clinical recovery from acute pancreatitis: resolution of pancreatic inflammation, present when one of the following criteria is met:

1. Discharge from the hospital
2. Normal inflammation parameters in laboratory tests
3. No signs of pancreatic inflammation on imaging

Cystic fibrosis: an autosomal recessive disorder caused by a mutation in the CFTR gene, resulting in defective chloride channels in epithelial cells, diagnosed by either a concentration in sweat of chloride greater than 60 mmol/L on repeated analysis, confirmation of a CFTR gene mutation, or both (10).

Cystic fibrosis related pancreatitis: pancreatitis caused by defective ductular and acinar pancreatic secretion, diagnosed when a patient with a history of cystic fibrosis presents with an acute pancreatitis in the absence of another origin (10).

Familial pancreatitis: acute pancreatitis from any cause that occurs in a family with an incidence that is greater than would be expected by chance alone, given the size of the family and the standardized incidence of pancreatitis within the Dutch population, defined as acute pancreatitis in patients who have two or more direct blood-related family members (parents, children or siblings) who have had an episode of acute pancreatitis (11-13).

Fever: a body temperature of 38.5˚C or higher.

Hereditary pancreatitis: otherwise unexplained pancreatitis in an individual from a family in which the pancreatitis phenotype appears to be inherited through a disease-causing gene mutation expressed in an autosomal dominant pattern, defined as pancreatitis in patients with a known mutation in the PRSS1 gene, the SPINK1 gene, the CFTR gene, the CTRC gene, the CLDN2 gene or the CPA1 gene, or if the patient has a direct family member (parents, children, siblings) with one or more of the above mentioned mutations and has at least one direct family member who has had an episode of acute pancreatitis or has chronic pancreatitis (13, 14).

Hypercalcemic pancreatitis: acute pancreatitis caused by hypercalcemia and diagnosed when no signs of a biliary pancreatitis are found in standard work-up and the patient has a blood serum calcium level of at least 12 mg/dl or 3 mmol/l, corrected for the serum albumin level, as first measured during admission (15).

Hypertriglyceridemic pancreatitis: acute pancreatitis based on hypertriglyceridemia and diagnosed if a biliary etiology is not demonstrated by standard work-up and the patient has a blood serum triglyceride level of at least 1000 mg/dl (or 11.2 mmol/l) under fasting conditions, as first measured during admission (16).

Hypothermia: a body temperature of 35.9˚C or lower.

Infected (extra)pancreatic necrosis: presence of microorganisms in (extra-)pancreatic necrosis, confirmed by a positive culture obtained by means of fine needle aspiration or from the first drainage procedure or necrosectomy, the presence of gas in the (extra-)pancreatic collection on CT, or the presence of clinical signs of persistent sepsis or progressive clinical deterioration despite maximal support on the intensive care unit (ICU) without other causes for infection (ruled out should be: pneumonia, urinary tract infection, wound infection, endocarditis, abdominal sepsis or any other infection which could be suspected based on the individual patient's clinical presentation) (17).

Medication associated pancreatitis: acute pancreatitis is considered to be caused by drugs when a biliary cause is not demonstrated by standard work-up, the patient uses one or multiple drug(s) listed in table S1 in additional file 1, the drug has been started or increased in dosage within a reasonable temporal sequence, in principle 1 month before the onset of the pancreatitis, and has a positive dechallenge (a drug reaction that is confirmed by stopping the drug) (18, 19).

Microlithiasis: stones or concrements, smaller than four mm, in the gall bladder or the bile ducts (20).

Murphy's sign: the phenomenon where compression of the right upper quadrant causes the patient to catch their breath due to pain when taking a deep breath (21).

Pancreas divisum: a congenital malformation of the main pancreatic duct (Wirsung's duct) with two separate ducts (a separate ventral duct of Wirsung and a dorsal duct of Santorini) as opposed to one main duct (of Wirsung) (22).

Positive imaging: positive imaging is defined as imaging during which a definitive cause for the acute pancreatitis episode can be found; or during which abnormalities are visualized constituting a definitive cause, after obtaining tissue and pathological examination. So, if during EUS ductal abnormalities are found, yet not enough to make a certain diagnosis of chronic pancreatitis according to the M-ANNHEIM classification (8), this imaging is considered to be negative, even though it did show abnormalities. This approach is chosen because the aim of this study is to determine the rate of which EUS can find a causative factor for a previous acute pancreatitis episode. For the same reason, finding of an anatomical abnormality after a first episode of acute pancreatitis is not scored as positive imaging. An overview of the exact findings scored as positive imaging is provided in table 3 of the main manuscript.

Post-ERCP pancreatitis: pancreatitis caused by mechanical injury from instrumentation and hydrostatic injury from contrast injection during ERCP, diagnosed if a patient develops a pancreatitis within 24 hours of an ERCP without indications of another origin (23).

Postoperative pancreatitis: pancreatitis caused by perioperative hypoperfusion of the pancreas, diagnosed if a patient develops a pancreatitis within 24 hours of abdominal surgery in the absence of indications for another origin (24).

Posttraumatic pancreatitis: pancreatitis caused by pancreatic injury due to trauma to the abdomen, diagnosed when the patient describes a typical blunt trauma to the upper abdomen and pancreatic trauma is visible on imaging (25).

Recurrence rate: the risk of a recurrent episode of acute pancreatitis.

Sludge: solid material which results from the slow settling of particles dispersed in bile (20).

Standard work-up:

1. A detailed personal and family history, including questions on:
    a. Alcohol use
    b. Recent ERCP
    c. Recent start of or changes in use of drugs associated with acute pancreatitis
    d. Recent major abdominal trauma
    e. Recent abdominal surgery
    f. Familial pancreatitis
    g. Hereditary pancreatitis
    h. Cystic fibrosis related pancreatitis
2. Laboratory tests, including:
    a. Blood serum triglycerides level, first measured during admission
    b. Blood serum calcium level, corrected for the serum albumin level, first measured during admission
    c. Blood serum ALT level on admission
3. Imaging via transabdominal ultrasound, MRI or MRCP after clinical recovery

Biliary events: acute cholecystitis; biliary colic's requiring readmission; biliary pancreatitis; cholangitis; or obstructive choledocholithiasis needing ERCP.

<u>Acute cholecystitis</u>: an acute inflammation of the gall bladder, diagnosed when one item in A, B and C is present:

A) Local signs of inflammation
   1. Murphy's' sign, or
   2. Right upper abdominal quadrant mass, pain or tenderness

B) Systemic signs of inflammation
   1. Fever or hypothermia, or
   2. Elevated C-reactive protein CRP), or
   3. Elevated white blood cell count

C) Imaging findings characteristic of acute cholecystitis (26, 27)

Note: acute cholecystitis and cholangitis (see definition below) are defined according to the Tokyo classification which defines fever as a body temperature of 38˚C or higher; however, fever will be defined in this study as hyperthermia of 38.5˚C or higher and hypothermia will be added as a systemic sign of inflammation, as this more accurately reflects clinical practice in the Netherlands.

<u>Biliary colic</u>**:** upper abdominal pain (either right upper quadrant or epigastric pain) lasting at least 30 minutes, often associated with restlessness (28).

BMJ Publishing Group Limited (BMJ) disclaims all liability and responsibility arising from any reliance placed on this supplemental material which has been supplied by the author(s)

Cholangitis: an inflammation of the bile duct(s), diagnosed when one item in each of the following categories is present:

1. Systemic inflammation
   a. Fever, hypothermia and/or shaking chills
   b. Laboratory data: evidence of inflammatory response (abnormal white blood cell counts (defined as smaller than 4,000/µl or larger than 10,000/µl), increase of serum CRP levels (defined as 1 mg/dl or higher), and other changes indicating inflammation)
2. Cholestasis
   a. Jaundice (defined as a total bilirubin of 2 mg/dl or higher)
   b. Laboratory data: abnormal liver function tests (increased serum alkaline phosphatase, gamma-glutamyltransferase (gamma-GT), aspartate transaminase (AST) and ALT levels (defined as more than 1.5 times the ULN))
3. Imaging
   a. Biliary dilatation
   b. Evidence of the etiology on imaging (stricture, stone, stent etc.) (26)

Obstructive choledocholithiasis: presence of gallstones, microlithiasis or biliary sludge in the CBD on imaging, requiring an ERCP, according to the treating physician.

References

1. Banks PA, Bollen TL, Dervenis C, Gooszen HG, Johnson CD, Sarr MG, et al. Classification of acute pancreatitis--2012: revision of the Atlanta classification and definitions by international consensus. Gut. 2013;62(1):102-11.
2. Working Group IAP/APA Acute Pancreatitis Guidelines. IAP/APA evidence-based guidelines for the management of acute pancreatitis. Pancreatology. 2013;13(4 Suppl 2):e1-15.
3. Stolle M, Sack PM, Thomasius R. Binge drinking in childhood and adolescence: epidemiology, consequences, and interventions. Dtsch Arztebl Int. 2009;106(19):323-8.
4. Sadr Azodi O, Orsini N, Andren-Sandberg A, Wolk A. Effect of type of alcoholic beverage in causing acute pancreatitis. Br J Surg. 2011;98(11):1609-16.
5. Midanik LT. Drunkenness, feeling the effects and 5+ measures. Addiction. 1999;94(6):887- 97.
6. Ammori BJ, Boreham B, Lewis P, Roberts SA. The Biochemical Detection of Biliary Etiology of Acute Pancreatitis on Admission: A Revisit in the Modern Era of Biliary Imaging. Pancreas. 2003;26:e32-5.
7. Schepers NJ, Bakker OJ, Besselink MG, Bollen TL, Dijkgraaf MG, van Eijck CH, et al. Early biliary decompression versus conservative treatment in acute biliary pancreatitis (APEC trial): study protocol for a randomized controlled trial. Trials. 2016;17:5.
8. Schneider A, Lohr JM, Singer MV. The M-ANNHEIM classification of chronic pancreatitis: introduction of a unifying classification system based on a review of previous classifications of the disease. J Gastroenterol. 2007;42(2):101-19.
9. Sarner M, Cotton PB. Classification of pancreatitis. Gut. 1984;25:756-9.
10. Ratjen F, Döring G. Cystic fibrosis. The Lancet. 2003;361(9358):681-9.
11. Spanier B, Bruno MJ, Dijkgraaf MG. Incidence and mortality of acute and chronic pancreatitis in the Netherlands: a nationwide record-linked cohort study for the years 1995-2005. World J Gastroenterol. 2013;19(20):3018-26.
12. CBS StatLine. Households; size, position in household, January 1st 1995-2013 StatLine: Central Bureau for Statistics 2015 [Available from: http://statline.cbs.nl/Statweb/publication/?VW=T&DM=SLNL&PA=37312&D1=a&D2=0,5,10,(l-2)-l&HD=180221-1109&HDR=G1&STB=T.

13. Whitcomb DC. Genetic aspects of pancreatitis. Annu Rev Med. 2010;61:413-24.

14. Whitcomb DC. Hereditary diseases of the pancreas. In: Yamada T AD, Kaplowitz N, Laine L, Owyang C, Powell DW, editors, editor. Textbook of Gastroenterology. 4th ed. Philadelphia: Lippincott Williams & Wilkins; 2003. p. 2147–65.

15. Shane E, Dinaz I. Hypercalcemia: Pathogenesis, clinical manifestations, differential diagnosis and management. In: Favus M, editor. Primer on the metabolic bone diseases and disorders of mineral metabolism. 6th ed. Philadelpiha: Kippincott, Williams, and Wilkins; 2006. p. 26-176.

16. Berglund L, Brunzell JD, Goldberg AC, Goldberg IJ, Sacks F, Murad MH, et al. Evaluation and treatment of hypertriglyceridemia: an Endocrine Society clinical practice guideline. J Clin Endocrinol Metab. 2012;97(9):2969-89.

17. van Brunschot S, van Grinsven J, van Santvoort HC, Bakker OJ, Besselink MG, Boermeester MA, et al. Endoscopic or surgical step-up approach for infected necrotising pancreatitis: a multicentre randomised trial. The Lancet. 2018;391(10115):51-8.

18. Nitsche C, Maertin S, Scheiber J, Ritter CA, Lerch MM, Mayerle J. Drug-induced pancreatitis. Curr Gastroenterol Rep. 2012;14(2):131-8.

19. Lankisch PG, Apte M, Banks PA. Acute pancreatitis. The Lancet. 2015;386(9988):85-96.

20. Jungst C, Kullak-Ublick GA, Jungst D. Gallstone disease: Microlithiasis and sludge. Best Pract Res Clin Gastroenterol. 2006;20(6):1053-62.

21. Murphy JB. The diagnosis of gall-stones. Am Med News. 1903;82:825.33.

22. Cotton P. Congenital anomaly of pancreas divisum as cause of obstructive pain and pancreatitis. Gut. 1980;21:105-14.

23. Cotton PB, Lehman G, Vennes J, Geenen JE, Russell RCG, Meyers WC, et al. Endoscopic sphincterotomy complications and their management: an attempt at consensus. Gastrointest Endosc. 1991;37:383-93.

24. Connor S. Defining post-operative pancreatitis as a new pancreatic specific complication following pancreatic resection. HPB (Oxford). 2016;18(8):642-51.

25. Booth F, Flint L. Pancreatoduodenal trauma. In: ed BJ, editor. Blunt multiple trauma. New York: Marcel Dekker; 1990. p. 497-509.

26. Miura F, Okamoto K, Takada T, Strasberg SM, Asbun HJ, Pitt HA, et al. Tokyo Guidelines 2018: initial management of acute biliary infection and flowchart for acute cholangitis. J Hepatobiliary Pancreat Sci. 2018;25(1):31-40.

27. Yokoe M, Takada T, Strasberg SM, Solomkin JS, Mayumi T, Gomi H, et al. TG13 diagnostic criteria and severity grading of acute cholecystitis (with videos). Journal of Hepato-Biliary-Pancreatic Sciences. 2013;20(1):35-46.

28. French E, Robb W. Biliary and renal colic. Br Med J. 1963:135-8.
