# Supplementary material for: Role of endoscopic ultrasonography in the diagnostic work-up of idiopathic acute pancreatitis (PICUS): study protocol for a nationwide prospective cohort study
Source: BMJ Open. 2020 Aug 20;10(8):e035504. doi: 10.1136/bmjopen-2019-035504 (PMC7440829; doi:10.1136/bmjopen-2019-035504)
Supplement: Supplementary data [file bmjopen-2019-035504supp003.pdf]

Additional file 3: Short Form 36 Questionnaire

English version

1. In general, would you say your health is:
   - ☐ excellent
   - ☐ very good
   - ☐ good
   - ☐ fair
   - ☐ poor

2. **Compared to one ear ago**, how would you rate your health in general **now**?
   - ☐ much better now than one year ago
   - ☐ somewhat better now than one year ago
   - ☐ about the same
   - ☐ somewhat worse now than one year ago
   - ☐ much worse now than one year ago

3. The following items are about activities you might do during a typical day. Does **your health now limit you** in these activities? If so, how much?

	**Yes, limited a lot**	**Yes, limited a little**	**No, not limited at all**
a. **Vigorous activities**, such as running, lifting heavy objects, participating in strenuous sports	☐	☐	☐
b. **Moderate activities**, such as moving a table, pushing a vacuum cleaner, bowling, or playing golf	☐	☐	☐
c. Lifting or carrying groceries	☐	☐	☐
d. Climbing **several** flights of stairs	☐	☐	☐
e. Climbing **one** flight of stairs	☐	☐	☐
f. Bending, kneeling, or stooping	☐	☐	☐
g. Walking **more than a mile**	☐	☐	☐
h. Walking **several blocks**	☐	☐	☐
i. Walking **one block**	☐	☐	☐
j. Bathing or dressing yourself	☐	☐	☐

4. During the **past 4 weeks**, have you had any of the following problems with your work or other regular daily activities **as a result of your physical health**?

	Yes	No
a. Cut down the **amount of time** you spent on work or other activities	☐	☐
b. **Accomplished less** than you would like	☐	☐
c. Were limited in the **kind** of work or other activities	☐	☐
d. Had **difficulty** performing the work or other activities (for example, it took extra effort)	☐	☐

5. During the **past 4 weeks**, have you had any of the following problems with your work or other regular daily activities **as a result of any emotional problems** (such as feeling depressed or anxious)?

	Yes	No
a. Cut down the **amount of time** you spent on work or other activities	☐	☐
b. **Accomplished less** than you would like	☐	☐
c. Didn’t do work or other activities as **carefully** as usual	☐	☐

6. During the **past 4 weeks**, to what extent has your physical health or emotional problems interfered with your normal social activities with family, friends, neighbors, or groups?

☐ not at all
☐ slightly
☐ moderately
☐ quite a bit
☐ extremely

7. How much **bodily** pain have you had during the **past 4 weeks**?

☐ none
☐ very mild
☐ mild
☐ moderate
☐ severe
☐ very severe

8. During the **past 4 weeks**, how much did **pain** interfere with your normal work (including both work outside the home and housework)?

☐ not at all
☐ a little bit
☐ moderately
☐ quite a bit
☐ extremely

9. These questions are about how you feel and how things have been with you **during the past 4 weeks**. For each question, please give the one answer that comes closest to the way you have been feeling.

How much of the time during the **past 4 weeks**…	**All of the time**	**Most of the time**	**A good bit of the time**	**Some of the time**	**A little of the time**	**None of the time**
a. Did you feel full of pep?	☐	☐	☐	☐	☐	☐
b. Have you been a very nervous person?	☐	☐	☐	☐	☐	☐
c. Have you felt so down in the dumps that nothing could cheer you up?	☐	☐	☐	☐	☐	☐
d. Have you felt calm and peaceful?	☐	☐	☐	☐	☐	☐
e. Did you have a lot of energy?	☐	☐	☐	☐	☐	☐
f. Have you felt downhearted and blue?	☐	☐	☐	☐	☐	☐
g. Did you feel worn out?	☐	☐	☐	☐	☐	☐
h. Have you been a happy person?	☐	☐	☐	☐	☐	☐
i. Did you feel tired?	☐	☐	☐	☐	☐	☐

10. During the **past 4 weeks**, how much of the time has **your physical health or emotional problems** interfered with your social activities (like visiting with friends, relatives, etc.)?

- ☐ all of the time
- ☐ most of the time
- ☐ some of the time
- ☐ a little of the time
- ☐ none of the time

11. How TRUE or FALSE is **each** of the following statements for you?

	**Definitely true**	**Mostly true**	**Don't know**	**Mostly false**	**Definitely false**
a. I seem to get sick a little easier than other people.	☐	☐	☐	☐	☐
b. I am as healthy as anybody I know.	☐	☐	☐	☐	☐
c. I expect my health to get worse.	☐	☐	☐	☐	☐
d. My health is excellent.	☐	☐	☐	☐	☐

Space for additional remarks with the questionnaire:

Dutch version

1. Wat vindt u, over het algemeen genomen, van uw gezondheid?	☐ uitstekend<br>☐ zeer goed<br>☐ goed<br>☐ matig<br>☐ slecht
2. In vergelijking *met 1 jaar geleden*, hoe zou u *nu* uw gezondheid in het algemeen beoordelen?	☐ veel beter dan een jaar geleden<br>☐ iets beter dan een jaar geleden<br>☐ ongeveer hetzelfde als een jaar geleden<br>☐ iets slechter dan een jaar geleden<br>☐ veel slechter dan een jaar geleden

3. De volgende vragen gaan over dagelijkse bezigheden. Wordt u door **uw gezondheid** *op dit moment* beperkt bij deze bezigheden? Zo ja, in welke mate?

	**Ja, ernstig**	**Ja, een beetje beperkt**	**Nee, helemaal niet beperkt**
a. *Forse inspanning* (zoals hardlopen, zware voorwerpen tillen, inspannend sporten)	☐	☐	☐
b. *Matige inspanning* (zoals het verplaatsen van een tafel, stofzuigen, fietsen)	☐	☐	☐
c. Tillen of boodschappen dragen	☐	☐	☐
d. *Een paar* trappen oplopen	☐	☐	☐
e. *Eén* trap oplopen	☐	☐	☐
f. Buigen, knielen of bukken	☐	☐	☐
g. *Meer dan een kilometer* lopen	☐	☐	☐
h. Een halve kilometer lopen	☐	☐	☐
i. *Honderd meter* lopen	☐	☐	☐
j. Uzelf wassen of aankleden	☐	☐	☐

4. Had u, ten gevolge van uw **lichamelijke gezondheid**, *de afgelopen 4 weken* één van de volgende problemen bij uw werk of andere bezigheden?

	Ja	Nee
a. U heeft *minder tijd* kunnen besteden aan werk of andere bezigheden.	☐	☐
b. U heeft *minder bereikt* dan u zou willen.	☐	☐
c. U was beperkt in het *soort* werk of soort bezigheden.	☐	☐
d. U had moeite met het werk of andere bezigheden (het kostte u bijvoorbeeld extra inspanning).	☐	☐

5. Had u, ten gevolge van een **emotioneel probleem** (bijvoorbeeld doordat u zich depressief of angstig voelde), *de afgelopen 4 weken* één van de volgende problemen bij uw werk of andere bezigheden?

	Ja	Nee
a. U heeft *minder tijd* kunnen besteden aan werk of andere bezigheden.	☐	☐
b. U heeft *minder bereikt* dan u zou willen.	☐	☐
c. U heeft het werk of andere bezigheden niet zo zorgvuldig gedaan als u gewend bent.	☐	☐

6. In hoeverre heeft uw **lichamelijke gezondheid** of hebben uw **emotionele problemen** u *de afgelopen 4 weken* belemmerd in uw normale sociale bezigheden met gezin, vrienden, buren of anderen?

☐ helemaal niet

☐ enigszins

☐ nogal

☐ veel

☐ heel erg veel

7. Hoeveel **pijn** had u *de afgelopen 4 weken?*

☐ geen

☐ heel licht

☐ licht

☐ nogal

☐ ernstig

☐ heel ernstig

8. In welke mate heeft **pijn** u *de afgelopen 4 weken* belemmerd bij uw normale werkzaamheden (zowel werk buitenshuis als huishoudelijk werk)?

☐ helemaal niet

☐ enigszins

☐ nogal

☐ veel

☐ heel erg veel

12. De volgende vragen gaan over hoe u zich *de afgelopen 4 weken* heeft **gevoeld**. Wilt u bij elke vraag het antwoord aankruisen dat het beste aansluit bij hoe u zich heeft gevoeld?

Hoe vaak gedurende *de afgelopen 4 weken:*	**Voortdurend**	**Meestal**	**Vaak**	**Soms**	**Zelden**	**Nooit**
a. voelde u zich levenslustig?	☐	☐	☐	☐	☐	☐
b. voelde u zich erg zenuwachtig?	☐	☐	☐	☐	☐	☐
c. zat u zo erg in de put dat niets u kon opvrolijken?	☐	☐	☐	☐	☐	☐
d. voelde u zich kalm en rustig?	☐	☐	☐	☐	☐	☐
e. voelde u zich erg energiek?	☐	☐	☐	☐	☐	☐
f. voelde u zich neerslachtig en somber?	☐	☐	☐	☐	☐	☐
g. voelde u zich uitgeblust?	☐	☐	☐	☐	☐	☐
h. voelde u zich gelukkig?	☐	☐	☐	☐	☐	☐
i. voelde u zich moe?	☐	☐	☐	☐	☐	☐

10. *Hoe vaak* hebben uw **lichamelijke gezondheid** of **emotionele problemen** gedurende *de afgelopen 4 weken* uw sociale activiteiten (zoals bezoek aan vrienden of naaste familieleden) belemmerd?

- ☐ voortdurend
- ☐ meestal
- ☐ soms
- ☐ zelden
- ☐ nooit

11. Wilt u het antwoord kiezen dat het beste weergeeft hoe juist of onjuist u elk van de volgende uitspraken voor uzelf vindt?

	**Volkomen juist**	**Grotendeels juist**	**Weet ik niet**	**Grotendeels onjuist**	**Volkomen onjuist**
a. Ik lijk gemakkelijker ziek te worden dan andere mensen.	☐	☐	☐	☐	☐
b. Ik ben net zo gezond als andere mensen die ik ken.	☐	☐	☐	☐	☐
c. Ik verwacht dat mijn gezondheid achteruit zal gaan.	☐	☐	☐	☐	☐
d. Mijn gezondheid is uitstekend.	☐	☐	☐	☐	☐

Ruimte voor aanvullende opmerkingen bij de vragenlijst:
