# Supplementary material for: Role of endoscopic ultrasonography in the diagnostic work-up of idiopathic acute pancreatitis (PICUS): study protocol for a nationwide prospective cohort study
Source: BMJ Open. 2020 Aug 20;10(8):e035504. doi: 10.1136/bmjopen-2019-035504 (PMC7440829; doi:10.1136/bmjopen-2019-035504)
Supplement: Author's manuscript [file bmjopen-2019-035504.draft_revisions.pdf]

BMJ Open is committed to open peer review. As part of this commitment we make the peer review history of every article we publish publicly available.

When an article is published we post the peer reviewers' comments and the authors' responses online. We also post the versions of the paper that were used during peer review. These are the versions that the peer review comments apply to.

The versions of the paper that follow are the versions that were submitted during the peer review process. They are not the versions of record or the final published versions. They should not be cited or distributed as the published version of this manuscript.

BMJ Open is an open access journal and the full, final, typeset and author-corrected version of record of the manuscript is available on our site with no access controls, subscription charges or pay-per-view fees (http://bmjopen.bmj.com).

If you have any questions on BMJ Open's open peer review process please email info.bmjopen@bmj.com

BMJ Open

The role of endoscopic ultrasonography in the diagnostic work-up of idiopathic acute pancreatitis (PICUS): study protocol for a nationwide prospective cohort study

Journal:	*BMJ Open*
Manuscript ID	bmjopen-2019-035504
Article Type:	Protocol
Date Submitted by the Author:	04-Nov-2019
Complete List of Authors:	Umans, Devica; Amsterdam University Medical Centres, Department of Gastroenterology and Hepatology; Saint Antonius Hospital, Research and Development<br>Timmerhuis, Hester; Saint Antonius Hospital, Department of Surgery; Saint Antonius Hospital, Research and Development<br>Hallensleben, Nora ; Erasmus Medical Center, Department of Gastroenterology and Hepatology; Saint Antonius Hospital, Research and Development<br>Bouwense, Stefan; Maastricht UMC+, Department of Surgery<br>Anten, Marie-Paule; Franciscus Gasthuis en Vlietland, Department of Gastroenterology and Hepatology<br>Bhalla, Abha; HagaZiekenhuis, Department of Gastroenterology and Hepatology<br>Bijlsma, Rina; Martini Ziekenhuis, Department of Gastroenterology and Hepatology<br>Boermeester, Marja; Amsterdam University Medical Centres, Department of Surgery<br>Brink, Menno; Meander MC, Department of Gastroenterology and Hepatology<br>Hol, Lieke; Maasstad Hospital, Department of Gastroenterology and Hepatology<br>Bruno, Marco; Erasmus Medical Center, Department of Gastroenterology and Hepatology<br>Curvers, Wouter; Catharina Hospital, Department of Gastroenterology and Hepatology<br>van Dullemen, Hendrik; UMCG, Department of Gastroenterology and Hepatology<br>van Eijck, Brechje; Spaarne Gasthuis, Department of Gastroenterology and Hepatology<br>Erkelens, G; Gelre Ziekenhuizen, Department of Gastroenterology and Hepatology<br>Fockens, Paul; Amsterdam University Medical Centres, Department of Gastroenterology and Hepatology<br>van Geenen, Erwin; Radboudumc<br>Hazen, Wouter; Elisabeth-TweeSteden Ziekenhuis, Department of Gastroenterology and Hepatology<br>Hoge, Chantal; Maastricht UMC+, Department of Gastroenterology and Hepatology<br>Inderson, Akin; LUMC, Department of Gastroenterology and Hepatology

	Kager, Liesbeth; Noordwest Ziekenhuisgroep, Department of Gastroenterology and Hepatology<br>Kuiken, Sjoerd; OLVG, Department of Gastroenterology and Hepatology<br>Perk, Lars; Medisch Centrum Haaglanden, Department of Gastroenterology and Hepatology<br>Poley, Jan-Werner<br>Quispel, Rutger; Reinier de Graaf Groep, Department of Gastroenterology and Hepatology<br>Römkens, Tessa; Jeroen Bosch Hospital, Department of Gastroenteroloy and Hepatology<br>van Santvoort, Hjalmar ; University Medical Center Utrecht; Saint Antonius Hospital, Department of Surgery<br>Tan, Adriaan; Canisius Wilhelmina Hospital, Department of Gastroenterology and Hepatology<br>Thijssen, Annemieke; Albert Schweitzer Ziekenhuis, Department of Gastroenterology and Hepatology<br>Venneman, Niels ; Medisch Spectrum Twente, Department of Gastroenterology and Hepatology<br>Vleggaar, Frank; Universitair Medisch Centrum Utrecht<br>Voorburg, Annet ; Diakonessenhuis Utrecht Zeist Doorn, Department of Gastroenterology and Hepatology<br>van Wanrooij, Roy; Amsterdam University Medical Centres, Department of Gastroenterology and Hepatology<br>Witteman, Ben; Ziekenhuis Gelderse Vallei, Department of Gastroenterology and Hepatology<br>Verdonk, Robert; Saint Antonius Hospital, Department of Gastroenterology and Hepatology<br>Besselink, Marc; Amsterdam University Medical Centres, Department of Surgery<br>van Hooft, Jeanin; AMC

Keywords:	Endoscopy < GASTROENTEROLOGY, Hepatobiliary disease < GASTROENTEROLOGY, Pancreatic disease < GASTROENTEROLOGY

SCHOLARONE™
Manuscripts

I, the Submitting Author has the right to grant and does grant on behalf of all authors of the Work (as defined in the below author licence), an exclusive licence and/or a non-exclusive licence for contributions from authors who are: i) UK Crown employees; ii) where BMJ has agreed a CC-BY licence shall apply, and/or iii) in accordance with the terms applicable for US Federal Government officers or employees acting as part of their official duties; on a worldwide, perpetual, irrevocable, royalty-free basis to BMJ Publishing Group Ltd ("BMJ") its licensees and where the relevant Journal is co-owned by BMJ to the co-owners of the Journal, to publish the Work in this journal and any other BMJ products and to exploit all rights, as set out in our licence.

The Submitting Author accepts and understands that any supply made under these terms is made by BMJ to the Submitting Author unless you are acting as an employee on behalf of your employer or a postgraduate student of an affiliated institution which is paying any applicable article publishing charge ("APC") for Open Access articles. Where the Submitting Author wishes to make the Work available on an Open Access basis (and intends to pay the relevant APC), the terms of reuse of such Open Access shall be governed by a Creative Commons licence – details of these licences and which Creative Commons licence will apply to this Work are set out in our licence referred to above.

Other than as permitted in any relevant BMJ Author's Self Archiving Policies, I confirm this Work has not been accepted for publication elsewhere, is not being considered for publication elsewhere and does not duplicate material already published. I confirm all authors consent to publication of this Work and authorise the granting of this licence.

For peer review only

The role of endoscopic ultrasonography in the diagnostic work-up of idiopathic acute pancreatitis (PICUS): study protocol for a nationwide prospective cohort study

Devica S Umans[1, 2], Hester C Timmerhuis[2, 3], Nora DL Hallensleben[2, 4], Stefan A Bouwense[5], Marie-Paule GF Anten[6], Abha Bhalla[7], Rina A Bijlsma[8], Marja A Boermeester[9], Menno A Brink[10], Lieke Hol[11], Marco J Bruno[4], Wouter L Curvers[12], Hendrik M van Dullemen[13], Brechje C van Eijck[14], G Willemien Erkelens[15], Paul Fockens[1], Erwin JM van Geenen[16], Wouter L Hazen[17], Chantal V Hoge[18], Akin Inderson[19], Liesbeth M Kager[20], Sjoerd D Kuiken[21], Lars E Perk[22], Jan-Werner Poley[4], Rutger Quispel[23], Tessa EH Römkens[24], Hjalmar C van Santvoort[3, 25], Adriaan CITL Tan[26], Annemieke Y Thijssen[27], Niels G Venneman[28], Frank P Vleggaar[29], Annet MCJ Voorburg[30], Roy LJ van Wanrooij[31], Ben J Witteman[32], Robert C Verdonk[33], Marc G Besselink[8], Jeanin E van Hooft[1], for the Dutch Pancreatitis Study Group

[1] Department of Gastroenterology and Hepatology, Amsterdam Gastroenterology and Metabolism, Amsterdam UMC, University of Amsterdam, Amsterdam, the Netherlands

[2] Department of Research and Development, St. Antonius Hospital, Nieuwegein, the Netherlands

[3] Department of Surgery, St. Antonius Hospital, Nieuwegein, the Netherlands

[4] Department of Gastroenterology and Hepatology, Erasmus Medical Center, Rotterdam, the Netherlands

[5] Department of Surgery, Maastricht UMC+, Maastricht, the Netherlands

[6] Department of Gastroenterology and Hepatology, Franciscus Gasthuis & Vlietland, Rotterdam, the Netherlands

[7] Department of Gastroenterology and Hepatology, Haga Hospital, Den Haag, the Netherlands

[8] Department of Gastroenterology and Hepatology, Martini Hospital, Groningen, the Netherlands

[9] Department of Surgery, Amsterdam UMC, University of Amsterdam, Amsterdam, the Netherlands

For peer review only

[10] Department of Gastroenterology and Hepatology, Meander Medical Center, Amersfoort, the Netherlands

[11] Department of Gastroenterology and Hepatology, Maasstad Hospital, Rotterdam, the Netherlands

[12] Department of Gastroenterology and Hepatology, Catharina Hospital, Eindhoven, the Netherlands

[13] Department of Gastroenterology and Hepatology, University Medical Center Groningen, Groningen, the Netherlands

[14] Department of Gastroenterology and Hepatology, Spaarne Gasthuis, Haarlem, the Netherlands

[15] Department of Gastroenterology and Hepatology, Gelre Hospital, Apeldoorn, the Netherlands

[16] Department of Gastroenterology and Hepatology, Radboud university medical center, Nijmegen, the Netherlands

[17] Department of Gastroenterology and Hepatology, Elisabeth-Tweesteden Hospital, Tilburg, the Netherlands

[18] Department of Gastroenterology and Hepatology, Maastricht University Medical Center, Maastricht, the Netherlands

[19] Department of Gastroenterology and Hepatology, Leiden University Medical Center, Leiden, the Netherlands

[20] Department of Gastroenterology and Hepatology, Noordwest Ziekenhuisgroep, Alkmaar, the Netherlands

[21] Department of Gastroenterology and Hepatology, OLVG, Amsterdam, the Netherlands

[22] Department of Gastroenterology and Hepatology, Medical Center Haaglanden, Den Haag, the Netherlands

[23] Department of Gastroenterology and Hepatology, Reinier de Graaf Gasthuis, Delft, the Netherlands

[24] Department of Gastroenterology and Hepatology, Jeroen Bosch Hospital, Den Bosch, the Netherlands

[25] Department of Surgery, UMC Utrecht, Utrecht, the Netherlands

[26] Department of Gastroenterology and Hepatology, Canisius-Wilhelmina Hospital, Nijmegen, the Netherlands

[27] Department of Gastroenterology and Hepatology, Albert Schweitzer Hospital, Dordrecht, the Netherlands

[28] Department of Gastroenterology and Hepatology, Medisch Spectrum Twente, Enschede, the Netherlands

[29] Department of Gastroenterology and Hepatology, University Medical Center Utrecht, the Netherlands

[30] Department of Gastroenterology and Hepatology, Diakonessenhuis, Utrecht, the Netherlands

[31] Department of Gastroenterology and Hepatology, Amsterdam Gastroenterology and Metabolism, Amsterdam UMC, Vrije Universiteit van Amsterdam, Amsterdam, the Netherlands

[32] Department of Gastroenterology and Hepatology, Hospital Gelderse Vallei, Ede, the Netherlands

For peer review only

For peer review only - http://bmjopen.bmj.com/site/about/guidelines.xhtml

[33] Department of Gastroenterology and Hepatology, St. Antonius Hospital, Nieuwegein, the Netherlands

Correspondence

Dr. Jeanin E. van Hooft, MD, PhD, MBA

Amsterdam UMC, location AMC

Department of Gastroenterology and Hepatology, C2-115

PO Box 22660

1100 DD Amsterdam

The Netherlands

Telephone: +31 20 566 7918

E-mail: j.e.vanhooft@amc.nl

Correspondence during review phase

Devica S Umans

Telephone: +31 88 320 7051

E-mail: d.s.umans@amsterdamumc.nl

For peer review only

List of abbreviations

ALT = alanine aminotransferase

BMI = body mass index

CBD = common bile duct

CI = confidence interval

CRF = case report form

CT = computed tomography

ERCP = endoscopic retrograde cholangiopancreaticography

EUS = endoscopic ultrasonography

IAP = idiopathic acute pancreatitis

IPMN = intraductal papillary mucinous neoplasm

IQR = interquartile range

MRCP = magnetic resonance cholangiopancreaticography

MRI = magnetic resonance imaging

Word count

Word count excluding title page, abstract, tables, acknowledgements, contributions and references: 3534

For peer review only

Abstract

Introduction

Idiopathic acute pancreatitis (IAP) remains a dilemma for physicians as it is uncertain whether patients with IAP may actually have an occult etiology. It is unclear to what extent additional diagnostic modalities such as endoscopic ultrasonography (EUS) are warranted after a first episode of IAP in order to uncover this etiology. Failure to timely determine treatable etiologies delays appropriate treatment and might subsequently cause recurrence of acute pancreatitis. Therefore, the aim of the PICUS study is to determine the value of routine EUS in determining the etiology of pancreatitis in patients with a first episode of IAP.

Methods and analysis

PICUS is designed as a multicenter prospective cohort study of 106 patients with a first episode of IAP after complete standard diagnostic work-up, in whom a diagnostic EUS will be performed. Standard diagnostic work-up will include a complete personal and family history, laboratory tests including serum alanine aminotransferase, calcium and triglyceride levels, and imaging by transabdominal ultrasound, magnetic resonance imaging or magnetic resonance cholangiopancreaticography after clinical recovery from the acute pancreatitis episode. The primary outcome measure is detection of etiology by EUS. Secondary outcome measures include pancreatitis recurrence rate, severity of recurrent pancreatitis, readmission, additional interventions, complications, length of hospital stay, quality of life, mortality and costs, during a follow-up period of 12 months.

For peer review only

For peer review only - http://bmjopen.bmj.com/site/about/guidelines.xhtml

Ethics and dissemination

PICUS is conducted according to the Declaration of Helsinki and Guideline for Good Clinical Practice. Five Medical Ethics Review Committees assessed PICUS. The results will be submitted for publication in an international peer-reviewed journal.

Conclusion

PICUS investigates the diagnostic yield of EUS in patients with a first episode of IAP and will determine whether routine EUS should be a part of the standard diagnostic work-up of a first episode of IAP.

Trial registration

Netherlands Trial Register: NL7066, June 9th 2018. Prospectively registered.

Article summary: strengths and limitations

- The PICUS study investigates the diagnostic yield of endoscopic ultrasonography in patients with a first episode of presumed idiopathic acute pancreatitis.
- This is the first prospective cohort studies of patients with a single episode of presumed IAP after complete standard diagnostic work-up (including exclusion based on blood serum ALT and imaging after clinical recovery).
- The results of the PICUS study will establish whether routine EUS should be incorporated in the guidelines for standard diagnostic work-up after a first episode of presumed IAP.

For peer review only

Keywords

Idiopathic acute pancreatitis; endoscopic ultrasonography, etiology

Background

Acute pancreatitis can be induced by numerous causes. Gallstone disease (approximately 50%) and alcohol (approximately 20%) are the most frequent causes (1-6), although the prevalence of etiologies of acute pancreatitis is dependent on, among other things, age and geographical factors (7-10). There is, however, a considerable group of patients of approximately 25% in whom no etiology can be found after routine diagnostic work-up (i.e. medical history, laboratory investigations and transabdominal ultrasound). These patients are considered to have presumed idiopathic acute pancreatitis (IAP) (3).

When IAP is presumed, guidelines recommend repeat transabdominal ultrasound after discharge (11, 12). This repeat ultrasonography has an additional diagnostic yield of 20% for the detection of gallstones or sludge in these patients (13). Undetected microlithiasis and biliary sludge are generally considered to be the major cause of presumed IAP (14, 15). Undetected and subsequently untreated gallstone disease poses a risk for recurrent acute pancreatitis and other biliary events, e.g. cholecystitis, biliary colic's and cholangitis.

Therefore, when previous diagnostics failed to uncover an etiology, endoscopic ultrasonography (EUS) should be considered for the detection of biliary disease or other abnormalities causing pancreatitis, such as neoplasms and chronic pancreatitis (11, 12, 16, 17). EUS is advised as the first step in presumed IAP, followed by (secretin-stimulated) magnetic resonance cholangiopancreaticography (MRCP) to identify rare morphologic abnormalities (11), as EUS is considered to have a higher diagnostic yield than MRCP for clinically relevant causes (18).

For peer review only - http://bmjopen.bmj.com/site/about/guidelines.xhtml

For peer review only

Although guidelines do recommend performing EUS after a first or second attack of presumed IAP, this recommendation is scored as a mere grade 2C, according to the GRADE classification (19) (indicating a weak recommendation based on evidence of low quality, with weak agreement among experts in this field) (11). Therefore, EUS is not routinely performed as the exact significance in this patient group is unclear (11, 16).

The PICUS study was designed to determine whether routine EUS should be incorporated in the standard diagnostic work-up of a first episode of presumed IAP.

Methods and analysis

Study aim

The objective of this study is to determine the diagnostic yield of EUS for the detection of etiology in patients with a first episode of presumed IAP.

Depending on the diagnostic yield of EUS observed in the PICUS study, incorporation of EUS in routine diagnostic work-up of patients with a first episode of presumed IAP will be considered. A minimal diagnostic yield of 10% for any etiology will be regarded as reasonable to justify implementing routine EUS in the standard diagnostic work-up of a first episode of presumed IAP.

Study design and setting

PICUS is a multicenter prospective cohort study. A total of 106 patients will be included from 28 participating Dutch centers, including all 8 university centers and 20 large teaching hospitals. A listing of the participating centers is included in the Authors' information. An overview of the study design, including screening procedures and follow-up, is provided in figure 1.

For peer review only - http://bmjopen.bmj.com/site/about/guidelines.xhtml

Study population

The subjects of this study have had a first episode of acute pancreatitis, as defined by the 2012 Revised Atlanta criteria (20), with an unknown origin after standard diagnostic work-up, according to the 2013 IAP/APA evidence-based guidelines on management of acute pancreatitis (11). The diagnostic modalities that constitute standard diagnostic work-up are listed in table 1. Potential etiologies and their definitions are listed in table 2.

Eligibility criteria

The inclusion criteria are:

1. Patients of 18 years or older
2. First episode of presumed IAP after standard diagnostic work-up
3. Informed consent for participation

The exclusion criteria are:

1. Known etiology
2. Chronic pancreatitis, as defined by the M-ANNHEIM criteria (21)
3. Recurrent pancreatitis
4. Altered anatomy which prohibits the endosonographist from visualizing the gall bladder, bile ducts, pancreas or pancreatic duct via EUS (e.g. gastric bypass surgery)
5. Diagnostic EUS aimed to determine etiology before inclusion

For peer review only - http://bmjopen.bmj.com/site/about/guidelines.xhtml

Endoscopic ultrasonography

EUS will be performed in routine clinical practice by an endosonographist. Use of linear or radial EUS will be at the discretion of the endosonographist. All Dutch endosonographists are trained to perform EUS according to the technique of Hawes and Fockens (22).

The endosonographist will systematically report, using a standardized Case Report Form (CRF), the experience of the endosonographist, visualization of anatomical structures (i.e. gall bladder, common bile duct (CBD) and pancreatic duct), presence of local complications of acute pancreatitis, characteristics of biliary etiology (i.e. gallstones, microlithiasis and/or biliary sludge), characteristics of chronic pancreatitis, presence of (a) pancreatic or peri-ampullary benign or malignant tumor(s), characteristics of auto-immune pancreatitis, anatomic variations (e.g. pancreas divisum) or other anomalies (e.g. cholecystitis, vascular, renal, splenic or hepatic anomalies or ascites), and performance of fine needle aspiration or fine needle biopsy. Additionally, the type of endoscope, use of sedation, procedure related complications and results of the fine needle aspiration or biopsy will be systematically recorded by the study coordinator in a separate CRF.

Primary outcome measure

The primary outcome measure is the number and ratio of patients with presumed IAP in whom EUS detects a cause for the pancreatitis episode.

A positive EUS is defined as an EUS during which a definitive cause for the acute pancreatitis episode has been found; or during which abnormalities are visualized constituting a definitive cause, after obtaining tissue and pathological examination. An overview of the exact findings scored as positive imaging is provided in table 3.

If during EUS pancreatic abnormalities are found, yet not enough to make a certain diagnosis of chronic pancreatitis according to the M-ANNHEIM classification (21), this imaging is considered to

For peer review only - http://bmjopen.bmj.com/site/about/guidelines.xhtml

be negative, even though it did show abnormalities. This approach is chosen because the aim of this study is to determine the rate of which EUS can find a cause for the presumed IAP episode. For the same reason, report of an anatomical abnormality during EUS after a first episode of acute pancreatitis is not scored as positive imaging as pancreatic morphological changes are very common in IAP and not necessarily clinically relevant, as is elaborated on in the discussion (23).

Secondary outcome measures

The secondary outcome measures are recurrence rate of acute pancreatitis, severity of recurrent pancreatitis (20), readmission, performance of additional invasive procedures (e.g. cholecystectomy, endoscopic sphincterotomy), complications of EUS and of additional interventions, according to the Clavien-Dindo classification (24), length of hospital stay, quality of life, mortality and costs. Relevant definitions are reported in Additional File 2.

Sample size calculation

The sample size calculation was based on the primary outcome measure, diagnostic yield of EUS. Based on two previous studies reporting yield in patients with a first episode of presumed IAP (25, 26), adjusted for the PICUS study criteria for inclusion (i.e. requiring negative imaging after clinical recovery) and for positive imaging (i.e. excluding pancreas divisum as etiology), diagnostic yield was assumed to be 30%. Using a two-sided significance level (α) of 0.05, a power ($1 - \beta$) of 80%, 95 patients are needed to attain a 95% confidence interval (CI) with a range smaller than 10% above and below the assumed yield of 30% (95% CI: 20.8, 39.2). Assuming a drop-out rate of 10%, a total of 106 patients will be included (27). The sample size was calculated using the software programs RStudio (28) and nQuery (29).

For peer review only - http://bmjopen.bmj.com/site/about/guidelines.xhtml

Follow-up

Data from patient records on primary and secondary outcome measures will be collected until 1 year after inclusion. Outpatient care and follow-up after the EUS is at the discretion of the treating physician, but an outpatient clinic visit after EUS to discuss the results of the EUS and potential subsequent appropriate treatment can be considered standard care.

In case of biliary disease, the patient will be considered for endoscopic retrograde cholangiopancreaticography (ERCP) with sphincterotomy when choledocho(-micro-)lithiasis or sludge in the CBD is present, and cholecystectomy, as is standard care for biliary pancreatitis. A (secretin-stimulated) MRCP will be recommended, if not performed earlier, if a patient is readmitted for a recurrent episode of acute pancreatitis after a negative EUS for etiology, in order to rule out structural anomalies such as pancreas divisum. This is in accordance with current guidelines (11).

Patients will be asked to fill out the Short Form-36 questionnaire in the validated Dutch translation on day 3 after inclusion, after 6 months and after 1 year.

Statistical aspects

All included subjects will be evaluated for primary and secondary endpoints until 1 year after inclusion. The primary analysis will be based on intention-to-treat principles. For exploratory reasons a per-protocol analysis will be performed too.

The intention-to-treat population comprises all patients included in the study, regardless of adherence to study protocol. The per-protocol population is the subset of included patients who were treated with the guidelines of the protocol. A tabular listing of all patients excluded from the intention-to-treat population will be provided together with the reasons for exclusion.

For peer review only

For peer review only - http://bmjopen.bmj.com/site/about/guidelines.xhtml

All analyses will be performed in SPSS for Microsoft Windows. All data handling and analysis will be saved in a syntax-file. Results will be presented with all centers combined. A two-tailed p-value of < 0.05 is considered statistically significant.

Baseline variables

The reported baseline characteristics consist of age, sex, body mass index (BMI), previous cholecystectomy, nicotine and alcohol use, severity of pancreatitis, length of hospital stay, amylase, lipase, C-reactive protein, alanine transaminase, calcium, albumin and triglycerides on admission, imaging modalities before EUS and their findings. Baseline characteristics of EUS will include timing of EUS, experience of endosonographist and type of sedation and type of endoscope used. Data will be presented in percentages or as mean with standard deviation, or in case of a skewed distribution as median with interquartile range (IQR).

Primary outcome measure: etiology detection rate

Overall detection rate of an etiology for the episode of acute pancreatitis will be presented as percentage with a 95% CI. Predefined subgroup analyses will be made for patients with and without obesity (cut-off at a BMI of 30), a previous cholecystectomy, alcohol use and local complications from the IAP episode. A subgroup analysis will also be made for patients with a transabdominal ultrasound as imaging after clinical recovery and with magnetic resonance imaging (MRI) or MRCP as imaging after clinical recovery. Finally, a subgroup analysis will be made for EUS performed by endosonographists with and without extensive experience (cut-off at 400 endosonographies performed), use of linear or radial scope and type of sedation used. In subgroup analyses, the Chi-square test or the Fisher's exact test will be used, as appropriate, to compare etiology detection rate between subgroups. In subgroup analyses, comparability between groups regarding baseline variables will be checked. If the subgroups

For peer review only - http://bmjopen.bmj.com/site/about/guidelines.xhtml

differ statistically significantly in one or more baseline variables, this will be corrected in a logistic regression analysis.

Secondary outcome measures

Secondary outcome measures will be described as percentages with 95% CI, as mean with standard deviation or median with IQR, as appropriate.

For recurrence rate, subgroup analyses will be made for patients with a positive and negative EUS, and in patients with a positive EUS, for patients who were and were not treated adequately. The same subgroup analyses as in the primary outcome measure, will also be applied on the recurrence rate. The Chi-square test or the Fisher's exact test will be used for comparison between subgroups, as appropriate.

For quality of life, subgroup analyses will be made for baseline versus follow-up quality of life and for patients with a positive and negative EUS, and with and without pancreatitis recurrence during follow-up. The (un-)paired T-test, Wilcoxon signed rank test or the Mann-Whitney U test will be used for comparisons between subgroups, as appropriate.

Cost analysis

The cost analysis will comprise direct medical costs, which are generated by healthcare utilization and include hospital admission periods and therapeutic and diagnostic procedures (30). Estimates of unit costs will be based on Dutch reference data from the cost guide of the Dutch Health Council (31). If this guide is an inappropriate determination of unit costs, the costs will be based on data provided by two hospital administrations (one university center and one general hospital) to account for the actual input of personnel, material and overhead over hospital resources used. Cost calculations will be used

For peer review only

to determine cost of interventions (surgical, endoscopic or radiological) and diagnostic imaging. The cost analysis will be reported separately from the main study manuscript.

Patient and public involvement

The patient advocacy organization *Alvleesklierverenigiging Nederland* was involved in the design of the PICUS study. The experience of the patient advocacy organization with IAP and participation in scientific research has driven the research question and design of the study with regards to patient burden. The patient advocacy organization will also be involved in the dissemination and implementation of the study results.

All patients eligible for participation will be asked to give written informed consent.

Ethics and dissemination

The PICUS study is conducted according to the principles of the Declaration of Helsinki (October 2013) and to the Guideline for Good Clinical Practice by the International Council for Harmonization (November 9 2016).

The need for ethical approval was waived by the Medical Ethics Review Committee of the Academic Medical Center on May 28, 2018 (W18_161 # 18.199), by the Medical Research Ethics Committee of the University Medical Center Utrecht on July 04, 2018 (18-469), by the Research Ethics Committee of Radboud university medical center on July 23, 2018 (2018-4520), by the Medical Ethics Review Committee of the Erasmus Medical Center on July 30, 2018 (MEC-2018-1293) and by the Medical Ethics Review Committee of the Maastricht University Medical Center on September 7, 2018 (2018-0685). Before start of inclusion, local board approval will be obtained in all participating centers.

For peer review only - http://bmjopen.bmj.com/site/about/guidelines.xhtml

The results of the PICUS study will be submitted for publication in an international peer-reviewed scientific journal, regardless of study outcomes.

Discussion

Previous research has suggested that EUS might be beneficial in the detection of an etiology in presumed IAP. However, data lacks on the efficacy of routine EUS in patients with a first episode of presumed IAP, after repeat imaging after clinical recovery is negative for an etiology. The PICUS study aims to determine whether routine EUS is warranted in a first episode of acute pancreatitis where no cause could be disclosed after complete standard diagnostic work-up.

Currently, guidelines do not clearly define criteria for biliary origin (11). However, it is generally agreed upon that cholelithiasis, microlithiasis or biliary sludge constitute biliary etiology. Several previous studies have shown an association between elevated ALT levels and acute biliary pancreatitis (32-35), with a positive predictive value of 85% for an ALT > 150 U/L within 48 hours after onset of symptoms (11, 32, 33, 35). Therefore, an elevated blood serum ALT level at admission is considered to entail a high probability of biliary etiology, and pancreatitis with an elevated ALT is treated as being of biliary origin (32-34, 36). However, the majority of current literature on EUS did not exclude patients based on ALT level at admission (15, 25, 26, 32, 37-46). As these patients have a higher a priori chance of confirmation of biliary etiology on EUS, the etiology detection rate of EUS might be overestimated in these studies. In PICUS, biliary etiology is defined as either the signs of cholelithiasis, microlithiasis or biliary sludge on transabdominal ultrasonography, or transient elevation of the blood serum ALT level of more than twice the upper limit of normal at admission. By only including patients with normal or slightly elevated ALT levels at admission, the etiology detection rate as reported in PICUS will reflect the detection rate in patients who are truly considered as having presumed IAP after standard diagnostic work-up.

For peer review only - http://bmjopen.bmj.com/site/about/guidelines.xhtml

Current guidelines advise a repeat transabdominal ultrasound after clinical recovery in the work-up of presumed IAP because the index transabdominal ultrasound is less sensitive during the acute phase of pancreatitis. The subpar visualization of gall bladder, bile ducts and pancreas is often due to excessive amounts of air in the intestines caused by pancreatitis-induced ileus and/or suboptimal cooperation of painful patients (47). After the first episode of acute pancreatitis, repeating a transabdominal ultrasound may be able to detect biliary stones where it could not during index admission (48). However, of the current literature on EUS in IAP, only a minority of studies included repeat imaging in the diagnostic work-up before EUS (15, 40, 41, 43). Previous research has shown that a repeat transabdominal ultrasound has a diagnostic yield of 20% in patients with a first episode of IAP (13). Omitting repeat imaging from diagnostic work-up before EUS may lead to an overestimation of the diagnostic yield of EUS. In PICUS, all patients are required to undergo imaging after clinical recovery, i.e. transabdominal ultrasound or MRI/MRCP. Computed tomography (CT) is not considered sufficient imaging as biliary disease, the most common underlying etiology in presumed IAP, cannot always be adequately detected using CT.

It is well documented that the overall diagnostic yield of EUS in patients with recurrent pancreatitis is superior to the diagnostic yield of both secretin-stimulated MRCP (s-MRCP) and non-secretin-stimulated MRCP (18, 44, 46, 49). In the subgroup of patients with a pancreas divisum, however, s-MRCP is considered to be superior in diagnostic yield to both EUS and MRCP (18). The role of pancreas divisum in the etiology of pancreatitis is unclear. Epidemiological studies have shown that the prevalence of pancreas divisum in the general population is equal to the prevalence in patients with presumed IAP (23). In patients with a pancreas divisum and acute pancreatitis, potentially other disease modifying factors add to the occurrence of pancreatitis, such as increased sensitivity to toxins or genetic susceptibility. Because of this ambiguity, pancreas divisum in patients with a first episode of acute pancreatitis is mostly left untreated in clinical practice. However, if patients with a pancreas divisum present with multiple episodes of presumed IAP, the divisum is often considered to be related to the pancreatitis and is subsequently treated, often with ERCP with endoscopic sphincterotomy,

For peer review only

For peer review only - http://bmjopen.bmj.com/site/about/guidelines.xhtml

although evidence supporting this practice is limited (23). Because of both the diagnostic superiority of EUS in recurrent pancreatitis as well as the lack of clinical consequences of (s-)MRCP in patients with a first episode of pancreatitis, EUS is preferred to (s-)MRCP as the first choice for additional diagnostic testing for etiology in patients with presumed IAP (18, 44, 46, 49). Subsequently, current guidelines advise performing MCRP in case of recurrent IAP after EUS fails to determine an etiology (11). Therefore, in PICUS, we have chosen not to systematically include (s-)MRCP in the diagnostic work-up before EUS of first episode IAP.

Current guidelines advise consideration of EUS after a first or second attack of IAP (11). However, there is a paucity of evidence on the efficacy of EUS in first episode IAP. Three previous studies prospectively reported on EUS in patients with first episode IAP (25, 26, 38). However, in these studies, patients were not excluded based on liver enzymes abnormalities suggestive of biliary disease and no repeat imaging after clinical recovery was performed. PICUS will be the first prospective cohort study in which EUS will be performed in patients with a first episode of IAP after complete standard diagnostic work-up before EUS according to current guidelines (11).

A diagnostic yield of 10% for any etiology will be considered reasonable to justify incorporating routine EUS after a first episode of presumed IAP. This cut-off value was determined during a multidisciplinary meeting of the Dutch Pancreatitis Study Group, which included the principal investigators of several trials being executed by the Dutch Pancreatitis Study Group. Considering the expectation that the majority of uncovered etiologies by EUS will be treatable (e.g. biliary disease) and adequate treatment could prevent pancreatitis recurrence, while in a minority of uncovered etiologies diagnosis before progression of disease might be crucial for prognosis (e.g. malignancy), a positive result in 10% of patients was deemed sufficient to warrant routine EUS after a first episode of presumed IAP.

In conclusion, the PICUS study is the first prospective cohort study of patients with a single episode of presumed IAP after complete standard diagnostic work-up (including exclusion based on

For peer review only - http://bmjopen.bmj.com/site/about/guidelines.xhtml

blood serum ALT and imaging after clinical recovery). The results of the PICUS study will establish whether routine EUS should be incorporated in the guidelines for standard diagnostic work-up after a first episode of presumed IAP.

Acknowledgements

The authors would like to acknowledge:

1. all of the members of the Dutch Pancreatitis Study Group, for their continuous efforts in recruitment of patients,
2. dr. S. van Dieren, for providing her expertise in the calculation of the sample size,
3. dr. M. van der Vlugt, MD, PhD, for her willingness to act as independent physician for the PICUS study, and
4. the pancreatic disease patient advocacy organization *Alvleesklierverenigingen Nederland*, for their effort in representing the perspective of (participating) patients during the study design.

For peer review only

References

1. Nesvaderani M, Eslick GD, Vagg D, Faraj S, Cox MR. Epidemiology, aetiology and outcomes of acute pancreatitis: A retrospective cohort study. Int J Surg. 2015;23(Pt A):68-74.
2. van Brunschot S, van Grinsven J, van Santvoort HC, Bakker OJ, Besselink MG, Boermeester MA, et al. Endoscopic or surgical step-up approach for infected necrotising pancreatitis: a multicentre randomised trial. The Lancet. 2018;391(10115):51-8.

For peer review only - http://bmjopen.bmj.com/site/about/guidelines.xhtml

3. Bakker OJ, van Brunschot S, van Santvoort HC, Besselink MG, Bollen TL, Boermeester MA, et al. Early versus on-demand nasoenteric tube feeding in acute pancreatitis. N Engl J Med. 2014;371(21):1983-93.

4. Besselink MGH, van Santvoort HC, Buskens E, Boermeester MA, van Goor H, Timmerman HM, et al. Probiotic prophylaxis in predicted severe acute pancreatitis: a randomised, double-blind, placebo-controlled trial. The Lancet. 2008;371(9613):651-9.

5. Olaf J. Bakker M, Hjalmar C. van Santvoort M, PhD, Sandra van Brunschot M, Ronald B. Geskus P, Marc G. Besselink M, PhD, Thomas L. Bollen M, et al. Endoscopic Transgastric vs Surgical Necrosectomy for Infected Necrotizing Pancreatitis A Randomized Trial. JAMA. 2012;307(10):1053-61.

6. Hjalmar C. van Santvoort MD, Marc G. Besselink, M.D., Ph.D., Olaf J. Bakker, M.D., H. Sijbrand Hofker, M.D., Marja A. Boermeester, M.D., Ph.D., Cornelis H. Dejong, M.D., Ph.D., Harry van Goor, M.D., Ph.D., Alexander F. Schaapherder, M.D., Ph.D., Casper H. van Eijck, M.D., Ph.D., Thomas L. Bollen, M.D., Bert van Ramshorst, M.D., Ph.D., Vincent B. Nieuwenhuijs, M.D., Ph.D., Robin Timmer, M.D., Ph.D., Johan S. Laméris, M.D., Ph.D., Philip M. Kruyt, M.D., Eric R. Manusama, M.D., Ph.D., Erwin van der Harst, M.D., Ph.D., George P. van der Schelling, M.D., Ph.D., Tom Karsten, M.D., Ph.D., Eric J. Hesselink, M.D., Ph.D., Cornelis J. van Laarhoven, M.D., Ph.D., Camiel Rosman, M.D., Ph.D., Koop Bosscha, M.D., Ph.D., Ralph J. de Wit, M.D., Ph.D., Alexander P. Houdijk, M.D., Ph.D., Maarten S. van Leeuwen, M.D., Ph.D., Erik Buskens, M.D., Ph.D., and Hein G. Gooszen, M.D., Ph.D. A Step-up Approach or Open Necrosectomy for Necrotizing Pancreatitis. N Engl J Med. 2010;362(16):1491-502.

7. Sadr-Azodi O, Oskarsson V, Discacciati A, Videhult P, Askling J, Ekbom A. Pancreatic Cancer Following Acute Pancreatitis: A Population-based Matched Cohort Study. Am J Gastroenterol. 2018;113(11):1711-9.

8. Yamin Zheng M, Zhen Zhou, PhD, Haichao Li, BS Med, Jia Li, MD, Ang Li,MD, Binrong Ma, PhD, Taiping Zhang, MD, Quan Liao, MD, Yingjiang Ye, MD, Zhongtao Zhang, MD, Yinmo Yang, MD, Zhenjun Wang, MD, Zaixing Zhang, MD, Jinshan Yang, BS Med, and Fei Li, MD. A Multicenter Study on Etiology of Acute Pancreatitis in Beijing During 5 Years. Pancreas. 2015;44:409-14.

For peer review only

For peer review only - http://bmjopen.bmj.com/site/about/guidelines.xhtml

463 9. Zhu Y, Pan X, Zeng H, He W, Xia L, Liu P, et al. A Study on the Etiology, Severity, and Mortality of 3260 Patients With Acute Pancreatitis According to the Revised Atlanta Classification in Jiangxi, China Over an 8-Year Period. Pancreas. 2017;46(4):504-9.

466 10. Roberts SE, Morrison-Rees S, John A, Williams JG, Brown TH, Samuel DG. The incidence and aetiology of acute pancreatitis across Europe. Pancreatology. 2017;17(2):155-65.

468 11. Working Group IAP/APA Acute Pancreatitis Guidelines. IAP/APA evidence-based guidelines for the management of acute pancreatitis. Pancreatology. 2013;13(4 Suppl 2):e1-15.

470 12. Working Party of the British Society of G, Association of Surgeons of Great B, Ireland, Pancreatic Society of Great B, Ireland, Association of Upper GISoGB, et al. UK guidelines for the management of acute pancreatitis. Gut. 2005;54 Suppl 3:iii1-9.

473 13. Hallensleben ND, Umans DS, Bouwense S, Verdonk RC, Besselink MG, Van Hooft JE, et al. The clinical course and diagnostic work-up of idiopathic acute pancreatitis, a post-hoc analysis of a prospective multicenter observational cohort. Digestive Disease Week; San Diego: Gastroenterology; 2019. p. S-121.

477 14. Lee SP, Nicholls JF, Park HZ. Biliary Sludge as a Cause of Acute Pancreatitis. N Engl J Med. 1992;326:589-93.

479 15. Garg PK, Tandon RK, Madan K. Is biliary microlithiasis a significant cause of idiopathic recurrent acute pancreatitis? A long-term follow-up study. Clin Gastroenterol Hepatol. 2007;5(1):75-9.

481 16. Tenner S, Baillie J, DeWitt J, Vege SS, American College of G. American College of Gastroenterology guideline: management of acute pancreatitis. Am J Gastroenterol. 2013;108(9):1400-15; 16.

484 17. Banks PA, Freeman ML, Practice Parameters Committee of the American College of G. Practice guidelines in acute pancreatitis. Am J Gastroenterol. 2006;101(10):2379-400.

486 18. Wan J, Ouyang Y, Yu C, Yang X, Xia L, Lu N. Comparison of EUS with MRCP in idiopathic acute pancreatitis: a systematic review and meta-analysis. Gastrointest Endosc. 2018.

For peer review only - http://bmjopen.bmj.com/site/about/guidelines.xhtml

19. Schünemann H BJ, Guyatt G, Oxman A, editors. GRADE handbook for grading quality of evidence and strength of recommendations. Group TGW, editor2013.

20. Banks PA, Bollen TL, Dervenis C, Gooszen HG, Johnson CD, Sarr MG, et al. Classification of acute pancreatitis--2012: revision of the Atlanta classification and definitions by international consensus. Gut. 2013;62(1):102-11.

21. Schneider A, Lohr JM, Singer MV. The M-ANNHEIM classification of chronic pancreatitis: introduction of a unifying classification system based on a review of previous classifications of the disease. J Gastroenterol. 2007;42(2):101-19.

22. Hawes RH, Fockens P. Endosonography. Second edition ed. Philadelphia: Elsevier Inc.; 2011.

23. DiMagno MJ, Wamsteker EJ. Pancreas divisum. Curr Gastroenterol Rep. 2011;13(2):150-6.

24. Dindo D, Demartines N, Clavien P-A. Classification of Surgical Complications. Annals of Surgery. 2004;240(2):205-13.

25. Yusoff IF, Raymond G, Sahai AV. A prospective comparison of the yield of EUS in primary vs. recurrent idiopathic acute pancreatitis. Gastrointest Endosc. 2004;60(5):673-8.

26. Vila JJ, Vicuna M, Irisarri R, de la Higuera BG, Ruiz-Clavijo D, Rodriguez-Gutierrez C, et al. Diagnostic yield and reliability of endoscopic ultrasonography in patients with idiopathic acute pancreatitis. Scand J Gastroenterol. 2010;45(3):375-81.

27. Elise Whitley JB. Statistics review 4: Sample size calculations. Critical Care 2002;6(4):335-41.

28. R-Core-Team. R: A Language and Environment for Statistical Computing. Vienna, Austria: The R Foundation for Statistical Computing; 2017.

29. Michael Ray Oliver P, Kaaren Yeghiazarian, Min Zheng, Mora Jamshidian, Igor Koyfman. nQuery Advisor. 1995-2007.

30. Gold MR, Siegel JE, Russel LB. Cost-effectiveness in Health and Medicine. New York: Oxford University Press; 1996.

For peer review only

For peer review only - http://bmjopen.bmj.com/site/about/guidelines.xhtml

31. Netherlands) ZNHI. Kostenhandleiding: Methodologie van kostenonderzoek en referentieprijzen voor economische evaluaties in de gezondheidszorg (Cost Guide: Methology of cost analysis and reference prices for economical evaluations in health care) 2015.

32. Liu CL, Fan ST, Lo CM, Tso WK, Wong Y, Poon RT, et al. Clinico-biochemical prediction of biliary cause of acute pancreatitis in the era of endoscopic ultrasonography. Aliment Pharmacol Ther. 2005;22(5):423-31.

33. Moolla Z, Anderson F, Thomson SR. Use of amylase and alanine transaminase to predict acute gallstone pancreatitis in a population with high HIV prevalence. World J Surg. 2013;37(1):156-61.

34. B. J. Ammori BB, P. Lewis, and S. A. Roberts. The Biochemical Detection of Biliary Etiology of Acute Pancreatitis on Admission: A Revisit in the Modern Era of Biliary Imaging. Pancreas. 2003;26(2):e32–e5.

35. Tenner SD, H.; Steinberg, W. Predicting gallstone pancreatitis with laboratory parameters: a meta-analysis. Am J Gastroenterol. 1994;89:1863-6.

36. Schepers NJ, Bakker OJ, Besselink MG, Bollen TL, Dijkgraaf MG, van Eijck CH, et al. Early biliary decompression versus conservative treatment in acute biliary pancreatitis (APEC trial): study protocol for a randomized controlled trial. Trials. 2016;17:5.

37. Frossard JL, Sosa-Valencia L, Amouyal G, Marty O, Hadengue A, Amouyal P. Usefulness of Endoscopic Ultrasonography in Patients with “Idiopathic” Acute Pancreatitis. Am J Med. 2000;109:196-200.

38. Govil A, Agrawal MK, Agrawal D, Udawat H. Role of endoscopic ultrasonography in patients with first episode of idiopathic acute pancreatitis. Indian J Gastroenterol. 2014;33(3):241-8.

39. Mariani A, Arcidiacono PG, Curioni S, Giussani A, Testoni PA. Diagnostic yield of ERCP and secretin-enhanced MRCP and EUS in patients with acute recurrent pancreatitis of unknown aetiology. Dig Liver Dis. 2009;41(10):753-8.

40. Morris-Stiff G, Al-Allak A, Frost B, Lewis WG, Puntis MC, Roberts A. Does Endoscopic Ultrasound Have Anything to Offer in the Diagnosis of Idiopathic Acute Pancreatitis? JOP. 2009;10(2):143-6.

For peer review only

For peer review only - http://bmjopen.bmj.com/site/about/guidelines.xhtml

41. Norton SA, Alderson D. Endoscopic ultrasonography in the evaluation of idiopathic acute pancreatitis British Journal of Surgery. 2000;97:1650-5.

42. Queneau P-E, Zeeh S, Lapeyre V, Thibault P, Heyd B, Carayon P, et al. Feasibility of and Interest in Combined Endoscopic Ultrasonography and Biliary Drainage in Unexplained Acute Biliopancreatic Disorders. Dig Dis Sci. 2002;47(9):2020-4.

43. Rana SS, Bhasin DK, Rao C, Singh K. Role of endoscopic ultrasound in idiopathic acute pancreatitis with negative ultrasound, computed tomography, and magnetic resonance cholangiopancreatography. Ann Gastroenterol. 2012;25(2):133-7.

44. Alejandro Repiso Ortega M, Rafael Gómez-Rodríguez, MD, Marta Romero, MD, Soledad Fernández-Zapardiel, MD, María del Mar Céspedes, MD, and José-María Carrobles, MD. Prospective Comparison of Endoscopic Ultrasonography and Magnetic Resonance Cholangiopancreatography in the Etiological Diagnosis of ‘‘Idiopathic’’ Acute Pancreatitis Pancreas. 2011;40(2):289-94.

45. Tandon M, Topazian M. Endoscopic Ultrasound in Idiopathic Acute Pancreatitis. Am J Gastroenterol. 2001;96(3):705–9.

46. Thevenot A, Bournet B, Otal P, Canevet G, Moreau J, Buscail L. Endoscopic ultrasound and magnetic resonance cholangiopancreatography in patients with idiopathic acute pancreatitis. Dig Dis Sci. 2013;58(8):2361-8.

47. Lara LF, Levy MJ. Idiopathic Recurrent Acute Pancreatitis. MedGenMed. 2004;6(4):10.

48. Signoretti M, Baccini F, Piciucchi M, Iannicelli E, Valente R, Zerboni G, et al. Repeated Transabdominal Ultrasonography Is a Simple and Accurate Strategy to Diagnose a Biliary Etiology of Acute Pancreatitis. Pancreas. 2014;43(7):1106-10.

49. Meeralam Y, Al-Shammari K, Yaghoobi M. Diagnostic accuracy of EUS compared with MRCP in detecting choledocholithiasis: a meta-analysis of diagnostic test accuracy in head-to-head studies. Gastrointest Endosc. 2017;86(6):986-93.

50. Stolle M, Sack PM, Thomasius R. Binge drinking in childhood and adolescence: epidemiology, consequences, and interventions. Dtsch Arztebl Int. 2009;106(19):323-8.

For peer review only

For peer review only - http://bmjopen.bmj.com/site/about/guidelines.xhtml

51. Sadr Azodi O, Orsini N, Andren-Sandberg A, Wolk A. Effect of type of alcoholic beverage in causing acute pancreatitis. Br J Surg. 2011;98(11):1609-16.

52. Midanik LT. Drunkenness, feeling the effects and 5+ measures. Addiction. 1999;94(6):887- 97.

53. Nicolien J. Schepers OJB, Marc G. H. Besselink, Thomas L. Bollen, Marcel G. W. Dijkgraaf, Casper H. J. van Eijck, Paul Fockens, Erwin J. M. van Geenen, Janneke van Grinsven, Nora D. L. Hallensleben, Bettina E. Hansen, Hjalmar C. van Santvoort, Robin Timmer, Marie-Paule G. F. Anten, Clemens J. M. Bolwerk, Foke van Delft, Hendrik M. van Dullemen, G. Willemien Erkelens, Jeanin E. van Hooft, Robert Laheij, René W. M. van der Hulst, Jeroen M. Jansen, Frank J. G. M. Kubben, Sjoerd D. Kuiken, Lars E. Perk, Rogier J. J. de Ridder, Marno C. M. Rijk, Tessa E. H. Römkens, Erik J. Schoon, Matthijs P. Schwartz, B. W. Marcel Spanier, Adriaan C. I. T. L. Tan, Willem J. Thijs, Niels G. Venneman, Frank P. Vleggaar, Wim van de Vrie, Ben J. Witteman, Hein G. Gooszen, Marco J. Bruno and for the Dutch Pancreatitis Study Group. Early biliary decompression versus conservative treatment in acute biliary pancreatitis (APEC trial): study protocol for a randomized controlled trial. Trials. 2016;17(5).

54. Ratjen F, Döring G. Cystic fibrosis. The Lancet. 2003;361(9358):681-9.

55. Spanier B, Bruno MJ, Dijkgraaf MG. Incidence and mortality of acute and chronic pancreatitis in the Netherlands: a nationwide record-linked cohort study for the years 1995-2005. World J Gastroenterol. 2013;19(20):3018-26.

56. StatLine C. Households; size, position in household, January 1st 1995-2013 StatLine: Central Bureau for Statistics 2015 [Available from: http://statline.cbs.nl/Statweb/publication/?VW=T&DM=SLNL&PA=37312&D1=a&D2=0,5,10,(l-2)-l&HD=180221-1109&HDR=G1&STB=T.

57. Whitcomb DC. Genetic aspects of pancreatitis. Annu Rev Med. 2010;61:413-24.

58. Whitcomb DC. Hereditary diseases of the pancreas. In: Yamada T AD, Kaplowitz N, Laine L, Owyang C, Powell DW, editors, editor. Textbook of Gastroenterology. 4th ed. Philadelphia: Lippincott Williams & Wilkins; 2003. p. 2147–65.

For peer review only

For peer review only - http://bmjopen.bmj.com/site/about/guidelines.xhtml

59. Shane E, Dinaz I. Hypercalcemia: Pathogenesis, clinical manifestations, differential diagnosis and management. In: Favus M, editor. Primer on the metabolic bone diseases and disorders of mineral metabolism. 6th ed. Philadelpiha: Kippincott, Williams, and Wilkins; 2006. p. 26-176.

60. Berglund L, Brunzell JD, Goldberg AC, Goldberg IJ, Sacks F, Murad MH, et al. Evaluation and treatment of hypertriglyceridemia: an Endocrine Society clinical practice guideline. J Clin Endocrinol Metab. 2012;97(9):2969-89.

61. Nitsche C, Maertin S, Scheiber J, Ritter CA, Lerch MM, Mayerle J. Drug-induced pancreatitis. Curr Gastroenterol Rep. 2012;14(2):131-8.

62. Lankisch PG, Apte M, Banks PA. Acute pancreatitis. The Lancet. 2015;386(9988):85-96.

63. Tummala P, Tariq SH, Chibnall JT, Agarwal B. Clinical Predictors of Pancreatic Carcinoma Causing Acute Pancreatitis. Pancreas. 2013;42(1):108-13.

64. P. B. Cotton M, G. Lehman, MD, J. Vennes, MD, J. E. Geenen, MD, R. C. G. Russell, MD, W. C. Meyers, MD, C. Liguory, MD, N. Nicki, MD. Endoscopic sphincterotomy complications and their management: an attempt at consensus. Gastrointest Endosc. 1991;37(3):383-93.

65. Connor S. Defining post-operative pancreatitis as a new pancreatic specific complication following pancreatic resection. HPB (Oxford). 2016;18(8):642-51.

66. Booth FV FL. Pancreatoduodenal trauma. In: ed BJ, editor. Blunt multiple trauma. New York: Marcel Dekker; 1990. p. 497-509.

67. Ioana Smith M, Klaus Monkemuller, MD, PhD, and C. Mel Wilcox, MD, MSPH. Incidentally Identified Common Bile Duct Dilatation. A Systematic Review of Evaluation, Causes, and Outcome. J Clin Gastroenterol. 2015;49:810-5.

68. Mortelé KJ, Rocha TC, Streeter JL, Taylor AJ. Multimodality Imaging of Pancreatic and Biliary Congenital Anomalies. Radiographics. 2006;26(3):715-31.

For peer review only

Author Statement

Authors' contributions

DSU drafted the manuscript. HCT, RCV, SAB, MGB and JEvH co-authored the writing of the manuscript. DSU, RCV, SAB, MABo, MJB, PF, EJMvG, JWP, HCvS, FPV, MGB and JEvH were involved in the design of the study during several meetings of the Dutch Pancreatitis Study Group. NDHL, MPGFA, AB, RAB, MABr, LH, WLC, HMvD, BCvE, GWE, WLH, CVH, AI, LMK, SDK, LEP, RQ, TEHR, ACITLT, AYT, NGV, AMCJV, RLJvW and BJW critically assessed the study design, during several meetings, and edited the manuscript. All authors read and approved the final manuscript.

Funding statement

This work was supported by the Dutch Digestive Disease Foundation (*Maag Lever Darm Stichting*, grant number D17-25). The PICUS study is an investigator-initiated study. The sponsor had no influence on design, implementation and conduct of the study, as well as on collection, analysis and interpretation of data, construction of the manuscript and decision to publish.

Competing interests statement

The authors declare that they have no competing interests.

Data Availability Statement

The datasets used and/or analyzed during the current study are available from the corresponding author on reasonable request.

For peer review only - http://bmjopen.bmj.com/site/about/guidelines.xhtml

For peer review only

Figure legend

Overview of screening and study procedures. MRI = magnetic resonance imaging. MRCP = magnetic resonance cholangiopancreaticography. CRF = Case Report Form. EUS = endoscopic ultrasonography.

Table 1

**Standard diagnostic work-up**	
Detailed personal and family history, including questions on:	Alcohol use
	Recent ERCP
	Recent start or changes in use of drugs associated with acute pancreatitis
	Recent major abdominal trauma
	Recent abdominal surgery
	Familial and hereditary pancreatitis
	Cystic fibrosis-related pancreatitis
Laboratory tests, including:	Blood serum triglycerides level
	Blood serum calcium level, corrected for the blood serum albumin level
	Blood serum ALT level on admission
Imaging:	Transabdominal ultrasound, MRI or MRCP after clinical recovery

Table 1: Standard diagnostic work-up *Standard diagnostic work-up according to the 2013 IAP/APA evidence-based guideline on management of acute pancreatitis. A listing of the drugs considered to be associated with acute pancreatitis are listed in additional file 1. ERCP = endoscopic retrograde cholangiopancreaticography; ALT = alanine aminotransferase; MRI = magnetic resonance imaging; MRCP = magnetic resonance cholangiopancreaticography.*

For peer review only

For peer review only - http://bmjopen.bmj.com/site/about/guidelines.xhtml

649 Table 2

Etiology	Definition
Alcohol	> 4 units of alcohol in the 24 hours prior to start of abdominal complaints (50-52)
Biliary disease	1. A transient elevated ALT level of >2 times the upper limit of normal at diagnosis of acute pancreatitis (34), OR<br>2. Gallstones, microlithiasis and/or biliary sludge, OR<br>3. A dilated CBD of >8 mm in patients <76 years or >10 mm in patients >75 years at diagnosis of acute pancreatitis (53)
Cystic fibrosis	history of cystic fibrosis in the absence of another origin (54)
Familial	two or more direct blood-related family members (parents, children or siblings) who have had an episode of acute pancreatitis (55-57)
Hereditary	mutation in the PRSS1, SPINK1, CFTR, CTRC, CLDN2 or CPA1 gene, or direct family member (parents, children, siblings) with one or more of the above mentioned mutations and at least one direct family member who has (had) acute or chronic pancreatitis (57, 58)
Hypercalcemia	blood serum calcium level ≥12 mg/dl (3 mmol/l), corrected for serum albumin level, as first measured during admission (59)
Hypertriglyceridemia	blood serum triglyceride level of ≥1000 mg/dl (11.2 mmol/l) under fasting conditions, as first measured during admission (60)
Medication	use of drug(s) listed in additional file 1, which has or have been started or increased in dosage within a reasonable temporal sequence, in principle 1

For peer review only

	month before onset of pancreatitis, and has or have a positive dechallenge (a drug reaction that is confirmed by stopping the drug) (61, 62)
Neoplasm	Known hepatopancreatobiliary malignancy or known malignancy with metastases causing obstruction of the pancreatic duct (63)
ERCP	ERCP within 24 hours before diagnosis of pancreatitis (64)
Surgical	abdominal surgery within 24 hours prior to diagnosis of pancreatitis (65)
Trauma	typical blunt trauma to the upper abdomen and pancreatic trauma visible on imaging (66)

Table 2: potential etiologies and their definitions *Potential etiologies and their definitions. Side branch or mixed type intraductal papillary mucinous neoplasms without dilatation of the pancreatic duct and pancreas divisum will not be considered to be a causative factor for the pancreatitis episode. If imaging is not able to discriminate between gall bladder polyps or concrements, lesions smaller than 10 mm will not be considered an exclusion criterion. Lesions above 10 mm, irrespective of whether they are a polyp or a concrement, are an immediate indication for cholecystectomy, and these patients will be excluded from PICUS. ALT = alanine transaminase. CBD = common bile duct. ERCP = endoscopic retrograde cholangiopancreaticography.*

For peer review only

Table 3

Biliary pancreatitis	Presence of biliary stones, microlithiasis, or sludge
	Widened CBD, >8 mm in patients <76 years, or >10 mm in patients >75 years, in the absence of other CBD dilating factors (e.g. opioid use, distal stenosis, obstruction of external compression of CBD or papilla (67))
Chronic pancreatitis	Pancreatic calcifications
	> 4 of the following abnormal features of the pancreas:

For peer review only - http://bmjopen.bmj.com/site/about/guidelines.xhtml

	1. Enlarged gland size<br>2. Cysts<br>3. Echo-poor lesions (focal areas of reduced echogenicity)<br>4. Echo-rich lesions (> 3 mm in diameter)<br>5. Accentuation of lobular pattern<br>6. Increased duct wall echogenicity<br>7. Irregularity of the main pancreatic duct<br>8. Dilation of the main pancreatic duct > 3.5 mm (68)<br>9. Visible side branches<br>10. Calcifications of the pancreatic duct
Neoplasms	Definitive diagnosis of pathological tissue after histological or cytological evaluation of specimen of an anomaly observed during EUS, e.g. hyperplastic or malignant tissue, or auto-immune inflammatory disease
	Main duct IPMN or mixed type IPMN causing dilatation of the pancreatic duct

Table 3: positive imaging *Definition of positive imaging. For each diagnosis, presence of one of the separately mentioned abnormalities is required to be considered as positive imaging. Specimen is not required to be obtained during EUS. Anatomical anomalies (e.g. divisum) are not considered a certain etiology in first episode IAP and therefore not considered as positive imaging. CBD = common bile duct. EUS = endoscopic ultrasonography. IPMN = intraductal papillary mucinous neoplasm.*

For peer review only

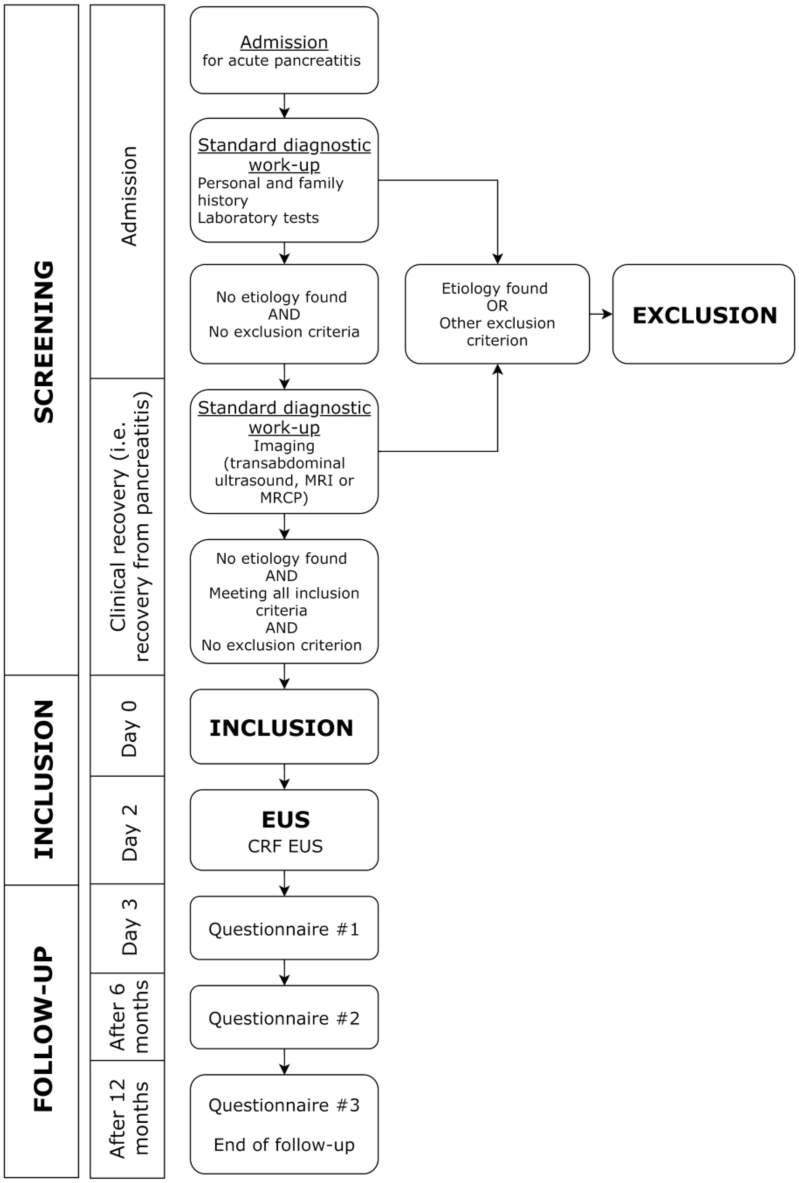

Overview of screening and study procedures. MRI = magnetic resonance imaging. MRCP = magnetic resonance cholangiopancreaticography. CRF = Case Report Form. EUS = endoscopic ultrasonography.

105x154mm (600 x 600 DPI)

Additional file 1: Table S1 Drugs associated with acute pancreatitis

<table>
<tr><th colspan="5">Drugs associated with acute pancreatitis</th></tr>
<tr><td>Acetaminophen</td><td>Cisplatin</td><td>Hydrochlorothiazide</td><td>Methyldopa</td><td rowspan="2">Pentavalent antimony compounds</td></tr>
<tr><td>Asparaginase</td><td>Cytarabine</td><td>Interferon alpha</td><td>Metronidazole</td></tr>
<tr><td>Azathioprine</td><td>Didanosine</td><td>Itraconazole</td><td>Octreotide</td><td>Phenformin</td></tr>
<tr><td>Bortezomib</td><td>Enalapril</td><td>Lamivudine</td><td>Olanzapine</td><td>Simvastatin</td></tr>
<tr><td>Capecitabine</td><td>Erythromycin</td><td>Mercaptopurine</td><td>Opiates</td><td>Steroids</td></tr>
<tr><td>Carbamazepine</td><td>Estrogens</td><td>Mesalazine</td><td>Oxyphenbutazone</td><td>Sulfasalazine</td></tr>
<tr><td>Cimetidine</td><td>Furosemide</td><td>Olsalazine</td><td>Pentamidine</td><td>co-trimoxazole</td></tr>
</table>

Drugs with a definite association with acute pancreatitis (1, 2)

References

1. Nitsche C, Maertin S, Scheiber J, Ritter CA, Lerch MM, Mayerle J. Drug-induced pancreatitis. Curr Gastroenterol Rep. 2012;14(2):131-8.
2. Lankisch PG, Apte M, Banks PA. Acute pancreatitis. The Lancet. 2015;386(9988):85-96.

For peer review only

Additional file 2: Relevant definitions

Acute pancreatitis: an acute inflammation of the pancreatic parenchyma, diagnosed when at least two of the three following characteristics are present (1):

1. Clinical features of acute pancreatitis, such as upper abdominal pain
2. Elevated serum amylase or lipase levels of at least three times the upper limit of normal (ULN)
3. Signs of acute pancreatitis on imaging

Note: no value of the required serum amylase or lipase level is provided as every participating center has a local laboratory, which is why each center may use different normal range values.

Idiopathic acute pancreatitis is considered to be present if no etiology is found in standard work-up, which comprises at least the following tests:

1. A detailed personal and family history, including questions on:
    a. Alcohol use
    b. Recent endoscopic retrograde cholangiopancreaticography (ERCP)
    c. Recent start of or changes in use of drugs associated with acute pancreatitis
    d. Recent major abdominal trauma
    e. Recent abdominal surgery
    f. Familial pancreatitis
    g. Hereditary pancreatitis
    h. Cystic fibrosis related pancreatitis

For peer review only

2. Laboratory tests, including:
    a. Blood serum triglycerides level on admission
    b. Blood serum calcium level, corrected for the serum albumin level, on admission
    c. Blood serum alanine transaminase (ALT) level on admission
3. Imaging via transabdominal ultrasound, magnetic resonance imaging (MRI) or magnetic resonance cholangiopancreaticography (MRCP) after clinical recovery

Note: side branch or mixed type intraductal papillary mucinous neoplasms (IPMN) without dilatation of the pancreatic duct will not be considered to be a causative factor for the pancreatitis episode.

Note: if the imaging is not able to discriminate between gall bladder polyps or concrements, lesions smaller than 10 mm will not be considered an exclusion criterion. Lesions above 10 mm, irrespective of whether they are a polyp or a concrement, are an immediate indication for cholecystectomy, and will be excluded from PICUS.

<ins>Alcoholic pancreatitis</ins>: pancreatitis caused by an excess intake of alcohol, diagnosed when biliary etiology is not demonstrated by standard work-up and the patient has indicated (either by direct or indirect personal history or by findings during physical examination) to have drank at least five units of alcohol in the 24 hours prior to start of abdominal complaints (or in asymptomatic acute pancreatitis: prior to diagnosis) (2-4)

<ins>Biliary pancreatitis</ins>: pancreatitis caused by biliary stones, microlithiasis or sludge, diagnosed when one of the following features is present:

1. A transient elevated ALT level of more than two times the ULN at diagnosis of acute pancreatitis (5)

For peer review only

2. Signs of presence of gallstones, microlithiasis or sludge on imaging, defined as follows:

    a. Gallstones, microlithiasis and/or biliary sludge, either in the gall bladder, ductus cysticus, intrahepatic bile ducts or in the common bile duct (CBD), and/or

    b. A CBD of more than eight mm in patients 75 years old or younger or more than ten mm in patients older than 75 years at diagnosis of acute pancreatitis (6)

Note: no value of the required serum ALT level is provided as the normal range values depend on the sex of the patient and as every participating center has a local laboratory, which is why each center may use different normal range values.

Chronic pancreatitis**:** a chronic inflammation of the pancreatic parenchyma, defined as typical clinical history of chronic pancreatitis (such as recurrent pancreatitis or abdominal pain, except for primary painless pancreatitis) and one or more of the following (7):

1. Pancreatic calcifications

2. Moderate or marked ductal lesions, defined as two or more of the following abnormal features on transabdominal ultrasound, computed tomography (CT) or MRI/MRCP, according to the Cambridge classification (8):

    a. Main pancreatic duct abnormalities, either enlargement or increased echogenicity of the duct wall (mandatory)

    b. Pancreatic enlargement

    c. Cavities

    d. Duct irregularities including intraductal fillings defects, calculi or duct obstruction

    e. Focal acute pancreatitis

For peer review only

f. Parenchymal heterogeneity

g. Irregularities of pancreatic head or body contour

3. Moderate or marked ductal lesions, defined as five or more of the following abnormal features on endoscopic ultrasonography (EUS):

a. Enlarged gland size

b. Cysts

c. Echo-poor lesions (focal areas of reduced echogenicity)

d. Echo-rich lesions (more than three mm in diameter)

e. Accentuation of lobular pattern (e.g., echo-poor normal parenchyma surrounded by hyperechoic strands)

f. Increased duct wall echogenicity

g. Irregularity of the main pancreatic duct (e.g., with narrowing of the duct)

h. Dilation of the main pancreatic duct

i. Visible side branches (e.g., with dilation)

j. Calcification (of the pancreatic duct)

4. Marked and persistent exocrine insufficiency defined as pancreatic steatorrhea markedly reduced by enzyme supplementation

5. Typical histology of an adequate histological specimen

Note: during initial diagnostic work-up during admission 'marked and persistent exocrine insufficiency' cannot be evaluated properly. Therefore this part of the definition of chronic pancreatitis will not be applicable during standard work-up. However, if the patient does show marked and persistent exocrine insufficiency during follow-up (either during the outpatient clinic visit after repeat

For peer review only

transabdominal ultrasound or after the EUS), this will be considered to be diagnostic for chronic pancreatitis. The same is applicable for histology of an adequate histological specimen: this is not part of standard work-up, however, if a typical histological specimen is obtained during follow-up, this will be considered to be diagnostic for chronic pancreatitis.

Cystic fibrosis: an autosomal recessive disorder caused by a mutation in the CFTR gene, resulting in defective chloride channels in epithelial cells, diagnosed by either a concentration in sweat of chloride greater than 60 mmol/L on repeated analysis, confirmation of a CFTR gene mutation, or both (9).

Cystic fibrosis related pancreatitis: pancreatitis caused by defective ductular and acinar pancreatic secretion, diagnosed when a patient with a history of cystic fibrosis presents with an acute pancreatitis in the absence of another origin (9).

Familial pancreatitis: acute pancreatitis from any cause that occurs in a family with an incidence that is greater than would be expected by chance alone, given the size of the family and the standardized incidence of pancreatitis within the Dutch population, defined as acute pancreatitis in patients who have two or more direct blood-related family members (parents, children or siblings) who have had an episode of acute pancreatitis (10-12).

Fever: a body temperature of 38.5˚C or higher.

Hereditary pancreatitis: otherwise unexplained pancreatitis in an individual from a family in which the pancreatitis phenotype appears to be inherited through a disease-causing gene mutation expressed in

For peer review only

an autosomal dominant pattern, defined as pancreatitis in patients with a known mutation in the PRSS1 gene, the SPINK1 gene, the CFTR gene, the CTRC gene, the CLDN2 gene or the CPA1 gene, or if the patient has a direct family member (parents, children, siblings) with one or more of the above mentioned mutations and has at least one direct family member who has had an episode of acute pancreatitis or has chronic pancreatitis (12, 13).

Hypercalcemic pancreatitis: acute pancreatitis caused by hypercalcemia and diagnosed when no signs of a biliary pancreatitis are found in standard work-up and the patient has a blood serum calcium level of at least 12 mg/dl or 3 mmol/l, corrected for the serum albumin level, as first measured during admission (14).

For peer review only

Hypertriglyceridemic pancreatitis: acute pancreatitis based on hypertriglyceridemia and diagnosed if a biliary etiology is not demonstrated by standard work-up and the patient has a blood serum triglyceride level of at least 1000 mg/dl (or 11.2 mmol/l) under fasting conditions, as first measured during admission (15).

Hypothermia: a body temperature of 35.9˚C or lower.

Infected (extra)pancreatic necrosis: presence of microorganisms in (extra-)pancreatic necrosis, confirmed by a positive culture obtained by means of fine needle aspiration or from the first drainage procedure or necrosectomy, the presence of gas in the (extra-)pancreatic collection on CT, or the presence of clinical signs of persistent sepsis or progressive clinical deterioration despite maximal support on the intensive care unit (ICU) without other causes for infection (ruled out should be:

For peer review only - http://bmjopen.bmj.com/site/about/guidelines.xhtml

pneumonia, urinary tract infection, wound infection, endocarditis, abdominal sepsis or any other infection which could be suspected based on the individual patient's clinical presentation) (16).

Medication associated pancreatitis: acute pancreatitis is considered to be caused by drugs when a biliary cause is not demonstrated by standard work-up, the patient uses one or multiple drug(s) listed in table S1 in additional file 1, the drug has been started or increased in dosage within a reasonable temporal sequence, in principle 1 month before the onset of the pancreatitis, and has a positive dechallenge (a drug reaction that is confirmed by stopping the drug) (17, 18).

Microlithiasis: stones or concrements, smaller than four mm, in the gall bladder or the bile ducts (19).

Murphy's sign: the phenomenon where compression of the right upper quadrant causes the patient to catch their breath due to pain when taking a deep breath (20).

Pancreas divisum: a congenital malformation of the main pancreatic duct (Wirsung's duct) with two separate ducts (a separate ventral duct of Wirsung and a dorsal duct of Santorini) as opposed to one main duct (of Wirsung) (21).

Positive imaging: positive imaging is defined as imaging during which a definitive cause for the acute pancreatitis episode can be found; or during which abnormalities are visualized constituting a definitive cause, after obtaining tissue and pathological examination. So, if during EUS ductal abnormalities are found, yet not enough to make a certain diagnosis of chronic pancreatitis according to the M-ANNHEIM classification (7), this imaging is considered to be negative, even though it did show abnormalities. This approach is chosen because the aim of this study is to determine the rate of which

For peer review only

EUS can find a causative factor for a previous acute pancreatitis episode. For the same reason, finding of an anatomical abnormality after a first episode of acute pancreatitis is not scored as positive imaging. An overview of the exact findings scored as positive imaging is provided in table 3 of the main manuscript.

Post-ERCP pancreatitis: pancreatitis caused by mechanical injury from instrumentation and hydrostatic injury from contrast injection during ERCP, diagnosed if a patient develops a pancreatitis within 24 hours of an ERCP without indications of another origin (22).

Postoperative pancreatitis: pancreatitis caused by perioperative hypoperfusion of the pancreas, diagnosed if a patient develops a pancreatitis within 24 hours of abdominal surgery in the absence of indications for another origin (23).

Posttraumatic pancreatitis: pancreatitis caused by pancreatic injury due to trauma to the abdomen, diagnosed when the patient describes a typical blunt trauma to the upper abdomen and pancreatic trauma is visible on imaging (24).

Recurrence rate: the risk of a recurrent episode of acute pancreatitis.

Sludge: solid material which results from the slow settling of particles dispersed in bile (19).

For peer review only

Standard work-up:

1. A detailed personal and family history, including questions on:
    a. Alcohol use
    b. Recent ERCP
    c. Recent start of or changes in use of drugs associated with acute pancreatitis
    d. Recent major abdominal trauma
    e. Recent abdominal surgery
    f. Familial pancreatitis
    g. Hereditary pancreatitis
    h. Cystic fibrosis related pancreatitis
2. Laboratory tests, including:
    a. Blood serum triglycerides level, first measured during admission
    b. Blood serum calcium level, corrected for the serum albumin level, first measured during admission
    c. Blood serum ALT level on admission
3. Imaging via transabdominal ultrasound, MRI or MRCP after clinical recovery

Biliary events: acute cholecystitis; biliary colic's requiring readmission; biliary pancreatitis; cholangitis; or obstructive choledocholithiasis needing ERCP.

For peer review only

Acute cholecystitis: an acute inflammation of the gall bladder, diagnosed when one item in A, B and C is present:

A) Local signs of inflammation

   1. Murphy's' sign, or
   2. Right upper abdominal quadrant mass, pain or tenderness

B) Systemic signs of inflammation

   1. Fever or hypothermia, or
   2. Elevated C-reactive protein CRP), or
   3. Elevated white blood cell count

C) Imaging findings characteristic of acute cholecystitis (25, 26)

Note: acute cholecystitis and cholangitis are defined according to the Tokyo classification which defines fever as a body temperature of 38˚C or higher; however, fever will be defined in this study as hyperthermia of 38.5˚C or higher and hypothermia will be added as a systemic sign of inflammation, as this more accurately reflects clinical practice in the Netherlands.

Biliary colic: upper abdominal pain (either right upper quadrant or epigastric pain) lasting at least 30 minutes, often associated with restlessness (27).

Cholangitis: an inflammation of the bile duct(s), diagnosed when one item in each of the following categories is present:

1. Systemic inflammation

   a. Fever, hypothermia and/or shaking chills

For peer review only

b. Laboratory data: evidence of inflammatory response (abnormal white blood cell counts (defined as smaller than 4,000/µl or larger than 10,000/µl), increase of serum CRP levels (defined as 1 mg/dl or higher), and other changes indicating inflammation)

2. Cholestasis

a. Jaundice (defined as a total bilirubin of 2 mg/dl or higher)

b. Laboratory data: abnormal liver function tests (increased serum alkaline phosphatase, gamma-glutamyltransferase (gamma-GT), aspartate transaminase (AST) and ALT levels (defined as more than 1.5 times the ULN))

3. Imaging

a. Biliary dilatation

b. Evidence of the etiology on imaging (stricture, stone, stent etc.) (25)

Note: acute cholecystitis and cholangitis are defined according to the Tokyo classification which defines fever as a body temperature of 38˚C or higher; however, fever will be defined in this study as hyperthermia of 38.5˚C or higher and hypothermia will be added as a systemic sign of inflammation, as this more accurately reflects clinical practice in the Netherlands.

<u>Obstructive choledocholithiasis</u>: presence of gallstones, microlithiasis or biliary sludge in the CBD on imaging, requiring an ERCP, according to the treating physician.

For peer review only

References

1. Banks PA, Bollen TL, Dervenis C, Gooszen HG, Johnson CD, Sarr MG, et al. Classification of acute pancreatitis--2012: revision of the Atlanta classification and definitions by international consensus. Gut. 2013;62(1):102-11.

2. Stolle M, Sack PM, Thomasius R. Binge drinking in childhood and adolescence: epidemiology, consequences, and interventions. Dtsch Arztebl Int. 2009;106(19):323-8.

3. Sadr Azodi O, Orsini N, Andren-Sandberg A, Wolk A. Effect of type of alcoholic beverage in causing acute pancreatitis. Br J Surg. 2011;98(11):1609-16.

4. Midanik LT. Drunkenness, feeling the effects and 5+ measures. Addiction. 1999;94(6):887- 97.

5. B. J. Ammori BB, P. Lewis, and S. A. Roberts. The Biochemical Detection of Biliary Etiology of Acute Pancreatitis on Admission: A Revisit in the Modern Era of Biliary Imaging. Pancreas. 2003;26(2):e32–e5.

6. Nicolien J. Schepers OJB, Marc G. H. Besselink, Thomas L. Bollen, Marcel G. W. Dijkgraaf, Casper H. J. van Eijck, Paul Fockens, Erwin J. M. van Geenen, Janneke van Grinsven, Nora D. L. Hallensleben, Bettina E. Hansen, Hjalmar C. van Santvoort, Robin Timmer, Marie-Paule G. F. Anten, Clemens J. M. Bolwerk, Foke van Delft, Hendrik M. van Dullemen, G. Willemien Erkelens, Jeanin E. van Hooft, Robert Laheij, René W. M. van der Hulst, Jeroen M. Jansen, Frank J. G. M. Kubben, Sjoerd D. Kuiken, Lars E. Perk, Rogier J. J. de Ridder, Marno C. M. Rijk, Tessa E. H. Römkens, Erik J. Schoon, Matthijs P. Schwartz, B. W. Marcel Spanier, Adriaan C. I. T. L. Tan, Willem J. Thijs, Niels G. Venneman, Frank P. Vleggaar, Wim van de Vrie, Ben J. Witteman, Hein G. Gooszen, Marco J. Bruno and for the Dutch Pancreatitis Study Group. Early biliary decompression versus conservative treatment in acute biliary pancreatitis (APEC trial): study protocol for a randomized controlled trial. Trials. 2016;17(5).

7. Schneider A, Lohr JM, Singer MV. The M-ANNHEIM classification of chronic pancreatitis: introduction of a unifying classification system based on a review of previous classifications of the disease. J Gastroenterol. 2007;42(2):101-19.

8. Sarner M, Cotton PB. Classification of pancreatitis. Gut. 1984;25:756-9.

9. Ratjen F, Döring G. Cystic fibrosis. The Lancet. 2003;361(9358):681-9.

For peer review only

10. Spanier B, Bruno MJ, Dijkgraaf MG. Incidence and mortality of acute and chronic pancreatitis in the Netherlands: a nationwide record-linked cohort study for the years 1995-2005. World J Gastroenterol. 2013;19(20):3018-26.

11. StatLine C. Households; size, position in household, January 1st 1995-2013 StatLine: Central Bureau for Statistics 2015 [Available from: http://statline.cbs.nl/Statweb/publication/?VW=T&DM=SLNL&PA=37312&D1=a&D2=0,5,10,(l-2)-l&HD=180221-1109&HDR=G1&STB=T.

12. Whitcomb DC. Genetic aspects of pancreatitis. Annu Rev Med. 2010;61:413-24.

13. Whitcomb DC. Hereditary diseases of the pancreas. In: Yamada T AD, Kaplowitz N, Laine L, Owyang C, Powell DW, editors, editor. Textbook of Gastroenterology. 4th ed. Philadelphia: Lippincott Williams & Wilkins; 2003. p. 2147–65.

14. Shane E, Dinaz I. Hypercalcemia: Pathogenesis, clinical manifestations, differential diagnosis and management. In: Favus M, editor. Primer on the metabolic bone diseases and disorders of mineral metabolism. 6th ed. Philadelpiha: Kippincott, Williams, and Wilkins; 2006. p. 26-176.

15. Berglund L, Brunzell JD, Goldberg AC, Goldberg IJ, Sacks F, Murad MH, et al. Evaluation and treatment of hypertriglyceridemia: an Endocrine Society clinical practice guideline. J Clin Endocrinol Metab. 2012;97(9):2969-89.

16. van Brunschot S, van Grinsven J, van Santvoort HC, Bakker OJ, Besselink MG, Boermeester MA, et al. Endoscopic or surgical step-up approach for infected necrotising pancreatitis: a multicentre randomised trial. The Lancet. 2018;391(10115):51-8.

17. Nitsche C, Maertin S, Scheiber J, Ritter CA, Lerch MM, Mayerle J. Drug-induced pancreatitis. Curr Gastroenterol Rep. 2012;14(2):131-8.

18. Lankisch PG, Apte M, Banks PA. Acute pancreatitis. The Lancet. 2015;386(9988):85-96.

19. Jungst C, Kullak-Ublick GA, Jungst D. Gallstone disease: Microlithiasis and sludge. Best Pract Res Clin Gastroenterol. 2006;20(6):1053-62.

20. Murphy JB. The diagnosis of gall-stones. Am Med News. 1903;82:825.33.

21. PB C. Congenital anomaly of pancreas divisum as cause of obstructive pain and pancreatitis. Gut. 1980;21:105-14.

For peer review only

22. P. B. Cotton M, G. Lehman, MD, J. Vennes, MD, J. E. Geenen, MD, R. C. G. Russell, MD, W. C. Meyers, MD, C. Liguory, MD, N. Nicki, MD. Endoscopic sphincterotomy complications and their management: an attempt at consensus. Gastrointest Endosc. 1991;37(3):383-93.

23. Connor S. Defining post-operative pancreatitis as a new pancreatic specific complication following pancreatic resection. HPB (Oxford). 2016;18(8):642-51.

24. Booth FV FL. Pancreatoduodenal trauma. In: ed BJ, editor. Blunt multiple trauma. New York: Marcel Dekker; 1990. p. 497-509.

25. Miura F, Okamoto K, Takada T, Strasberg SM, Asbun HJ, Pitt HA, et al. Tokyo Guidelines 2018: initial management of acute biliary infection and flowchart for acute cholangitis. J Hepatobiliary Pancreat Sci. 2018;25(1):31-40.

26. Masamichi Yokoe TT, Steven M. Strasberg, Joseph S. Solomkin, Toshihiko Mayumi, Harumi Gomi, Henry A. Pitt, O. James Garden, Seiki Kiriyama, Jiro Hata, Toshifumi Gabata, Masahiro Yoshida, Fumihiko Miura, Kohji Okamoto, Toshio Tsuyuguchi, Takao Itoi, Yuichi Yamashita, Christos Dervenis, Angus C. W. Chan, Wan-Yee Lau, Avinash N. Supe, Giulio Belli, Serafin C. Hilvano, Kui-Hin Liau, Myung-Hwan Kim, Sun-Whe Kim, Chen-Guo Ker. TG13 diagnostic criteria and severity grading of acute cholecystitis (with videos). J Hepatobiliary Pancreat Sci. 2013;20:35-46.

27. EB French WR. Biliary and renal colic. Br Med J. 1963:135-8.

For peer review only

SPIRIT

STANDARD PROTOCOL ITEMS: RECOMMENDATIONS FOR INTERVENTIONAL TRIALS

SPIRIT 2013 Checklist: Recommended items to address in a clinical trial protocol and related documents*

Section/item	ItemNo	Description	Addressed on page number
**Administrative information**			
Title	1	Descriptive title identifying the study design, population, interventions, and, if applicable, trial acronym	1
Trial registration	2a	Trial identifier and registry name. If not yet registered, name of intended registry	7
	2b	All items from the World Health Organization Trial Registration Data Set	1, 3, 7, 9-14, 22, 23
Protocol version	3	Date and version identifier	1
Funding	4	Sources and types of financial, material, and other support	23
Roles and responsibilities	5a	Names, affiliations, and roles of protocol contributors	1-3, 23
	5b	Name and contact information for the trial sponsor	24
	5c	Role of study sponsor and funders, if any, in study design; collection, management, analysis, and interpretation of data; writing of the report; and the decision to submit the report for publication, including whether they will have ultimate authority over any of these activities	23

For peer review only - http://bmjopen.bmj.com/site/about/guidelines.xhtml

	5d	Composition, roles, and responsibilities of the coordinating centre, steering committee, endpoint adjudication committee, data management team, and other individuals or groups overseeing the trial, if applicable (see Item 21a for data monitoring committee)	24-26
**Introduction**			
Background and rationale	6a	Description of research question and justification for undertaking the trial, including summary of relevant studies (published and unpublished) examining benefits and harms for each intervention	8, 9
	6b	Explanation for choice of comparators	Not applicable
Objectives	7	Specific objectives or hypotheses	9
Trial design	8	Description of trial design including type of trial (eg, parallel group, crossover, factorial, single group), allocation ratio, and framework (eg, superiority, equivalence, noninferiority, exploratory)	9
**Methods: Participants, interventions, and outcomes**			
Study setting	9	Description of study settings (eg, community clinic, academic hospital) and list of countries where data will be collected. Reference to where list of study sites can be obtained	9
Eligibility criteria	10	Inclusion and exclusion criteria for participants. If applicable, eligibility criteria for study centres and individuals who will perform the interventions (eg, surgeons, psychotherapists)	10, 11
Interventions	11a	Interventions for each group with sufficient detail to allow replication, including how and when they will be administered	11
	11b	Criteria for discontinuing or modifying allocated interventions for a given trial participant (eg, drug dose change in response to harms, participant request, or improving/worsening disease)	Not applicable

For peer review only - http://bmjopen.bmj.com/site/about/guidelines.xhtml

	11c	Strategies to improve adherence to intervention protocols, and any procedures for monitoring adherence (eg, drug tablet return, laboratory tests)	Not applicable
	11d	Relevant concomitant care and interventions that are permitted or prohibited during the trial	9
Outcomes	12	Primary, secondary, and other outcomes, including the specific measurement variable (eg, systolic blood pressure), analysis metric (eg, change from baseline, final value, time to event), method of aggregation (eg, median, proportion), and time point for each outcome. Explanation of the clinical relevance of chosen efficacy and harm outcomes is strongly recommended	12-14
Participant timeline	13	Time schedule of enrolment, interventions (including any run-ins and washouts), assessments, and visits for participants. A schematic diagram is highly recommended (see Figure)	Figure
Sample size	14	Estimated number of participants needed to achieve study objectives and how it was determined, including clinical and statistical assumptions supporting any sample size calculations	14
Recruitment	15	Strategies for achieving adequate participant enrolment to reach target sample size	9
**Methods: Assignment of interventions (for controlled trials)**			
Allocation:			
Sequence generation	16a	Method of generating the allocation sequence (eg, computer-generated random numbers), and list of any factors for stratification. To reduce predictability of a random sequence, details of any planned restriction (eg, blocking) should be provided in a separate document that is unavailable to those who enrol participants or assign interventions	Not applicable
Allocation concealment mechanism	16b	Mechanism of implementing the allocation sequence (eg, central telephone; sequentially numbered, opaque, sealed envelopes), describing any steps to conceal the sequence until interventions are assigned	Not applicable

Implementation	16c	Who will generate the allocation sequence, who will enrol participants, and who will assign participants to interventions	Not applicable
Blinding (masking)	17a	Who will be blinded after assignment to interventions (eg, trial participants, care providers, outcome assessors, data analysts), and how	Not applicable
	17b	If blinded, circumstances under which unblinding is permissible, and procedure for revealing a participant's allocated intervention during the trial	Not applicable
**Methods: Data collection, management, and analysis**			
Data collection methods	18a	Plans for assessment and collection of outcome, baseline, and other trial data, including any related processes to promote data quality (eg, duplicate measurements, training of assessors) and a description of study instruments (eg, questionnaires, laboratory tests) along with their reliability and validity, if known. Reference to where data collection forms can be found, if not in the protocol	11, 14, 15
	18b	Plans to promote participant retention and complete follow-up, including list of any outcome data to be collected for participants who discontinue or deviate from intervention protocols	14, 15
Data management	19	Plans for data entry, coding, security, and storage, including any related processes to promote data quality (eg, double data entry; range checks for data values). Reference to where details of data management procedures can be found, if not in the protocol	11, 14, 15
Statistical methods	20a	Statistical methods for analysing primary and secondary outcomes. Reference to where other details of the statistical analysis plan can be found, if not in the protocol	15-17
	20b	Methods for any additional analyses (eg, subgroup and adjusted analyses)	15-17
	20c	Definition of analysis population relating to protocol non-adherence (eg, as randomised analysis), and any statistical methods to handle missing data (eg, multiple imputation)	15
**Methods: Monitoring**			

For peer review only - http://bmjopen.bmj.com/site/about/guidelines.xhtml

Data monitoring	21a	Composition of data monitoring committee (DMC); summary of its role and reporting structure; statement of whether it is independent from the sponsor and competing interests; and reference to where further details about its charter can be found, if not in the protocol. Alternatively, an explanation of why a DMC is not needed	22
	21b	Description of any interim analyses and stopping guidelines, including who will have access to these interim results and make the final decision to terminate the trial	Not applicable
Harms	22	Plans for collecting, assessing, reporting, and managing solicited and spontaneously reported adverse events and other unintended effects of trial interventions or trial conduct	14, 15
Auditing	23	Frequency and procedures for auditing trial conduct, if any, and whether the process will be independent from investigators and the sponsor	22
**Ethics and dissemination**			
Research ethics approval	24	Plans for seeking research ethics committee/institutional review board (REC/IRB) approval	22
Protocol amendments	25	Plans for communicating important protocol modifications (eg, changes to eligibility criteria, outcomes, analyses) to relevant parties (eg, investigators, REC/IRBs, trial participants, trial registries, journals, regulators)	22
Consent or assent	26a	Who will obtain informed consent or assent from potential trial participants or authorised surrogates, and how (see Item 32)	22
	26b	Additional consent provisions for collection and use of participant data and biological specimens in ancillary studies, if applicable	Not applicable
Confidentiality	27	How personal information about potential and enrolled participants will be collected, shared, and maintained in order to protect confidentiality before, during, and after the trial	11, 14-17
Declaration of interests	28	Financial and other competing interests for principal investigators for the overall trial and each study site	22

For peer review only - http://bmjopen.bmj.com/site/about/guidelines.xhtml

Access to data	29	Statement of who will have access to the final trial dataset, and disclosure of contractual agreements that limit such access for investigators	22
Ancillary and post-trial care	30	Provisions, if any, for ancillary and post-trial care, and for compensation to those who suffer harm from trial participation	Not applicable
Dissemination policy	31a	Plans for investigators and sponsor to communicate trial results to participants, healthcare professionals, the public, and other relevant groups (eg, via publication, reporting in results databases, or other data sharing arrangements), including any publication restrictions	23
	31b	Authorship eligibility guidelines and any intended use of professional writers	23
	31c	Plans, if any, for granting public access to the full protocol, participant-level dataset, and statistical code	22
**Appendices**			
Informed consent materials	32	Model consent form and other related documentation given to participants and authorised surrogates	Available upon request
Biological specimens	33	Plans for collection, laboratory evaluation, and storage of biological specimens for genetic or molecular analysis in the current trial and for future use in ancillary studies, if applicable	Not applicable

*It is strongly recommended that this checklist be read in conjunction with the SPIRIT 2013 Explanation & Elaboration for important clarification on the items. Amendments to the protocol should be tracked and dated. The SPIRIT checklist is copyrighted by the SPIRIT Group under the Creative Commons "Attribution-NonCommercial-NoDerivs 3.0 Unported" license.

For peer review only

BMJ Open

The role of endoscopic ultrasonography in the diagnostic work-up of idiopathic acute pancreatitis (PICUS): study protocol for a nationwide prospective cohort study

Journal:	*BMJ Open*
Manuscript ID	bmjopen-2019-035504.R1
Article Type:	Protocol
Date Submitted by the Author:	02-Mar-2020

Complete List of Authors:	Umans, Devica; Amsterdam University Medical Centres, Department of Gastroenterology and Hepatology; Saint Antonius Hospital, Research and Development<br>Timmerhuis, Hester; Saint Antonius Hospital, Department of Surgery; Saint Antonius Hospital, Research and Development<br>Hallensleben, Nora ; Erasmus Medical Center, Department of Gastroenterology and Hepatology; Saint Antonius Hospital, Research and Development<br>Bouwense, Stefan; Maastricht UMC+, Department of Surgery<br>Anten, Marie-Paule; Franciscus Gasthuis en Vlietland, Department of Gastroenterology and Hepatology<br>Bhalla, Abha; HagaZiekenhuis, Department of Gastroenterology and Hepatology<br>Bijlsma, Rina; Martini Ziekenhuis, Department of Gastroenterology and Hepatology<br>Boermeester, Marja; Amsterdam University Medical Centres, Department of Surgery<br>Brink, Menno; Meander MC, Department of Gastroenterology and Hepatology<br>Hol, Lieke; Maasstad Hospital, Department of Gastroenterology and Hepatology<br>Bruno, Marco; Erasmus Medical Center, Department of Gastroenterology and Hepatology<br>Curvers, Wouter; Catharina Hospital, Department of Gastroenterology and Hepatology<br>van Dullemen, Hendrik; UMCG, Department of Gastroenterology and Hepatology<br>van Eijck, Brechje; Spaarne Gasthuis, Department of Gastroenterology and Hepatology<br>Erkelens, G; Gelre Ziekenhuizen, Department of Gastroenterology and Hepatology<br>Fockens, Paul; Amsterdam University Medical Centres, Department of Gastroenterology and Hepatology<br>van Geenen, Erwin; Radboudumc<br>Hazen, Wouter; Elisabeth-TweeSteden Ziekenhuis, Department of Gastroenterology and Hepatology<br>Hoge, Chantal; Maastricht UMC+, Department of Gastroenterology and Hepatology<br>Inderson, Akin; LUMC, Department of Gastroenterology and Hepatology

	Kager, Liesbeth; Noordwest Ziekenhuisgroep, Department of Gastroenterology and Hepatology<br>Kuiken, Sjoerd; OLVG, Department of Gastroenterology and Hepatology<br>Perk, Lars; Medisch Centrum Haaglanden, Department of Gastroenterology and Hepatology<br>Poley, Jan-Werner<br>Quispel, Rutger; Reinier de Graaf Groep, Department of Gastroenterology and Hepatology<br>Römkens, Tessa; Jeroen Bosch Hospital, Department of Gastroenteroloy and Hepatology<br>van Santvoort, Hjalmar ; University Medical Center Utrecht; Saint Antonius Hospital, Department of Surgery<br>Tan, Adriaan; Canisius Wilhelmina Hospital, Department of Gastroenterology and Hepatology<br>Thijssen, Annemieke; Albert Schweitzer Ziekenhuis, Department of Gastroenterology and Hepatology<br>Venneman, Niels ; Medisch Spectrum Twente, Department of Gastroenterology and Hepatology<br>Vleggaar, Frank; Universitair Medisch Centrum Utrecht<br>Voorburg, Annet ; Diakonessenhuis Utrecht Zeist Doorn, Department of Gastroenterology and Hepatology<br>van Wanrooij, Roy; Amsterdam University Medical Centres, Department of Gastroenterology and Hepatology<br>Witteman, Ben; Ziekenhuis Gelderse Vallei, Department of Gastroenterology and Hepatology<br>Verdonk, Robert; Saint Antonius Hospital, Department of Gastroenterology and Hepatology<br>Besselink, Marc; Amsterdam University Medical Centres, Department of Surgery<br>van Hooft, Jeanin; AMC
**Primary Subject Heading**:	Gastroenterology and hepatology
Secondary Subject Heading:	Evidence based practice, Research methods
Keywords:	Endoscopy < GASTROENTEROLOGY, Hepatobiliary disease < GASTROENTEROLOGY, Pancreatic disease < GASTROENTEROLOGY

SCHOLARONE™
Manuscripts

I, the Submitting Author has the right to grant and does grant on behalf of all authors of the Work (as defined in the below author licence), an exclusive licence and/or a non-exclusive licence for contributions from authors who are: i) UK Crown employees; ii) where BMJ has agreed a CC-BY licence shall apply, and/or iii) in accordance with the terms applicable for US Federal Government officers or employees acting as part of their official duties; on a worldwide, perpetual, irrevocable, royalty-free basis to BMJ Publishing Group Ltd ("BMJ") its licensees and where the relevant Journal is co-owned by BMJ to the co-owners of the Journal, to publish the Work in this journal and any other BMJ products and to exploit all rights, as set out in our licence.

The Submitting Author accepts and understands that any supply made under these terms is made by BMJ to the Submitting Author unless you are acting as an employee on behalf of your employer or a postgraduate student of an affiliated institution which is paying any applicable article publishing charge ("APC") for Open Access articles. Where the Submitting Author wishes to make the Work available on an Open Access basis (and intends to pay the relevant APC), the terms of reuse of such Open Access shall be governed by a Creative Commons licence – details of these licences and which Creative Commons licence will apply to this Work are set out in our licence referred to above.

Other than as permitted in any relevant BMJ Author's Self Archiving Policies, I confirm this Work has not been accepted for publication elsewhere, is not being considered for publication elsewhere and does not duplicate material already published. I confirm all authors consent to publication of this Work and authorise the granting of this licence.

For peer review only

The role of endoscopic ultrasonography in the diagnostic work-up of idiopathic acute pancreatitis (PICUS): study protocol for a nationwide prospective cohort study

Devica S Umans[1, 2], Hester C Timmerhuis[2, 3], Nora DL Hallensleben[2, 4], Stefan A Bouwense[5], Marie-Paule GF Anten[6], Abha Bhalla[7], Rina A Bijlsma[8], Marja A Boermeester[9], Menno A Brink[10], Lieke Hol[11], Marco J Bruno[4], Wouter L Curvers[12], Hendrik M van Dullemen[13], Brechje C van Eijck[14], G Willemien Erkelens[15], Paul Fockens[1], Erwin JM van Geenen[16], Wouter L Hazen[17], Chantal V Hoge[18], Akin Inderson[19], Liesbeth M Kager[20], Sjoerd D Kuiken[21], Lars E Perk[22], Jan-Werner Poley[4], Rutger Quispel[23], Tessa EH Römkens[24], Hjalmar C van Santvoort[3, 25], Adriaan CITL Tan[26], Annemieke Y Thijssen[27], Niels G Venneman[28], Frank P Vleggaar[29], Annet MCJ Voorburg[30], Roy LJ van Wanrooij[31], Ben J Witteman[32], Robert C Verdonk[33], Marc G Besselink[8], Jeanin E van Hooft[1], for the Dutch Pancreatitis Study Group

[1] Department of Gastroenterology and Hepatology, Amsterdam Gastroenterology and Metabolism, Amsterdam UMC, University of Amsterdam, Amsterdam, the Netherlands

[2] Department of Research and Development, St. Antonius Hospital, Nieuwegein, the Netherlands

[3] Department of Surgery, St. Antonius Hospital, Nieuwegein, the Netherlands

[4] Department of Gastroenterology and Hepatology, Erasmus Medical Center, Rotterdam, the Netherlands

[5] Department of Surgery, Maastricht UMC+, Maastricht, the Netherlands

[6] Department of Gastroenterology and Hepatology, Franciscus Gasthuis & Vlietland, Rotterdam, the Netherlands

[7] Department of Gastroenterology and Hepatology, Haga Hospital, Den Haag, the Netherlands

[8] Department of Gastroenterology and Hepatology, Martini Hospital, Groningen, the Netherlands

[9] Department of Surgery, Amsterdam UMC, University of Amsterdam, Amsterdam, the Netherlands

For peer review only

[10] Department of Gastroenterology and Hepatology, Meander Medical Center, Amersfoort, the Netherlands

[11] Department of Gastroenterology and Hepatology, Maasstad Hospital, Rotterdam, the Netherlands

[12] Department of Gastroenterology and Hepatology, Catharina Hospital, Eindhoven, the Netherlands

[13] Department of Gastroenterology and Hepatology, University Medical Center Groningen, Groningen, the Netherlands

[14] Department of Gastroenterology and Hepatology, Spaarne Gasthuis, Haarlem, the Netherlands

[15] Department of Gastroenterology and Hepatology, Gelre Hospital, Apeldoorn, the Netherlands

[16] Department of Gastroenterology and Hepatology, Radboud university medical center, Nijmegen, the Netherlands

[17] Department of Gastroenterology and Hepatology, Elisabeth-Tweesteden Hospital, Tilburg, the Netherlands

[18] Department of Gastroenterology and Hepatology, Maastricht University Medical Center, Maastricht, the Netherlands

[19] Department of Gastroenterology and Hepatology, Leiden University Medical Center, Leiden, the Netherlands

[20] Department of Gastroenterology and Hepatology, Noordwest Ziekenhuisgroep, Alkmaar, the Netherlands

[21] Department of Gastroenterology and Hepatology, OLVG, Amsterdam, the Netherlands

[22] Department of Gastroenterology and Hepatology, Medical Center Haaglanden, Den Haag, the Netherlands

[23] Department of Gastroenterology and Hepatology, Reinier de Graaf Gasthuis, Delft, the Netherlands

[24] Department of Gastroenterology and Hepatology, Jeroen Bosch Hospital, Den Bosch, the Netherlands

[25] Department of Surgery, UMC Utrecht, Utrecht, the Netherlands

[26] Department of Gastroenterology and Hepatology, Canisius-Wilhelmina Hospital, Nijmegen, the Netherlands

[27] Department of Gastroenterology and Hepatology, Albert Schweitzer Hospital, Dordrecht, the Netherlands

[28] Department of Gastroenterology and Hepatology, Medisch Spectrum Twente, Enschede, the Netherlands

[29] Department of Gastroenterology and Hepatology, University Medical Center Utrecht, the Netherlands

[30] Department of Gastroenterology and Hepatology, Diakonessenhuis, Utrecht, the Netherlands

[31] Department of Gastroenterology and Hepatology, Amsterdam Gastroenterology and Metabolism, Amsterdam UMC, Vrije Universiteit van Amsterdam, Amsterdam, the Netherlands

[32] Department of Gastroenterology and Hepatology, Hospital Gelderse Vallei, Ede, the Netherlands

For peer review only

For peer review only - http://bmjopen.bmj.com/site/about/guidelines.xhtml

[33] Department of Gastroenterology and Hepatology, St. Antonius Hospital, Nieuwegein, the Netherlands

Correspondence

Dr. Jeanin E. van Hooft, MD, PhD, MBA

Amsterdam UMC, location AMC

Department of Gastroenterology and Hepatology, C2-115

PO Box 22660

1100 DD Amsterdam

The Netherlands

Telephone: +31 20 566 7918

E-mail: j.e.vanhooft@amsterdamumc.nl / j.e.van_hooft@lumc.nl

Correspondence during review phase

Devica S Umans

Telephone: +31 88 320 7051

E-mail: d.s.umans@amsterdamumc.nl

List of abbreviations

ALT = alanine aminotransferase

BMI = body mass index

For peer review only

CI = confidence interval

CRF = case report form

CT = computed tomography

ERCP = endoscopic retrograde cholangiopancreaticography

EUS = endoscopic ultrasonography

GRADE = Grading of Recommendations Assessment, Development and Evaluation

IAP = idiopathic acute pancreatitis

IAP/APA = International Association of Pancreatology/American Pancreatic Association

IPMN = intraductal papillary mucinous neoplasm

IQR = interquartile range

MRCP = magnetic resonance cholangiopancreaticography

MRI = magnetic resonance imaging

Word count

Word count excluding title page, abstract, article summary (strengths and limitations), key words, tables, figure legend, author statement and references: 3546

For peer review only

For peer review only - http://bmjopen.bmj.com/site/about/guidelines.xhtml

Abstract

Introduction

Idiopathic acute pancreatitis (IAP) remains a dilemma for physicians as it is uncertain whether patients with IAP may actually have an occult etiology. It is unclear to what extent additional diagnostic modalities such as endoscopic ultrasonography (EUS) are warranted after a first episode of IAP in order to uncover this etiology. Failure to timely determine treatable etiologies delays appropriate treatment and might subsequently cause recurrence of acute pancreatitis. Therefore, the aim of the "Pancreatitis of Idiopathic origin: Clinical added value of endoscopic UltraSonography" (PICUS) study is to determine the value of routine EUS in determining the etiology of pancreatitis in patients with a first episode of IAP.

Methods and analysis

PICUS is designed as a multicenter prospective cohort study of 106 patients with a first episode of IAP after complete standard diagnostic work-up, in whom a diagnostic EUS will be performed. Standard diagnostic work-up will include a complete personal and family history, laboratory tests including serum alanine aminotransferase, calcium and triglyceride levels, and imaging by transabdominal ultrasound, magnetic resonance imaging or magnetic resonance cholangiopancreaticography after clinical recovery from the acute pancreatitis episode. The primary outcome measure is detection of etiology by EUS. Secondary outcome measures include pancreatitis recurrence rate, severity of recurrent pancreatitis, readmission, additional interventions, complications, length of hospital stay, quality of life, mortality and costs, during a follow-up period of 12 months.

For peer review only

Ethics and dissemination

PICUS is conducted according to the Declaration of Helsinki and Guideline for Good Clinical Practice. Five Medical Ethics Review Committees assessed PICUS. The results will be submitted for publication in an international peer-reviewed journal.

Trial registration

Netherlands Trial Register: NL7066, June 9th 2018. Prospectively registered.

For peer review only

Article summary: strengths and limitations

- This is the first prospective cohort study of only patients with a single episode of presumed IAP.
- This is the first prospective cohort study which only includes patients after complete standard diagnostic work-up (including exclusion based on blood serum ALT and imaging after clinical recovery).
- The multicenter nature of this study reduces the risk of patient selection bias.
- By following patients for a year after EUS, this study could establish the association between EUS, detection of etiology and subsequent treatment of etiology, and pancreatitis recurrence.
- As the timing of the EUS is set to be after clinical recovery from pancreatitis in this trial, no conclusions on the diagnostic yield of EUS in a different time frame can be drawn from this study.

For peer review only - http://bmjopen.bmj.com/site/about/guidelines.xhtml

Keywords

Idiopathic acute pancreatitis; endoscopic ultrasonography, etiology

Background

Acute pancreatitis can be induced by numerous causes. Gallstone disease (approximately 50%) and alcohol (approximately 20%) are the most frequent causes (1-6), although the prevalence of etiologies of acute pancreatitis is dependent on, among other things, age and geographical factors (7-10). There is, however, a considerable group of patients of approximately 25% in whom no etiology can be found after routine diagnostic work-up (i.e. medical history, laboratory investigations and transabdominal ultrasound). These patients are considered to have presumed idiopathic acute pancreatitis (IAP) (3).

When IAP is presumed, guidelines recommend repeat transabdominal ultrasound after discharge (11, 12). This repeat ultrasonography has an additional diagnostic yield of 20% for the detection of gallstones or sludge in these patients (13). Undetected microlithiasis and biliary sludge are generally considered to be the major cause of presumed IAP (14, 15). Undetected and subsequently untreated gallstone disease poses a risk for recurrent acute pancreatitis and other biliary events, e.g. cholecystitis, biliary colic's and cholangitis.

Therefore, when previous diagnostics failed to uncover an etiology, endoscopic ultrasonography (EUS) should be considered for the detection of biliary disease or other abnormalities causing pancreatitis, such as neoplasms and chronic pancreatitis (11, 12, 16, 17). EUS is advised as the first step in presumed IAP, followed by (secretin-enhanced) magnetic resonance cholangiopancreaticography (MRCP) to identify rare morphologic abnormalities (11), as EUS is considered to have a higher diagnostic yield than MRCP for clinically relevant causes (18).

For peer review only

Although guidelines do recommend performing EUS after a first or second attack of presumed IAP, this recommendation is scored as a mere grade 2C, according to the Grading of Recommendations Assessment, Development and Evaluation (GRADE) classification (19) (indicating a weak recommendation based on evidence of low quality, with weak agreement among experts in this field) (11). Therefore, EUS is not routinely performed as the exact significance in this patient group is unclear (11, 16).

The PICUS study was designed to determine whether routine EUS should be incorporated in the standard diagnostic work-up of a first episode of presumed IAP.

Methods and analysis

Study aim

The objective of this study is to determine the diagnostic yield of EUS for the detection of etiology in patients with a first episode of presumed IAP.

Depending on the diagnostic yield of EUS observed in the PICUS study, incorporation of EUS in routine diagnostic work-up of patients with a first episode of presumed IAP will be considered. A minimal diagnostic yield of 10% for any etiology will be regarded as reasonable to justify implementing routine EUS in the standard diagnostic work-up of a first episode of presumed IAP.

Study design and setting

PICUS is a multicenter prospective cohort study. A total of 106 patients will be included from 28 participating Dutch centers, including all 8 university centers and 20 large teaching hospitals. A listing

For peer review only

For peer review only - http://bmjopen.bmj.com/site/about/guidelines.xhtml

of the participating centers is included in the Authors' information. An overview of the study design, including screening procedures and follow-up, is provided in figure 1.

Study population

The subjects of this study have had a first episode of acute pancreatitis, as defined by the 2012 Revised Atlanta criteria (20), with an unknown origin after standard diagnostic work-up, according to the 2013 International Association of Pancreatology/American Pancreatic Association (IAP/APA) evidence-based guidelines on management of acute pancreatitis (11). The diagnostic modalities that constitute standard diagnostic work-up are listed in table 1 and additional file 1. The diagnostic tests as laid out in table 1 are to be performed in all subjects and these tests cannot show any signs of an etiology in all subjects. Potential etiologies and their definitions are listed in table 2 and additional file 1.

Eligibility criteria

The inclusion criteria are:

1. Patients of 18 years or older
2. First episode of presumed IAP after standard diagnostic work-up, as defined by the IAP/APA evidence-based guidelines on management of acute pancreatitis (11)
3. Informed consent for participation

The exclusion criteria are:

1. Known etiology
2. Chronic pancreatitis, as defined by the M-ANNHEIM criteria (21)
3. Recurrent pancreatitis

For peer review only - http://bmjopen.bmj.com/site/about/guidelines.xhtml

4. Altered anatomy which prohibits the endosonographist from visualizing the gall bladder, bile ducts, pancreas or pancreatic duct via EUS (e.g. gastric bypass surgery)
5. Diagnostic EUS aimed to determine etiology before inclusion

Endoscopic ultrasonography

EUS will be performed in routine clinical practice by an endosonographist. Use of linear or radial EUS will be at the discretion of the endosonographist. All Dutch endosonographists are trained to perform EUS according to the technique of Hawes and Fockens (22).

The endosonographist will systematically report, using a standardized Case Report Form (CRF), the experience of the endosonographist, visualization of anatomical structures (i.e. gall bladder, common bile duct and pancreatic duct), presence of local complications of acute pancreatitis, characteristics of biliary etiology (i.e. gallstones, microlithiasis and/or biliary sludge), characteristics of chronic pancreatitis, presence of (a) pancreatic or peri-ampullary benign or malignant tumor(s), characteristics of auto-immune pancreatitis, anatomic variations (e.g. pancreas divisum) or other anomalies (e.g. cholecystitis, vascular, renal, splenic or hepatic anomalies or ascites), and performance of fine needle aspiration or fine needle biopsy. Additionally, the type of endoscope, use of sedation, procedure related complications and results of the fine needle aspiration or biopsy will be systematically recorded by the study coordinator in a separate CRF.

Primary outcome measure

The primary outcome measure is the number and ratio of patients with presumed IAP in whom EUS detects a cause for the pancreatitis episode.

A positive EUS is defined as an EUS during which a definitive cause for the acute pancreatitis episode has been found; or during which abnormalities are visualized constituting a definitive cause,

For peer review only

For peer review only - http://bmjopen.bmj.com/site/about/guidelines.xhtml

after obtaining tissue and pathological examination. An overview of the exact findings scored as positive imaging is provided in table 3.

If during EUS pancreatic abnormalities are found, yet not enough to make a certain diagnosis of chronic pancreatitis according to the M-ANNHEIM classification (21), this imaging is considered to be negative, even though it did show abnormalities. This approach is chosen because the aim of this study is to determine the rate of which EUS can find a cause for the presumed IAP episode. For the same reason, report of an anatomical abnormality during EUS after a first episode of acute pancreatitis is not scored as positive imaging as pancreatic morphological changes are very common in IAP and not necessarily clinically relevant, as is elaborated on in the discussion (23).

Secondary outcome measures

The secondary outcome measures are recurrence rate of acute pancreatitis, severity of recurrent pancreatitis (20), readmission, performance of additional invasive procedures (e.g. cholecystectomy, endoscopic sphincterotomy), complications of EUS and of additional interventions, according to the Clavien-Dindo classification (24), length of hospital stay, quality of life, mortality and costs. Relevant definitions are reported in Additional File 2.

Sample size calculation

The sample size calculation was based on the primary outcome measure, diagnostic yield of EUS. Based on two previous studies reporting yield in patients with a first episode of presumed IAP (25, 26), adjusted for the PICUS study criteria for inclusion (i.e. requiring negative imaging after clinical recovery) and for positive imaging (i.e. excluding pancreas divisum as etiology), diagnostic yield was assumed to be 30%. Using a two-sided significance level (α) of 0.05, a power ($1 - \beta$) of 80%, 95 patients are needed to attain a 95% confidence interval (CI) with a range smaller than 10% above and below

For peer review only

the assumed yield of 30% (95% CI: 20.8, 39.2). Assuming a drop-out rate of 10%, a total of 106 patients will be included (27). The sample size was calculated using the software programs RStudio (28) and nQuery (29).

Follow-up

Data from patient records on primary and secondary outcome measures will be collected until 1 year after inclusion. Outpatient care and follow-up after the EUS is at the discretion of the treating physician, but an outpatient clinic visit after EUS to discuss the results of the EUS and potential subsequent appropriate treatment can be considered standard care.

In case of biliary disease, the patient will be considered for endoscopic retrograde cholangiopancreaticography (ERCP) with sphincterotomy when choledocho(-micro-)lithiasis or sludge in the common bile duct is present, and cholecystectomy, as is standard care for biliary pancreatitis. A (secretin-enhanced) MRCP will be recommended, if not performed earlier, if a patient is readmitted for a recurrent episode of acute pancreatitis after a negative EUS for etiology, in order to rule out structural anomalies such as pancreas divisum. This is in accordance with current guidelines (11).

Patients will be asked to fill out the Short Form-36 questionnaire in the validated Dutch translation on day 3 after inclusion, after 6 months and after 1 year. This questionnaire in both English and Dutch is included in additional file 3.

Statistical aspects

All included subjects will be evaluated for primary and secondary endpoints until 1 year after inclusion. The primary analysis will be based on intention-to-treat principles. For exploratory reasons a per-protocol analysis will be performed too.

For peer review only - http://bmjopen.bmj.com/site/about/guidelines.xhtml

The intention-to-treat population comprises all patients included in the study, regardless of adherence to study protocol. The per-protocol population is the subset of included patients who were treated with the guidelines of the protocol (i.e. meeting all eligibility criteria including all of the diagnostic tests required for the diagnosis of IAP, undergoing EUS as described in the "Endoscopic ultrasonography section"). A tabular listing of all patients excluded from the intention-to-treat population will be provided together with the reasons for exclusion.

All analyses will be performed in SPSS for Microsoft Windows. All data handling and analysis will be saved in a syntax-file. Results will be presented with all centers combined. A two-tailed p-value of < 0.05 is considered statistically significant.

For peer review only

Baseline variables

The reported baseline characteristics consist of age, sex, body mass index (BMI), previous cholecystectomy, nicotine and alcohol use, severity of pancreatitis, length of hospital stay, amylase, lipase, C-reactive protein, alanine transaminase, calcium, albumin and triglycerides levels in blood serum on admission, imaging modalities before EUS and their findings. Baseline characteristics of EUS will include timing of EUS, experience of endosonographist and type of sedation and type of endoscope used. Data will be presented in percentages or as mean with standard deviation, or in case of a skewed distribution as median with interquartile range (IQR).

Primary outcome measure: etiology detection rate

Overall detection rate of an etiology for the episode of acute pancreatitis will be presented as percentage with a 95% CI. Predefined subgroup analyses will be made for patients with and without obesity (cut-off at a BMI of 30), a previous cholecystectomy, alcohol use and local complications from the IAP episode. A subgroup analysis will also be made for patients with a transabdominal ultrasound

For peer review only - http://bmjopen.bmj.com/site/about/guidelines.xhtml

as imaging after clinical recovery and with magnetic resonance imaging (MRI) or MRCP as imaging after clinical recovery. Finally, a subgroup analysis will be made for EUS performed by endosonographists with and without extensive experience (cut-off at 400 endosonographies performed), use of linear or radial scope and type of sedation used. In subgroup analyses, the Chi-square test or the Fisher's exact test will be used, as appropriate, to compare etiology detection rate between subgroups. In subgroup analyses, comparability between groups regarding baseline variables will be checked. If the subgroups differ statistically significantly in one or more baseline variables, this will be corrected in a logistic regression analysis.

Secondary outcome measures

Secondary outcome measures will be described as percentages with 95% CI, as mean with standard deviation or median with IQR, as appropriate.

For recurrence rate, subgroup analyses will be made for patients with a positive and negative EUS, and in patients with a positive EUS, for patients who were and were not treated adequately. The same subgroup analyses as in the primary outcome measure, will also be applied on the recurrence rate. The Chi-square test or the Fisher's exact test will be used for comparison between subgroups, as appropriate.

For quality of life, subgroup analyses will be made for baseline versus follow-up quality of life and for patients with a positive and negative EUS, and with and without pancreatitis recurrence during follow-up. The (un-)paired T-test, Wilcoxon signed rank test or the Mann-Whitney U test will be used for comparisons between subgroups, as appropriate.

For peer review only

For peer review only - http://bmjopen.bmj.com/site/about/guidelines.xhtml

Cost analysis

The cost analysis will comprise direct medical costs, which are generated by healthcare utilization and include hospital admission periods and therapeutic and diagnostic procedures (30). Estimates of unit costs will be based on Dutch reference data from the cost guide of the Dutch Health Council (31). If this guide is an inappropriate determination of unit costs, the costs will be based on data provided by two hospital administrations (one university center and one general hospital) to account for the actual input of personnel, material and overhead over hospital resources used. Cost calculations will be used to determine cost of interventions (surgical, endoscopic or radiological) and diagnostic imaging. The cost analysis will be reported separately from the main study manuscript.

Patient and public involvement

The patient advocacy organization *'Alvleeskliervereniging Nederland'* was involved in the design of the PICUS study. The experience of the patient advocacy organization with IAP and participation in scientific research has driven the research question and design of the study with regards to patient burden. The patient advocacy organization will also be involved in the dissemination and implementation of the study results.

All patients eligible for participation will be asked to give written informed consent.

Ethics and dissemination

The PICUS study is conducted according to the principles of the Declaration of Helsinki (October 2013) and to the Guideline for Good Clinical Practice by the International Council for Harmonization (November 9 2016).

For peer review only - http://bmjopen.bmj.com/site/about/guidelines.xhtml

The need for ethical approval was waived by the Medical Ethics Review Committee of the Academic Medical Center on May 28, 2018 (W18_161 # 18.199), by the Medical Research Ethics Committee of the University Medical Center Utrecht on July 04, 2018 (18-469), by the Research Ethics Committee of Radboud university medical center on July 23, 2018 (2018-4520), by the Medical Ethics Review Committee of the Erasmus Medical Center on July 30, 2018 (MEC-2018-1293) and by the Medical Ethics Review Committee of the Maastricht University Medical Center on September 7, 2018 (2018-0685). Before start of inclusion, local board approval will be obtained in all participating centers.

The results of the PICUS study will be submitted for publication in an international peer-reviewed scientific journal, regardless of study outcomes.

Discussion

Previous research has suggested that EUS might be beneficial in the detection of an etiology in presumed IAP. However, data lacks on the efficacy of routine EUS in patients with a first episode of presumed IAP, after repeat imaging after clinical recovery is negative for an etiology. The PICUS study aims to determine whether routine EUS is warranted in a first episode of acute pancreatitis where no cause could be uncovered after complete standard diagnostic work-up.

Currently, guidelines do not clearly define criteria for biliary origin (11). However, it is generally agreed upon that cholelithiasis, microlithiasis or biliary sludge constitute biliary etiology. Several previous studies have shown an association between elevated ALT levels and acute biliary pancreatitis (32-35), with a positive predictive value of 85% for an ALT > 150 U/L within 48 hours after onset of symptoms (11, 32, 33, 35). Therefore, an elevated blood serum ALT level at admission is considered to entail a high probability of biliary etiology, and pancreatitis with an elevated ALT is treated as being of biliary origin (32-34, 36). However, the majority of current literature on EUS did not exclude patients

For peer review only - http://bmjopen.bmj.com/site/about/guidelines.xhtml

based on ALT level at admission (15, 25, 26, 32, 37-46). As these patients have a higher a priori chance of confirmation of biliary etiology on EUS, the etiology detection rate of EUS might be overestimated in these studies. In PICUS, biliary etiology is defined as either the signs of cholelithiasis, microlithiasis or biliary sludge on transabdominal ultrasonography, or transient elevation of the blood serum ALT level of more than twice the upper limit of normal at admission in the absence of ALT elevating comorbidity. By only including patients with normal or slightly elevated ALT levels at admission, the etiology detection rate as reported in PICUS will reflect the detection rate in patients who are truly considered as having presumed IAP after standard diagnostic work-up.

Current guidelines advise a repeat transabdominal ultrasound after clinical recovery in the work-up of presumed IAP because the index transabdominal ultrasound is less sensitive during the acute phase of pancreatitis. The subpar visualization of gall bladder, bile ducts and pancreas is often due to excessive amounts of air in the intestines caused by pancreatitis-induced ileus and/or suboptimal cooperation of painful patients (47). After the first episode of acute pancreatitis, repeating a transabdominal ultrasound may be able to detect biliary stones where it could not during index admission (48). Of the current literature on EUS in IAP, however, only a minority of studies included repeat imaging in the diagnostic work-up before EUS (15, 40, 41, 43). Previous research has shown that a repeat transabdominal ultrasound has a diagnostic yield of 20% in patients with a first episode of IAP (49). Omitting repeat imaging from diagnostic work-up before EUS may lead to an overestimation of the diagnostic yield of EUS. In PICUS, all patients are required to undergo imaging after clinical recovery, i.e. transabdominal ultrasound or MRI/MRCP. Computed tomography (CT) is not considered sufficient imaging as biliary disease, the most common underlying etiology in presumed IAP, cannot always be adequately detected using CT.

It is well documented that the overall diagnostic yield of EUS in patients with recurrent pancreatitis is superior to the diagnostic yield of both secretin-enhanced MRCP (s-MRCP) and non-secretin-enhanced MRCP (18, 44, 46, 50). In the subgroup of patients with a pancreas divisum,

For peer review only

For peer review only - http://bmjopen.bmj.com/site/about/guidelines.xhtml

however, s-MRCP is considered to be superior in diagnostic yield to both EUS and MRCP (18). The role of pancreas divisum in the etiology of pancreatitis is unclear. Epidemiological studies have shown that the prevalence of pancreas divisum in the general population is equal to the prevalence in patients with presumed IAP (23). In patients with a pancreas divisum and acute pancreatitis, potentially other disease modifying factors add to the occurrence of pancreatitis, such as increased sensitivity to toxins or genetic susceptibility. Because of this ambiguity, pancreas divisum in patients with a first episode of acute pancreatitis is mostly left untreated in clinical practice. However, if patients with a pancreas divisum present with multiple episodes of presumed IAP, the divisum is often considered to be related to the pancreatitis and is subsequently treated, often with ERCP with endoscopic sphincterotomy, although evidence supporting this practice is limited (23). Because of both the diagnostic superiority of EUS in recurrent pancreatitis as well as the lack of clinical consequences of (s-)MRCP in patients with a first episode of pancreatitis, EUS is preferred to (s-)MRCP as the first choice for additional diagnostic testing for etiology in patients with presumed IAP (18, 44, 46, 50). Subsequently, current guidelines advise performing MRCP in case of recurrent IAP after EUS fails to determine an etiology (11). Therefore, in PICUS, we have chosen not to systematically include (s-)MRCP in the diagnostic work-up before EUS of first episode IAP.

Current guidelines advise consideration of EUS after a first or second attack of IAP (11). However, there is a paucity of evidence on the efficacy of EUS in first episode IAP. Three previous studies prospectively reported on EUS in patients with first episode IAP (25, 26, 38). However, in these studies, patients were not excluded based on liver enzymes abnormalities suggestive of biliary disease and no repeat imaging after clinical recovery was performed. PICUS will be the first prospective cohort study in which EUS will be performed in patients with a first episode of IAP after complete standard diagnostic work-up before EUS according to current guidelines (11).

A diagnostic yield of 10% for any etiology will be considered reasonable to justify incorporating routine EUS after a first episode of presumed IAP. This cut-off value was determined during a

For peer review only

For peer review only - http://bmjopen.bmj.com/site/about/guidelines.xhtml

multidisciplinary meeting of the Dutch Pancreatitis Study Group, which included the principal investigators of several trials being executed by the Dutch Pancreatitis Study Group. Considering the expectation that the majority of uncovered etiologies by EUS will be treatable (e.g. biliary disease) and adequate treatment could prevent pancreatitis recurrence, while in a minority of uncovered etiologies diagnosis before progression of disease might be crucial for prognosis (e.g. malignancy), a positive result in 10% of patients was deemed sufficient to warrant routine EUS after a first episode of presumed IAP.

In conclusion, the PICUS study is the first prospective cohort study of patients with a single episode of presumed IAP after complete standard diagnostic work-up (including exclusion based on blood serum ALT and imaging after clinical recovery). The results of the PICUS study will establish whether routine EUS should be incorporated in the guidelines for standard diagnostic work-up after a first episode of presumed IAP.

For peer review only

Acknowledgements

The authors would like to acknowledge:

1. all of the members of the Dutch Pancreatitis Study Group, for their continuous efforts in recruitment of patients,
2. dr. S. van Dieren, for providing her expertise in the calculation of the sample size,
3. dr. M. van der Vlugt, MD, PhD, for her willingness to act as independent physician for the PICUS study, and
4. the pancreatic disease patient advocacy organization *Alvleesklierverenigin Nederland*, for their effort in representing the perspective of (participating) patients during the study design.

For peer review only - http://bmjopen.bmj.com/site/about/guidelines.xhtml

References

1. Nesvaderani M, Eslick GD, Vagg D, Faraj S, Cox MR. Epidemiology, aetiology and outcomes of acute pancreatitis: A retrospective cohort study. Int J Surg. 2015;23(Pt A):68-74.

2. van Brunschot S, van Grinsven J, van Santvoort HC, Bakker OJ, Besselink MG, Boermeester MA, et al. Endoscopic or surgical step-up approach for infected necrotising pancreatitis: a multicentre randomised trial. The Lancet. 2018;391(10115):51-8.

3. Bakker OJ, van Brunschot S, van Santvoort HC, Besselink MG, Bollen TL, Boermeester MA, et al. Early versus on-demand nasoenteric tube feeding in acute pancreatitis. N Engl J Med. 2014;371(21):1983-93.

4. Besselink MGH, van Santvoort HC, Buskens E, Boermeester MA, van Goor H, Timmerman HM, et al. Probiotic prophylaxis in predicted severe acute pancreatitis: a randomised, double-blind, placebo-controlled trial. The Lancet. 2008;371(9613):651-9.

5. Bakker OJ, van Santvoort HC, van Brunschot S, Geskus RB, Besselink MG, Bollen TL, et al. Endoscopic Transgastric vs Surgical Necrosectomy for Infected Necrotizing Pancreatitis A Randomized Trial. JAMA. 2012;307(10):1053-61.

6. Van Santvoort HC, Besselink MG, Bakker OJ, Hofker HS, Boermeester MA, dejong CH, et al. A Step-up Approach or Open Necrosectomy for Necrotizing Pancreatitis. N Engl J Med. 2010;362:1491-502.

7. Sadr-Azodi O, Oskarsson V, Discacciati A, Videhult P, Askling J, Ekbom A. Pancreatic Cancer Following Acute Pancreatitis: A Population-based Matched Cohort Study. Am J Gastroenterol. 2018;113(11):1711-9.

8. Zheng Y, Zhou Z, Li H, Li J, Li A, Ma B, et al. A Multicenter Study on Etiology of Acute Pancreatitis in Beijing During 5 Years. Pancreas. 2015;44(3):409-14.

9. Zhu Y, Pan X, Zeng H, He W, Xia L, Liu P, et al. A Study on the Etiology, Severity, and Mortality of 3260 Patients With Acute Pancreatitis According to the Revised Atlanta Classification in Jiangxi, China Over an 8-Year Period. Pancreas. 2017;46(4):504-9.

For peer review only

10. Roberts SE, Morrison-Rees S, John A, Williams JG, Brown TH, Samuel DG. The incidence and aetiology of acute pancreatitis across Europe. Pancreatology. 2017;17(2):155-65.

11. Working Group IAP/APA Acute Pancreatitis Guidelines. IAP/APA evidence-based guidelines for the management of acute pancreatitis. Pancreatology. 2013;13(4 Suppl 2):e1-15.

12. UK Working Party on Acute Pancreatitis. UK guidelines for the management of acute pancreatitis. Gut. 2005;54 Suppl 3:iii1-9.

13. Hallensleben ND, Umans DS, Bouwense SA, Verdonk RC, Romkens TE, Witteman BJ, et al. The diagnostic work-up and outcomes of 'presumed' idiopathic acute pancreatitis: A post-hoc analysis of a multicentre observational cohort. United European Gastroenterol J. in press.

14. Lee SP, Nicholls JF, Park HZ. Biliary Sludge as a Cause of Acute Pancreatitis. N Engl J Med. 1992;326:589-93.

15. Garg PK, Tandon RK, Madan K. Is biliary microlithiasis a significant cause of idiopathic recurrent acute pancreatitis? A long-term follow-up study. Clin Gastroenterol Hepatol. 2007;5(1):75-9.

16. Tenner S, Baillie J, DeWitt J, Vege SS, American College of G. American College of Gastroenterology guideline: management of acute pancreatitis. Am J Gastroenterol. 2013;108(9):1400-15; 16.

17. Banks PA, Freeman ML, Practice Parameters Committee of the American College of G. Practice guidelines in acute pancreatitis. Am J Gastroenterol. 2006;101(10):2379-400.

18. Wan J, Ouyang Y, Yu C, Yang X, Xia L, Lu N. Comparison of EUS with MRCP in idiopathic acute pancreatitis: a systematic review and meta-analysis. Gastrointest Endosc. 2018.

19. Schünemann H BJ, Guyatt G, Oxman A, editors. GRADE handbook for grading quality of evidence and strength of recommendations. Group TGW, editor2013.

20. Banks PA, Bollen TL, Dervenis C, Gooszen HG, Johnson CD, Sarr MG, et al. Classification of acute pancreatitis--2012: revision of the Atlanta classification and definitions by international consensus. Gut. 2013;62(1):102-11.

For peer review only

For peer review only - http://bmjopen.bmj.com/site/about/guidelines.xhtml

21. Schneider A, Lohr JM, Singer MV. The M-ANNHEIM classification of chronic pancreatitis: introduction of a unifying classification system based on a review of previous classifications of the disease. J Gastroenterol. 2007;42(2):101-19.

22. Hawes RH, Fockens P. Endosonography. Second edition ed. Philadelphia: Elsevier Inc.; 2011.

23. DiMagno MJ, Wamsteker EJ. Pancreas divisum. Curr Gastroenterol Rep. 2011;13(2):150-6.

24. Dindo D, Demartines N, Clavien P-A. Classification of Surgical Complications. Annals of Surgery. 2004;240(2):205-13.

25. Yusoff IF, Raymond G, Sahai AV. A prospective comparison of the yield of EUS in primary vs. recurrent idiopathic acute pancreatitis. Gastrointest Endosc. 2004;60(5):673-8.

26. Vila JJ, Vicuna M, Irisarri R, de la Higuera BG, Ruiz-Clavijo D, Rodriguez-Gutierrez C, et al. Diagnostic yield and reliability of endoscopic ultrasonography in patients with idiopathic acute pancreatitis. Scand J Gastroenterol. 2010;45(3):375-81.

27. Whitley E, Ball J. Statistics review 4: Sample size calculations. Critical Care 2002;6(4):335-41.

28. R-Core-Team. R: A Language and Environment for Statistical Computing. Vienna, Austria: The R Foundation for Statistical Computing; 2017.

29. Oliver MR, Yeghiazarian K, Zheng M, Jamshidian M, Koyfman I. nQuery Advisor. 1995-2007.

30. Gold MR, Siegel JE, Russel LB. Cost-effectiveness in Health and Medicine. New York: Oxford University Press; 1996.

31. Zorginstituut Nederland (Health Institute Netherlands). Kostenhandleiding: Methodologie van kostenonderzoek en referentieprijzen voor economische evaluaties in de gezondheidszorg (Cost Guide: Methology of cost analysis and reference prices for economical evaluations in health care) 2015.

32. Liu CL, Fan ST, Lo CM, Tso WK, Wong Y, Poon RT, et al. Clinico-biochemical prediction of biliary cause of acute pancreatitis in the era of endoscopic ultrasonography. Aliment Pharmacol Ther. 2005;22(5):423-31.

For peer review only

For peer review only - http://bmjopen.bmj.com/site/about/guidelines.xhtml

33. Moolla Z, Anderson F, Thomson SR. Use of amylase and alanine transaminase to predict acute gallstone pancreatitis in a population with high HIV prevalence. World J Surg. 2013;37(1):156-61.

34. Ammori BJ, Boreham B, Lewis P, Roberts SA. The Biochemical Detection of Biliary Etiology of Acute Pancreatitis on Admission: A Revisit in the Modern Era of Biliary Imaging. Pancreas. 2003;26:e32-5.

35. Tenner SD, H.; Steinberg, W. Predicting gallstone pancreatitis with laboratory parameters: a meta-analysis. Am J Gastroenterol. 1994;89:1863-6.

36. Schepers NJ, Bakker OJ, Besselink MG, Bollen TL, Dijkgraaf MG, van Eijck CH, et al. Early biliary decompression versus conservative treatment in acute biliary pancreatitis (APEC trial): study protocol for a randomized controlled trial. Trials. 2016;17:5.

37. Frossard JL, Sosa-Valencia L, Amouyal G, Marty O, Hadengue A, Amouyal P. Usefulness of Endoscopic Ultrasonography in Patients with "Idiopathic" Acute Pancreatitis. Am J Med. 2000;109:196-200.

38. Govil A, Agrawal MK, Agrawal D, Udawat H. Role of endoscopic ultrasonography in patients with first episode of idiopathic acute pancreatitis. Indian J Gastroenterol. 2014;33(3):241-8.

39. Mariani A, Arcidiacono PG, Curioni S, Giussani A, Testoni PA. Diagnostic yield of ERCP and secretin-enhanced MRCP and EUS in patients with acute recurrent pancreatitis of unknown aetiology. Dig Liver Dis. 2009;41(10):753-8.

40. Morris-Stiff G, Al-Allak A, Frost B, Lewis WG, Puntis MC, Roberts A. Does Endoscopic Ultrasound Have Anything to Offer in the Diagnosis of Idiopathic Acute Pancreatitis? JOP. 2009;10(2):143-6.

41. Norton SA, Alderson D. Endoscopic ultrasonography in the evaluation of idiopathic acute pancreatitis British Journal of Surgery. 2000;97:1650-5.

42. Queneau P-E, Zeeh S, Lapeyre V, Thibault P, Heyd B, Carayon P, et al. Feasibility of and Interest in Combined Endoscopic Ultrasonography and Biliary Drainage in Unexplained Acute Biliopancreatic Disorders. Dig Dis Sci. 2002;47(9):2020-4.

For peer review only

43. Rana SS, Bhasin DK, Rao C, Singh K. Role of endoscopic ultrasound in idiopathic acute pancreatitis with negative ultrasound, computed tomography, and magnetic resonance cholangiopancreatography. Ann Gastroenterol. 2012;25(2):133-7.

44. Repiso Ortega A, Gómez-Rodríguez R, Romero M, Fernández-Zapardiel S, del Mar Céspedes M, Carrobles J-M. Prospective Comparison of Endoscopic Ultrasonography and Magnetic Resonance Cholangiopancreatography in the Etiological Diagnosis of ‘‘Idiopathic’’ Acute Pancreatitis. Pancreas. 2011;40:289-94.

45. Tandon M, Topazian M. Endoscopic Ultrasound in Idiopathic Acute Pancreatitis. Am J Gastroenterol. 2001;96(3):705–9.

46. Thevenot A, Bournet B, Otal P, Canevet G, Moreau J, Buscail L. Endoscopic ultrasound and magnetic resonance cholangiopancreatography in patients with idiopathic acute pancreatitis. Dig Dis Sci. 2013;58(8):2361-8.

47. Lara LF, Levy MJ. Idiopathic Recurrent Acute Pancreatitis. MedGenMed. 2004;6(4):10.

48. Signoretti M, Baccini F, Piciucchi M, Iannicelli E, Valente R, Zerboni G, et al. Repeated Transabdominal Ultrasonography Is a Simple and Accurate Strategy to Diagnose a Biliary Etiology of Acute Pancreatitis. Pancreas. 2014;43(7):1106-10.

49. Hallensleben ND, Umans DS, Bouwense S, Verdonk RC, Besselink MG, Van Hooft JE, et al. The clinical course and diagnostic work-up of idiopathic acute pancreatitis, a post-hoc analysis of a prospective multicenter observational cohort. Digestive Disease Week; San Diego: Gastroenterology; 2019. p. S-121.

50. Meeralam Y, Al-Shammari K, Yaghoobi M. Diagnostic accuracy of EUS compared with MRCP in detecting choledocholithiasis: a meta-analysis of diagnostic test accuracy in head-to-head studies. Gastrointest Endosc. 2017;86(6):986-93.

51. Stolle M, Sack PM, Thomasius R. Binge drinking in childhood and adolescence: epidemiology, consequences, and interventions. Dtsch Arztebl Int. 2009;106(19):323-8.

For peer review only

For peer review only - http://bmjopen.bmj.com/site/about/guidelines.xhtml

52. Sadr Azodi O, Orsini N, Andren-Sandberg A, Wolk A. Effect of type of alcoholic beverage in causing acute pancreatitis. Br J Surg. 2011;98(11):1609-16.

53. Midanik LT. Drunkenness, feeling the effects and 5+ measures. Addiction. 1999;94(6):887- 97.

54. Ratjen F, Döring G. Cystic fibrosis. The Lancet. 2003;361(9358):681-9.

55. Spanier B, Bruno MJ, Dijkgraaf MG. Incidence and mortality of acute and chronic pancreatitis in the Netherlands: a nationwide record-linked cohort study for the years 1995-2005. World J Gastroenterol. 2013;19(20):3018-26.

56. CBS StatLine. Households; size, position in household, January 1st 1995-2013 StatLine: Central Bureau for Statistics 2015 [Available from: http://statline.cbs.nl/Statweb/publication/?VW=T&DM=SLNL&PA=37312&D1=a&D2=0,5,10,(l-2)-l&HD=180221-1109&HDR=G1&STB=T.

57. Whitcomb DC. Genetic aspects of pancreatitis. Annu Rev Med. 2010;61:413-24.

58. Whitcomb DC. Hereditary diseases of the pancreas. In: Yamada T AD, Kaplowitz N, Laine L, Owyang C, Powell DW, editors, editor. Textbook of Gastroenterology. 4th ed. Philadelphia: Lippincott Williams & Wilkins; 2003. p. 2147–65.

59. Shane E, Dinaz I. Hypercalcemia: Pathogenesis, clinical manifestations, differential diagnosis and management. In: Favus M, editor. Primer on the metabolic bone diseases and disorders of mineral metabolism. 6th ed. Philadelpiha: Kippincott, Williams, and Wilkins; 2006. p. 26-176.

60. Berglund L, Brunzell JD, Goldberg AC, Goldberg IJ, Sacks F, Murad MH, et al. Evaluation and treatment of hypertriglyceridemia: an Endocrine Society clinical practice guideline. J Clin Endocrinol Metab. 2012;97(9):2969-89.

61. Nitsche C, Maertin S, Scheiber J, Ritter CA, Lerch MM, Mayerle J. Drug-induced pancreatitis. Curr Gastroenterol Rep. 2012;14(2):131-8.

62. Lankisch PG, Apte M, Banks PA. Acute pancreatitis. The Lancet. 2015;386(9988):85-96.

63. Tummala P, Tariq SH, Chibnall JT, Agarwal B. Clinical Predictors of Pancreatic Carcinoma Causing Acute Pancreatitis. Pancreas. 2013;42(1):108-13.

For peer review only

For peer review only - http://bmjopen.bmj.com/site/about/guidelines.xhtml

64. Cotton PB, Lehman G, Vennes J, Geenen JE, Russell RCG, Meyers WC, et al. Endoscopic sphincterotomy complications and their management: an attempt at consensus. Gastrointest Endosc. 1991;37:383-93.

65. Connor S. Defining post-operative pancreatitis as a new pancreatic specific complication following pancreatic resection. HPB (Oxford). 2016;18(8):642-51.

66. Booth F, Flint L. Pancreatoduodenal trauma. In: ed BJ, editor. Blunt multiple trauma. New York: Marcel Dekker; 1990. p. 497-509.

67. Smith I, Monkemuller K, Wilcox CM. Incidentally Identified Common Bile Duct Dilatation. A Systematic Review of Evaluation, Causes, and Outcome. J Clin Gastroenterol. 2015;49:810-5.

68. Mortelé KJ, Rocha TC, Streeter JL, Taylor AJ. Multimodality Imaging of Pancreatic and Biliary Congenital Anomalies. Radiographics. 2006;26(3):715-31.

Author Statement

Authors' contributions

DSU drafted the manuscript. HCT, RCV, SAB, MGB and JEvH co-authored the writing of the manuscript. DSU, RCV, SAB, MABo, MJB, PF, EJMvG, JWP, HCvS, FPV, MGB and JEvH were involved in the design of the study during several meetings of the Dutch Pancreatitis Study Group. NDHL, MPGFA, AB, RAB, MABr, LH, WLC, HMvD, BCvE, GWE, WLH, CVH, AI, LMK, SDK, LEP, RQ, TEHR, ACITLT, AYT, NGV, AMCJV, RLJvW and BJW critically assessed the study design, during several meetings, and edited the manuscript. All authors read and approved the final manuscript.

For peer review only

For peer review only - http://bmjopen.bmj.com/site/about/guidelines.xhtml

Funding statement

This work was supported by the Dutch Digestive Disease Foundation (*Maag Lever Darm Stichting*, grant number D17-25). The PICUS study is an investigator-initiated study. The sponsor had no influence on design, implementation and conduct of the study, as well as on collection, analysis and interpretation of data, construction of the manuscript and decision to publish.

Competing interests statement

The authors declare that they have no competing interests.

Data Availability Statement

The datasets used and/or analyzed during the current study are available from the corresponding author on reasonable request.

Figure legend

Figure 1: Overview of screening and study procedures. MRI = magnetic resonance imaging. MRCP = magnetic resonance cholangiopancreaticography. CRF = Case Report Form. EUS = endoscopic ultrasonography.

For peer review only

Table 1

**Standard diagnostic work-up**	
Detailed personal and family history, including questions on:	Alcohol use
	Recent ERCP
	Recent start or changes in use of drugs associated with acute pancreatitis
	Recent major abdominal trauma
	Recent abdominal surgery
	Familial and hereditary pancreatitis
	Cystic fibrosis-related pancreatitis
Laboratory tests, including:	Blood serum triglycerides level
	Blood serum calcium level, corrected for the blood serum albumin level
	Blood serum ALT level on admission
Imaging:	Transabdominal ultrasound, MRI or MRCP after clinical recovery

Table 1: Standard diagnostic work-up *Standard diagnostic work-up according to the 2013 IAP/APA evidence-based guideline on management of acute pancreatitis. A listing of the drugs considered to be associated with acute pancreatitis are listed in additional file 1. ERCP = endoscopic retrograde cholangiopancreaticography; ALT = alanine aminotransferase; MRI = magnetic resonance imaging; MRCP = magnetic resonance cholangiopancreaticography.*

For peer review only

For peer review only - http://bmjopen.bmj.com/site/about/guidelines.xhtml

645 Table 2

Etiology	Definition
Alcohol	> 4 units of alcohol in the 24 hours prior to start of abdominal complaints (51-53)
Biliary disease	1. A transient elevated ALT level of >2 times the upper limit of normal at diagnosis of acute pancreatitis, in the absence of other ALT elevating comorbidity (34), OR<br>2. Gallstones, microlithiasis and/or biliary sludge, OR<br>3. A dilated CBD of >8 mm in patients <76 years or >10 mm in patients >75 years at diagnosis of acute pancreatitis (36)
Cystic fibrosis	history of cystic fibrosis in the absence of another origin (54)
Familial	two or more direct blood-related family members (parents, children or siblings) who have had an episode of acute pancreatitis (55-57)
Hereditary	mutation in the PRSS1, SPINK1, CFTR, CTRC, CLDN2 or CPA1 gene, or direct family member (parents, children, siblings) with one or more of the above mentioned mutations and at least one direct family member who has (had) acute or chronic pancreatitis (57, 58)
Hypercalcemia	blood serum calcium level ≥12 mg/dl (3 mmol/l), corrected for serum albumin level, as first measured during admission (59)
Hypertriglyceridemia	blood serum triglyceride level of ≥1000 mg/dl (11.2 mmol/l) under fasting conditions, as first measured during admission (60)
Medication	use of drug(s) listed in additional file 1, which has or have been started or increased in dosage within a reasonable temporal sequence, in principle 1

For peer review only - http://bmjopen.bmj.com/site/about/guidelines.xhtml

	month before onset of pancreatitis, and has or have a positive dechallenge (a drug reaction that is confirmed by stopping the drug) (61, 62)
Neoplasm	Known hepatopancreatobiliary malignancy or known malignancy with metastases causing obstruction of the pancreatic duct (63)
ERCP	ERCP within 24 hours before diagnosis of pancreatitis (64)
Surgical	abdominal surgery within 24 hours prior to diagnosis of pancreatitis (65)
Trauma	typical blunt trauma to the upper abdomen and pancreatic trauma visible on imaging (66)

Table 2: potential etiologies and their definitions *Potential etiologies and their definitions. Side branch or mixed type intraductal papillary mucinous neoplasms without dilatation of the pancreatic duct and pancreas divisum will not be considered to be a causative factor for the pancreatitis episode. If imaging is not able to discriminate between gall bladder polyps or concrements, lesions smaller than 10 mm will not be considered an exclusion criterion. Lesions above 10 mm, irrespective of whether they are a polyp or a concrement, are an immediate indication for cholecystectomy, and these patients will be excluded from PICUS. ALT = alanine transaminase. CBD = common bile duct. ERCP = endoscopic retrograde cholangiopancreaticography.*

For peer review only

For peer review only - http://bmjopen.bmj.com/site/about/guidelines.xhtml

Table 3

Biliary pancreatitis	Presence of biliary stones, microlithiasis, or sludge
	Widened CBD, >8 mm in patients <76 years, or >10 mm in patients >75 years, in the absence of other CBD dilating factors (e.g. opioid use, distal stenosis, obstruction of external compression of CBD or papilla (67))
Chronic pancreatitis	Pancreatic calcifications
	> 4 of the following abnormal features of the pancreas:<br>1. Enlarged gland size<br>2. Cysts<br>3. Echo-poor lesions (focal areas of reduced echogenicity)<br>4. Echo-rich lesions (> 3 mm in diameter)<br>5. Accentuation of lobular pattern<br>6. Increased duct wall echogenicity<br>7. Irregularity of the main pancreatic duct<br>8. Dilation of the main pancreatic duct > 3.5 mm (68)<br>9. Visible side branches<br>10. Calcifications of the pancreatic duct
Neoplasms	Definitive diagnosis of pathological tissue after histological or cytological evaluation of specimen of an anomaly observed during EUS, e.g. hyperplastic or malignant tissue, or auto-immune inflammatory disease
	Main duct IPMN or mixed type IPMN causing dilatation of the pancreatic duct

Table 3: positive imaging *Definition of positive imaging. For each diagnosis, presence of one of the separately mentioned abnormalities is required to be considered as positive imaging. Specimen is not required to be obtained during EUS. Anatomical anomalies (e.g. divisum) are not considered a certain etiology in first episode IAP and therefore not considered as positive imaging. CBD = common bile duct. EUS = endoscopic ultrasonography. IPMN = intraductal papillary mucinous neoplasm.*

For peer review only

For peer review only - http://bmjopen.bmj.com/site/about/guidelines.xhtml

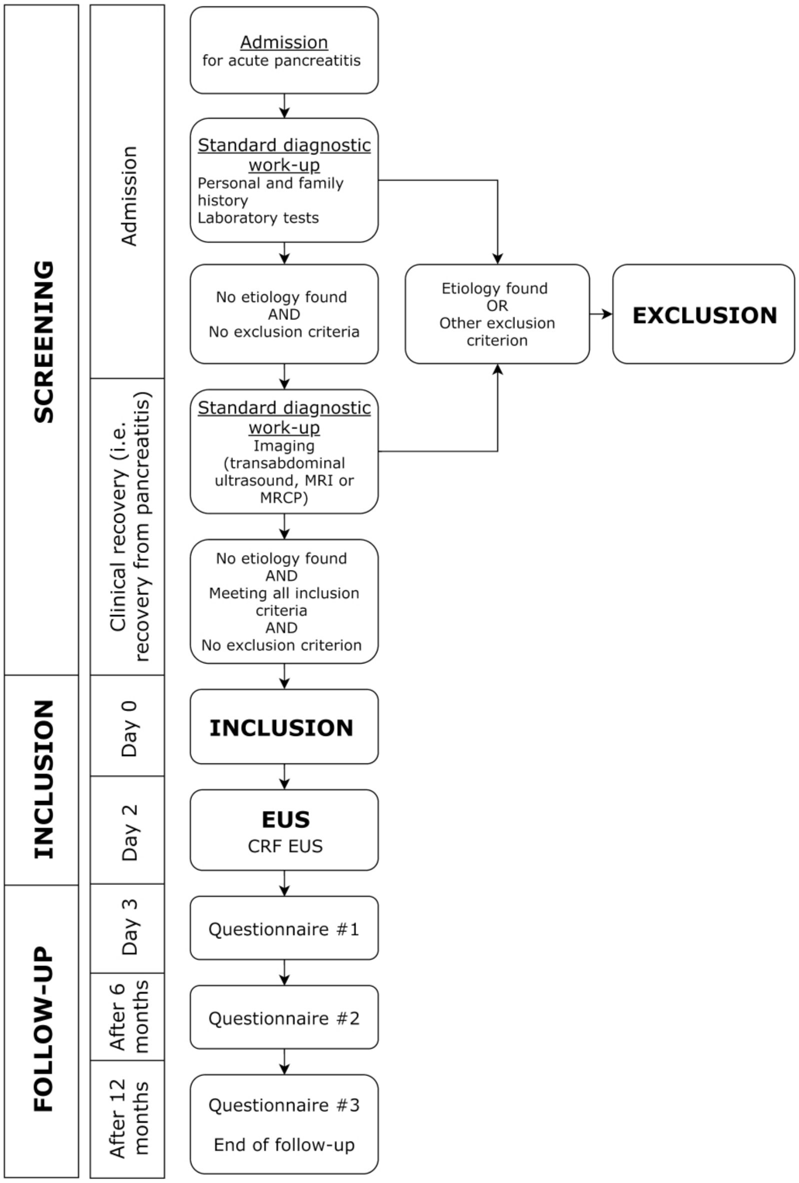

Overview of screening and study procedures. MRI = magnetic resonance imaging. MRCP = magnetic resonance cholangiopancreaticography. CRF = Case Report Form. EUS = endoscopic ultrasonography.

105x154mm (1200 x 1200 DPI)

Additional file 1: Table S1 Drugs associated with acute pancreatitis

**Drugs associated with acute pancreatitis**				
Acetaminophen	Cisplatin	Hydrochlorothiazide	Methyldopa	Pentavalent antimony compounds
Asparaginase	Cytarabine	Interferon alpha	Metronidazole	
Azathioprine	Didanosine	Itraconazole	Octreotide	Phenformin
Bortezomib	Enalapril	Lamivudine	Olanzapine	Simvastatin
Capecitabine	Erythromycin	Mercaptopurine	Opiates	Steroids
Carbamazepine	Estrogens	Mesalazine	Oxyphenbutazone	Sulfasalazine
Cimetidine	Furosemide	Olsalazine	Pentamidine	co-trimoxazole

Drugs with a definite association with acute pancreatitis (1, 2)

For peer review only

References

1. Nitsche C, Maertin S, Scheiber J, Ritter CA, Lerch MM, Mayerle J. Drug-induced pancreatitis. Curr Gastroenterol Rep. 2012;14(2):131-8.
2. Lankisch PG, Apte M, Banks PA. Acute pancreatitis. The Lancet. 2015;386(9988):85-96.

Additional file 2: Relevant definitions

Acute pancreatitis: an acute inflammation of the pancreatic parenchyma, diagnosed when at least two of the three following characteristics are present (1):

1. Clinical features of acute pancreatitis, such as upper abdominal pain
2. Elevated serum amylase or lipase levels of at least three times the upper limit of normal (ULN)
3. Signs of acute pancreatitis on imaging

Note: no value of the required serum amylase or lipase level is provided as every participating center has a local laboratory, which is why each center may use different normal range values.

Idiopathic acute pancreatitis is considered to be present if no etiology is found in standard work-up, according to the IAP/APA evidence-based guidelines on management of acute pancreatitis (2), which comprises at least the following tests:

1. A detailed personal and family history, including questions on:
    a. Alcohol use
    b. Recent endoscopic retrograde cholangiopancreaticography (ERCP)
    c. Recent start of or changes in use of drugs associated with acute pancreatitis
    d. Recent major abdominal trauma
    e. Recent abdominal surgery
    f. Familial pancreatitis
    g. Hereditary pancreatitis

For peer review only

h. Cystic fibrosis related pancreatitis

2. Laboratory tests, including:

a. Blood serum triglycerides level on admission

b. Blood serum calcium level, corrected for the serum albumin level, on admission

c. Blood serum alanine transaminase (ALT) level on admission

3. Imaging via transabdominal ultrasound, magnetic resonance imaging (MRI) or magnetic resonance cholangiopancreaticography (MRCP) after clinical recovery

Note: side branch or mixed type intraductal papillary mucinous neoplasms (IPMN) without dilatation of the pancreatic duct will not be considered to be a causative factor for the pancreatitis episode.

Note: if the imaging is not able to discriminate between gall bladder polyps or concrements, lesions smaller than 10 mm will not be considered an exclusion criterion. Lesions above 10 mm, irrespective of whether they are a polyp or a concrement, are an immediate indication for cholecystectomy, and will be excluded from PICUS.

Alcoholic pancreatitis: pancreatitis caused by an excess intake of alcohol, diagnosed when biliary etiology is not demonstrated by standard work-up and the patient has indicated (either by direct or indirect personal history or by findings during physical examination) to have drank at least five units of alcohol in the 24 hours prior to start of abdominal complaints (or in asymptomatic acute pancreatitis: prior to diagnosis) (3-5)

For peer review only

Biliary pancreatitis: pancreatitis caused by biliary stones, microlithiasis or sludge, diagnosed when one of the following features is present:

1. A transient elevated ALT level of more than two times the ULN at diagnosis of acute pancreatitis, in the absence of ALT elevating comorbidity (6)
2. Signs of presence of gallstones, microlithiasis or sludge on imaging, defined as follows:
    a. Gallstones, microlithiasis and/or biliary sludge, either in the gall bladder, ductus cysticus, intrahepatic bile ducts or in the common bile duct (CBD), and/or
    b. A CBD of more than eight mm in patients 75 years old or younger or more than ten mm in patients older than 75 years at diagnosis of acute pancreatitis (7)

Note: no value of the required serum ALT level is provided as the normal range values depend on the sex of the patient and as every participating center has a local laboratory, which is why each center may use different normal range values.

Chronic pancreatitis**:** a chronic inflammation of the pancreatic parenchyma, defined as typical clinical history of chronic pancreatitis (such as recurrent pancreatitis or abdominal pain, except for primary painless pancreatitis) and one or more of the following (8):

1. Pancreatic calcifications
2. Moderate or marked ductal lesions, defined as two or more of the following abnormal features on transabdominal ultrasound, computed tomography (CT) or MRI/MRCP, according to the Cambridge classification (9):
    a. Main pancreatic duct abnormalities, either enlargement or increased echogenicity of the duct wall (mandatory)
    b. Pancreatic enlargement

For peer review only

c. Cavities

d. Duct irregularities including intraductal fillings defects, calculi or duct obstruction

e. Focal acute pancreatitis

f. Parenchymal heterogeneity

g. Irregularities of pancreatic head or body contour

3. Moderate or marked ductal lesions, defined as five or more of the following abnormal features on endoscopic ultrasonography (EUS):

   a. Enlarged gland size

   b. Cysts

   c. Echo-poor lesions (focal areas of reduced echogenicity)

   d. Echo-rich lesions (more than three mm in diameter)

   e. Accentuation of lobular pattern (e.g., echo-poor normal parenchyma surrounded by hyperechoic strands)

   f. Increased duct wall echogenicity

   g. Irregularity of the main pancreatic duct (e.g., with narrowing of the duct)

   h. Dilation of the main pancreatic duct

   i. Visible side branches (e.g., with dilation)

   j. Calcification (of the pancreatic duct)

4. Marked and persistent exocrine insufficiency defined as pancreatic steatorrhea markedly reduced by enzyme supplementation

5. Typical histology of an adequate histological specimen

For peer review only

Note: during initial diagnostic work-up during admission 'marked and persistent exocrine insufficiency' cannot be evaluated properly. Therefore this part of the definition of chronic pancreatitis will not be applicable during standard work-up. However, if the patient does show marked and persistent exocrine insufficiency during follow-up (either during the outpatient clinic visit after repeat transabdominal ultrasound or after the EUS), this will be considered to be diagnostic for chronic pancreatitis. The same is applicable for histology of an adequate histological specimen: this is not part of standard work-up, however, if a typical histological specimen is obtained during follow-up, this will be considered to be diagnostic for chronic pancreatitis.

Clinical recovery from acute pancreatitis: resolution of pancreatic inflammation, present when one of the following criteria is met:

1. Discharge from the hospital
2. Normal inflammation parameters in laboratory tests
3. No signs of pancreatic inflammation on imaging

Cystic fibrosis: an autosomal recessive disorder caused by a mutation in the CFTR gene, resulting in defective chloride channels in epithelial cells, diagnosed by either a concentration in sweat of chloride greater than 60 mmol/L on repeated analysis, confirmation of a CFTR gene mutation, or both (10).

Cystic fibrosis related pancreatitis: pancreatitis caused by defective ductular and acinar pancreatic secretion, diagnosed when a patient with a history of cystic fibrosis presents with an acute pancreatitis in the absence of another origin (10).

For peer review only

Familial pancreatitis: acute pancreatitis from any cause that occurs in a family with an incidence that is greater than would be expected by chance alone, given the size of the family and the standardized incidence of pancreatitis within the Dutch population, defined as acute pancreatitis in patients who have two or more direct blood-related family members (parents, children or siblings) who have had an episode of acute pancreatitis (11-13).

Fever: a body temperature of 38.5˚C or higher.

Hereditary pancreatitis: otherwise unexplained pancreatitis in an individual from a family in which the pancreatitis phenotype appears to be inherited through a disease-causing gene mutation expressed in an autosomal dominant pattern, defined as pancreatitis in patients with a known mutation in the PRSS1 gene, the SPINK1 gene, the CFTR gene, the CTRC gene, the CLDN2 gene or the CPA1 gene, or if the patient has a direct family member (parents, children, siblings) with one or more of the above mentioned mutations and has at least one direct family member who has had an episode of acute pancreatitis or has chronic pancreatitis (13, 14).

Hypercalcemic pancreatitis: acute pancreatitis caused by hypercalcemia and diagnosed when no signs of a biliary pancreatitis are found in standard work-up and the patient has a blood serum calcium level of at least 12 mg/dl or 3 mmol/l, corrected for the serum albumin level, as first measured during admission (15).

For peer review only

Hypertriglyceridemic pancreatitis: acute pancreatitis based on hypertriglyceridemia and diagnosed if a biliary etiology is not demonstrated by standard work-up and the patient has a blood serum triglyceride level of at least 1000 mg/dl (or 11.2 mmol/l) under fasting conditions, as first measured during admission (16).

Hypothermia: a body temperature of 35.9˚C or lower.

Infected (extra)pancreatic necrosis: presence of microorganisms in (extra-)pancreatic necrosis, confirmed by a positive culture obtained by means of fine needle aspiration or from the first drainage procedure or necrosectomy, the presence of gas in the (extra-)pancreatic collection on CT, or the presence of clinical signs of persistent sepsis or progressive clinical deterioration despite maximal support on the intensive care unit (ICU) without other causes for infection (ruled out should be: pneumonia, urinary tract infection, wound infection, endocarditis, abdominal sepsis or any other infection which could be suspected based on the individual patient's clinical presentation) (17).

Medication associated pancreatitis: acute pancreatitis is considered to be caused by drugs when a biliary cause is not demonstrated by standard work-up, the patient uses one or multiple drug(s) listed in table S1 in additional file 1, the drug has been started or increased in dosage within a reasonable temporal sequence, in principle 1 month before the onset of the pancreatitis, and has a positive dechallenge (a drug reaction that is confirmed by stopping the drug) (18, 19).

Microlithiasis: stones or concrements, smaller than four mm, in the gall bladder or the bile ducts (20).

For peer review only

Murphy's sign: the phenomenon where compression of the right upper quadrant causes the patient to catch their breath due to pain when taking a deep breath (21).

Pancreas divisum: a congenital malformation of the main pancreatic duct (Wirsung's duct) with two separate ducts (a separate ventral duct of Wirsung and a dorsal duct of Santorini) as opposed to one main duct (of Wirsung) (22).

Positive imaging: positive imaging is defined as imaging during which a definitive cause for the acute pancreatitis episode can be found; or during which abnormalities are visualized constituting a definitive cause, after obtaining tissue and pathological examination. So, if during EUS ductal abnormalities are found, yet not enough to make a certain diagnosis of chronic pancreatitis according to the M-ANNHEIM classification (8), this imaging is considered to be negative, even though it did show abnormalities. This approach is chosen because the aim of this study is to determine the rate of which EUS can find a causative factor for a previous acute pancreatitis episode. For the same reason, finding of an anatomical abnormality after a first episode of acute pancreatitis is not scored as positive imaging. An overview of the exact findings scored as positive imaging is provided in table 3 of the main manuscript.

Post-ERCP pancreatitis: pancreatitis caused by mechanical injury from instrumentation and hydrostatic injury from contrast injection during ERCP, diagnosed if a patient develops a pancreatitis within 24 hours of an ERCP without indications of another origin (23).

For peer review only

For peer review only - http://bmjopen.bmj.com/site/about/guidelines.xhtml

Postoperative pancreatitis: pancreatitis caused by perioperative hypoperfusion of the pancreas, diagnosed if a patient develops a pancreatitis within 24 hours of abdominal surgery in the absence of indications for another origin (24).

Posttraumatic pancreatitis: pancreatitis caused by pancreatic injury due to trauma to the abdomen, diagnosed when the patient describes a typical blunt trauma to the upper abdomen and pancreatic trauma is visible on imaging (25).

Recurrence rate: the risk of a recurrent episode of acute pancreatitis.

Sludge: solid material which results from the slow settling of particles dispersed in bile (20).

For peer review only

Standard work-up:

1. A detailed personal and family history, including questions on:
    a. Alcohol use
    b. Recent ERCP
    c. Recent start of or changes in use of drugs associated with acute pancreatitis
    d. Recent major abdominal trauma
    e. Recent abdominal surgery
    f. Familial pancreatitis
    g. Hereditary pancreatitis
    h. Cystic fibrosis related pancreatitis
2. Laboratory tests, including:
    a. Blood serum triglycerides level, first measured during admission
    b. Blood serum calcium level, corrected for the serum albumin level, first measured during admission
    c. Blood serum ALT level on admission
3. Imaging via transabdominal ultrasound, MRI or MRCP after clinical recovery

Biliary events: acute cholecystitis; biliary colic's requiring readmission; biliary pancreatitis; cholangitis; or obstructive choledocholithiasis needing ERCP.

For peer review only

Acute cholecystitis: an acute inflammation of the gall bladder, diagnosed when one item in A, B and C is present:

A) Local signs of inflammation
   1. Murphy's' sign, or
   2. Right upper abdominal quadrant mass, pain or tenderness
B) Systemic signs of inflammation
   1. Fever or hypothermia, or
   2. Elevated C-reactive protein CRP), or
   3. Elevated white blood cell count
C) Imaging findings characteristic of acute cholecystitis (26, 27)

Note: acute cholecystitis and cholangitis (see definition below) are defined according to the Tokyo classification which defines fever as a body temperature of 38˚C or higher; however, fever will be defined in this study as hyperthermia of 38.5˚C or higher and hypothermia will be added as a systemic sign of inflammation, as this more accurately reflects clinical practice in the Netherlands.

Biliary colic**:** upper abdominal pain (either right upper quadrant or epigastric pain) lasting at least 30 minutes, often associated with restlessness (28).

For peer review only

Cholangitis: an inflammation of the bile duct(s), diagnosed when one item in each of the following categories is present:

1. Systemic inflammation
    a. Fever, hypothermia and/or shaking chills
    b. Laboratory data: evidence of inflammatory response (abnormal white blood cell counts (defined as smaller than 4,000/µl or larger than 10,000/µl), increase of serum CRP levels (defined as 1 mg/dl or higher), and other changes indicating inflammation)
2. Cholestasis
    a. Jaundice (defined as a total bilirubin of 2 mg/dl or higher)
    b. Laboratory data: abnormal liver function tests (increased serum alkaline phosphatase, gamma-glutamyltransferase (gamma-GT), aspartate transaminase (AST) and ALT levels (defined as more than 1.5 times the ULN))
3. Imaging
    a. Biliary dilatation
    b. Evidence of the etiology on imaging (stricture, stone, stent etc.) (26)

Obstructive choledocholithiasis: presence of gallstones, microlithiasis or biliary sludge in the CBD on imaging, requiring an ERCP, according to the treating physician.

For peer review only

References

1. Banks PA, Bollen TL, Dervenis C, Gooszen HG, Johnson CD, Sarr MG, et al. Classification of acute pancreatitis--2012: revision of the Atlanta classification and definitions by international consensus. Gut. 2013;62(1):102-11.

2. Working Group IAP/APA Acute Pancreatitis Guidelines. IAP/APA evidence-based guidelines for the management of acute pancreatitis. Pancreatology. 2013;13(4 Suppl 2):e1-15.

3. Stolle M, Sack PM, Thomasius R. Binge drinking in childhood and adolescence: epidemiology, consequences, and interventions. Dtsch Arztebl Int. 2009;106(19):323-8.

4. Sadr Azodi O, Orsini N, Andren-Sandberg A, Wolk A. Effect of type of alcoholic beverage in causing acute pancreatitis. Br J Surg. 2011;98(11):1609-16.

5. Midanik LT. Drunkenness, feeling the effects and 5+ measures. Addiction. 1999;94(6):887- 97.

6. Ammori BJ, Boreham B, Lewis P, Roberts SA. The Biochemical Detection of Biliary Etiology of Acute Pancreatitis on Admission: A Revisit in the Modern Era of Biliary Imaging. Pancreas. 2003;26:e32-5.

7. Schepers NJ, Bakker OJ, Besselink MG, Bollen TL, Dijkgraaf MG, van Eijck CH, et al. Early biliary decompression versus conservative treatment in acute biliary pancreatitis (APEC trial): study protocol for a randomized controlled trial. Trials. 2016;17:5.

8. Schneider A, Lohr JM, Singer MV. The M-ANNHEIM classification of chronic pancreatitis: introduction of a unifying classification system based on a review of previous classifications of the disease. J Gastroenterol. 2007;42(2):101-19.

9. Sarner M, Cotton PB. Classification of pancreatitis. Gut. 1984;25:756-9.

10. Ratjen F, Döring G. Cystic fibrosis. The Lancet. 2003;361(9358):681-9.

11. Spanier B, Bruno MJ, Dijkgraaf MG. Incidence and mortality of acute and chronic pancreatitis in the Netherlands: a nationwide record-linked cohort study for the years 1995-2005. World J Gastroenterol. 2013;19(20):3018-26.

12. CBS StatLine. Households; size, position in household, January 1st 1995-2013 StatLine: Central Bureau for Statistics 2015 [Available from: http://statline.cbs.nl/Statweb/publication/?VW=T&DM=SLNL&PA=37312&D1=a&D2=0,5,10,(l-2)-l&HD=180221-1109&HDR=G1&STB=T.

For peer review only

13. Whitcomb DC. Genetic aspects of pancreatitis. Annu Rev Med. 2010;61:413-24.

14. Whitcomb DC. Hereditary diseases of the pancreas. In: Yamada T AD, Kaplowitz N, Laine L, Owyang C, Powell DW, editors, editor. Textbook of Gastroenterology. 4th ed. Philadelphia: Lippincott Williams & Wilkins; 2003. p. 2147–65.

15. Shane E, Dinaz I. Hypercalcemia: Pathogenesis, clinical manifestations, differential diagnosis and management. In: Favus M, editor. Primer on the metabolic bone diseases and disorders of mineral metabolism. 6th ed. Philadelpiha: Kippincott, Williams, and Wilkins; 2006. p. 26-176.

16. Berglund L, Brunzell JD, Goldberg AC, Goldberg IJ, Sacks F, Murad MH, et al. Evaluation and treatment of hypertriglyceridemia: an Endocrine Society clinical practice guideline. J Clin Endocrinol Metab. 2012;97(9):2969-89.

17. van Brunschot S, van Grinsven J, van Santvoort HC, Bakker OJ, Besselink MG, Boermeester MA, et al. Endoscopic or surgical step-up approach for infected necrotising pancreatitis: a multicentre randomised trial. The Lancet. 2018;391(10115):51-8.

18. Nitsche C, Maertin S, Scheiber J, Ritter CA, Lerch MM, Mayerle J. Drug-induced pancreatitis. Curr Gastroenterol Rep. 2012;14(2):131-8.

19. Lankisch PG, Apte M, Banks PA. Acute pancreatitis. The Lancet. 2015;386(9988):85-96.

20. Jungst C, Kullak-Ublick GA, Jungst D. Gallstone disease: Microlithiasis and sludge. Best Pract Res Clin Gastroenterol. 2006;20(6):1053-62.

21. Murphy JB. The diagnosis of gall-stones. Am Med News. 1903;82:825.33.

22. Cotton P. Congenital anomaly of pancreas divisum as cause of obstructive pain and pancreatitis. Gut. 1980;21:105-14.

23. Cotton PB, Lehman G, Vennes J, Geenen JE, Russell RCG, Meyers WC, et al. Endoscopic sphincterotomy complications and their management: an attempt at consensus. Gastrointest Endosc. 1991;37:383-93.

24. Connor S. Defining post-operative pancreatitis as a new pancreatic specific complication following pancreatic resection. HPB (Oxford). 2016;18(8):642-51.

25. Booth F, Flint L. Pancreatoduodenal trauma. In: ed BJ, editor. Blunt multiple trauma. New York: Marcel Dekker; 1990. p. 497-509.

For peer review only

26. Miura F, Okamoto K, Takada T, Strasberg SM, Asbun HJ, Pitt HA, et al. Tokyo Guidelines 2018: initial management of acute biliary infection and flowchart for acute cholangitis. J Hepatobiliary Pancreat Sci. 2018;25(1):31-40.

27. Yokoe M, Takada T, Strasberg SM, Solomkin JS, Mayumi T, Gomi H, et al. TG13 diagnostic criteria and severity grading of acute cholecystitis (with videos). Journal of Hepato-Biliary-Pancreatic Sciences. 2013;20(1):35-46.

28. French E, Robb W. Biliary and renal colic. Br Med J. 1963:135-8.

For peer review only

Additional file 3: Short Form 36 Questionnaire

English version

1. In general, would you say your health is:
   ☐ excellent
   ☐ very good
   ☐ good
   ☐ fair
   ☐ poor

2. **Compared to one ear ago**, how would you rate your health in general **now**?
   ☐ much better now than one year ago
   ☐ somewhat better now than one year ago
   ☐ about the same
   ☐ somewhat worse now than one year ago
   ☐ much worse now than one year ago

For peer review only

3. The following items are about activities you might do during a typical day. Does **your health now limit you** in these activities? If so, how much?

	Yes, limited a lot	Yes, limited a little	No, not limited at all
a. **Vigorous activities**, such as running, lifting heavy objects, participating in strenuous sports	☐	☐	☐
b. **Moderate activities**, such as moving a table, pushing a vacuum cleaner, bowling, or playing golf	☐	☐	☐
c. Lifting or carrying groceries	☐	☐	☐
d. Climbing **several** flights of stairs	☐	☐	☐
e. Climbing **one** flight of stairs	☐	☐	☐
f. Bending, kneeling, or stooping	☐	☐	☐
g. Walking **more than a mile**	☐	☐	☐
h. Walking **several blocks**	☐	☐	☐
i. Walking **one block**	☐	☐	☐
j. Bathing or dressing yourself	☐	☐	☐

For peer review only

4. During the **past 4 weeks**, have you had any of the following problems with your work or other regular daily activities **as a result of your physical health**?

	Yes	No
a. Cut down the **amount of time** you spent on work or other activities	☐	☐
b. **Accomplished less** than you would like	☐	☐
c. Were limited in the **kind** of work or other activities	☐	☐
d. Had **difficulty** performing the work or other activities (for example, it took extra effort)	☐	☐

5. During the **past 4 weeks**, have you had any of the following problems with your work or other regular daily activities **as a result of any emotional problems** (such as feeling depressed or anxious)?

	Yes	No
a. Cut down the **amount of time** you spent on work or other activities	☐	☐
b. **Accomplished less** than you would like	☐	☐
c. Didn't do work or other activities as **carefully** as usual	☐	☐

For peer review only

6. During the **past 4 weeks**, to what extent has your physical health or emotional problems interfered with your normal social activities with family, friends, neighbors, or groups?

☐ not at all
☐ slightly
☐ moderately
☐ quite a bit
☐ extremely

7. How much **bodily** pain have you had during the **past 4 weeks**?

☐ none
☐ very mild
☐ mild
☐ moderate
☐ severe
☐ very severe

8. During the **past 4 weeks**, how much did **pain** interfere with your normal work (including both work outside the home and housework)?

☐ not at all
☐ a little bit
☐ moderately
☐ quite a bit
☐ extremely

For peer review only

9. These questions are about how you feel and how things have been with you **during the past 4 weeks**. For each question, please give the one answer that comes closest to the way you have been feeling.

How much of the time during the **past 4 weeks**…	**All of the time**	**Most of the time**	**A good bit of the time**	**Some of the time**	**A little of the time**	**None of the time**
a. Did you feel full of pep?	☐	☐	☐	☐	☐	☐
b. Have you been a very nervous person?	☐	☐	☐	☐	☐	☐
c. Have you felt so down in the dumps that nothing could cheer you up?	☐	☐	☐	☐	☐	☐
d. Have you felt calm and peaceful?	☐	☐	☐	☐	☐	☐
e. Did you have a lot of energy?	☐	☐	☐	☐	☐	☐
f. Have you felt downhearted and blue?	☐	☐	☐	☐	☐	☐
g. Did you feel worn out?	☐	☐	☐	☐	☐	☐
h. Have you been a happy person?	☐	☐	☐	☐	☐	☐
i. Did you feel tired?	☐	☐	☐	☐	☐	☐

For peer review only

10. During the **past 4 weeks**, how much of the time has **your physical health or emotional problems** interfered with your social activities (like visiting with friends, relatives, etc.)?

- ☐ all of the time
- ☐ most of the time
- ☐ some of the time
- ☐ a little of the time
- ☐ none of the time

11. How TRUE or FALSE is **each** of the following statements for you?

	**Definitely true**	**Mostly true**	**Don't know**	**Mostly false**	**Definitely false**
a. I seem to get sick a little easier than other people.	☐	☐	☐	☐	☐
b. I am as healthy as anybody I know.	☐	☐	☐	☐	☐
c. I expect my health to get worse.	☐	☐	☐	☐	☐
d. My health is excellent.	☐	☐	☐	☐	☐

Space for additional remarks with the questionnaire:

For peer review only

Dutch version

1. Wat vindt u, over het algemeen genomen, van uw gezondheid?
   - ☐ uitstekend
   - ☐ zeer goed
   - ☐ goed
   - ☐ matig
   - ☐ slecht

2. In vergelijking *met 1 jaar geleden*, hoe zou u *nu* uw gezondheid in het algemeen beoordelen?
   - ☐ veel beter dan een jaar geleden
   - ☐ iets beter dan een jaar geleden
   - ☐ ongeveer hetzelfde als een jaar geleden
   - ☐ iets slechter dan een jaar geleden
   - ☐ veel slechter dan een jaar geleden

For peer review only

3. De volgende vragen gaan over dagelijkse bezigheden. Wordt u door **uw gezondheid** *op dit moment* beperkt bij deze bezigheden? Zo ja, in welke mate?

	**Ja, ernstig**	**Ja, een beetje beperkt**	**Nee, helemaal niet beperkt**
a. *Forse inspanning* (zoals hardlopen, zware voorwerpen tillen, inspannend sporten)	☐	☐	☐
b. *Matige inspanning* (zoals het verplaatsen van een tafel, stofzuigen, fietsen)	☐	☐	☐
c. Tillen of boodschappen dragen	☐	☐	☐
d. *Een paar* trappen oplopen	☐	☐	☐
e. *Eén* trap oplopen	☐	☐	☐
f. Buigen, knielen of bukken	☐	☐	☐
g. *Meer dan een kilometer* lopen	☐	☐	☐
h. Een halve kilometer lopen	☐	☐	☐
i. *Honderd meter* lopen	☐	☐	☐
j. Uzelf wassen of aankleden	☐	☐	☐

For peer review only

4. Had u, ten gevolge van uw **lichamelijke gezondheid**, *de afgelopen 4 weken* één van de volgende problemen bij uw werk of andere bezigheden?

	Ja	Nee
a. U heeft *minder tijd* kunnen besteden aan werk of andere bezigheden.	☐	☐
b. U heeft *minder bereikt* dan u zou willen.	☐	☐
c. U was beperkt in het *soort* werk of soort bezigheden.	☐	☐
d. U had moeite met het werk of andere bezigheden (het kostte u bijvoorbeeld extra inspanning).	☐	☐

For peer review only

5. Had u, ten gevolge van een **emotioneel probleem** (bijvoorbeeld doordat u zich depressief of angstig voelde), *de afgelopen 4 weken* één van de volgende problemen bij uw werk of andere bezigheden?

	Ja	Nee
a. U heeft *minder tijd* kunnen besteden aan werk of andere bezigheden.	☐	☐
b. U heeft *minder bereikt* dan u zou willen.	☐	☐
c. U heeft het werk of andere bezigheden niet zo zorgvuldig gedaan als u gewend bent.	☐	☐

6. In hoeverre heeft uw **lichamelijke gezondheid** of hebben uw **emotionele problemen** u *de afgelopen 4 weken* belemmerd in uw normale sociale bezigheden met gezin, vrienden, buren of anderen?

☐ helemaal niet
☐ enigszins
☐ nogal
☐ veel
☐ heel erg veel

7. Hoeveel **pijn** had u *de afgelopen 4 weken?*

☐ geen
☐ heel licht
☐ licht
☐ nogal
☐ ernstig
☐ heel ernstig

8. In welke mate heeft **pijn** u *de afgelopen 4 weken* belemmerd bij uw normale werkzaamheden (zowel werk buitenshuis als huishoudelijk werk)?

☐ helemaal niet
☐ enigszins
☐ nogal
☐ veel
☐ heel erg veel

For peer review only

12. De volgende vragen gaan over hoe u zich *de afgelopen 4 weken* heeft **gevoeld**. Wilt u bij elke vraag het antwoord aankruisen dat het beste aansluit bij hoe u zich heeft gevoeld?

Hoe vaak gedurende *de afgelopen 4 weken:*	**Voortdurend**	**Meestal**	**Vaak**	**Soms**	**Zelden**	**Nooit**
a. voelde u zich levenslustig?	☐	☐	☐	☐	☐	☐
b. voelde u zich erg zenuwachtig?	☐	☐	☐	☐	☐	☐
c. zat u zo erg in de put dat niets u kon opvrolijken?	☐	☐	☐	☐	☐	☐
d. voelde u zich kalm en rustig?	☐	☐	☐	☐	☐	☐
e. voelde u zich erg energiek?	☐	☐	☐	☐	☐	☐
f. voelde u zich neerslachtig en somber?	☐	☐	☐	☐	☐	☐
g. voelde u zich uitgeblust?	☐	☐	☐	☐	☐	☐
h. voelde u zich gelukkig?	☐	☐	☐	☐	☐	☐
i. voelde u zich moe?	☐	☐	☐	☐	☐	☐

For peer review only

10. *Hoe vaak* hebben uw **lichamelijke gezondheid** of **emotionele problemen** gedurende *de afgelopen 4 weken* uw sociale activiteiten (zoals bezoek aan vrienden of naaste familieleden) belemmerd?

- ☐ voortdurend
- ☐ meestal
- ☐ soms
- ☐ zelden
- ☐ nooit

11. Wilt u het antwoord kiezen dat het beste weergeeft hoe juist of onjuist u elk van de volgende uitspraken voor uzelf vindt?

	Volkomen juist	Grotendeels juist	Weet ik niet	Grotendeels onjuist	Volkomen onjuist
a. Ik lijk gemakkelijker ziek te worden dan andere mensen.	☐	☐	☐	☐	☐
b. Ik ben net zo gezond als andere mensen die ik ken.	☐	☐	☐	☐	☐
c. Ik verwacht dat mijn gezondheid achteruit zal gaan.	☐	☐	☐	☐	☐
d. Mijn gezondheid is uitstekend.	☐	☐	☐	☐	☐

Ruimte voor aanvullende opmerkingen bij de vragenlijst:

For peer review only

BMJ Open

The role of endoscopic ultrasonography in the diagnostic work-up of idiopathic acute pancreatitis (PICUS): study protocol for a nationwide prospective cohort study

Journal:	*BMJ Open*
Manuscript ID	bmjopen-2019-035504.R2
Article Type:	Protocol
Date Submitted by the Author:	10-Jun-2020
Complete List of Authors:	Umans, Devica; Amsterdam University Medical Centres, Department of Gastroenterology and Hepatology; Saint Antonius Hospital, Research and Development<br>Timmerhuis, Hester; Saint Antonius Hospital, Department of Surgery; Saint Antonius Hospital, Research and Development<br>Hallensleben, Nora ; Erasmus Medical Center, Department of Gastroenterology and Hepatology; Saint Antonius Hospital, Research and Development<br>Bouwense, Stefan; Maastricht UMC+, Department of Surgery<br>Anten, Marie-Paule; Franciscus Gasthuis en Vlietland, Department of Gastroenterology and Hepatology<br>Bhalla, Abha; HagaZiekenhuis, Department of Gastroenterology and Hepatology<br>Bijlsma, Rina; Martini Ziekenhuis, Department of Gastroenterology and Hepatology<br>Boermeester, Marja; Amsterdam University Medical Centres, Department of Surgery<br>Brink, Menno; Meander MC, Department of Gastroenterology and Hepatology<br>Hol, Lieke; Maasstad Hospital, Department of Gastroenterology and Hepatology<br>Bruno, Marco; Erasmus Medical Center, Department of Gastroenterology and Hepatology<br>Curvers, Wouter; Catharina Hospital, Department of Gastroenterology and Hepatology<br>van Dullemen, Hendrik; UMCG, Department of Gastroenterology and Hepatology<br>van Eijck, Brechje; Spaarne Gasthuis, Department of Gastroenterology and Hepatology<br>Erkelens, G; Gelre Ziekenhuizen, Department of Gastroenterology and Hepatology<br>Fockens, Paul; Amsterdam University Medical Centres, Department of Gastroenterology and Hepatology<br>van Geenen, Erwin; Radboudumc<br>Hazen, Wouter; Elisabeth-TweeSteden Ziekenhuis, Department of Gastroenterology and Hepatology<br>Hoge, Chantal; Maastricht UMC+, Department of Gastroenterology and Hepatology<br>Inderson, Akin; LUMC, Department of Gastroenterology and Hepatology

	Kager, Liesbeth; Noordwest Ziekenhuisgroep, Department of Gastroenterology and Hepatology<br>Kuiken, Sjoerd; OLVG, Department of Gastroenterology and Hepatology<br>Perk, Lars; Medisch Centrum Haaglanden, Department of Gastroenterology and Hepatology<br>Poley, Jan-Werner<br>Quispel, Rutger; Reinier de Graaf Groep, Department of Gastroenterology and Hepatology<br>Römkens, Tessa; Jeroen Bosch Hospital, Department of Gastroenteroloy and Hepatology<br>van Santvoort, Hjalmar ; University Medical Center Utrecht; Saint Antonius Hospital, Department of Surgery<br>Tan, Adriaan; Canisius Wilhelmina Hospital, Department of Gastroenterology and Hepatology<br>Thijssen, Annemieke; Albert Schweitzer Ziekenhuis, Department of Gastroenterology and Hepatology<br>Venneman, Niels ; Medisch Spectrum Twente, Department of Gastroenterology and Hepatology<br>Vleggaar, Frank; Universitair Medisch Centrum Utrecht<br>Voorburg, Annet ; Diakonessenhuis Utrecht Zeist Doorn, Department of Gastroenterology and Hepatology<br>van Wanrooij, Roy; Amsterdam University Medical Centres, Department of Gastroenterology and Hepatology<br>Witteman, Ben; Ziekenhuis Gelderse Vallei, Department of Gastroenterology and Hepatology<br>Verdonk, Robert; Saint Antonius Hospital, Department of Gastroenterology and Hepatology<br>Besselink, Marc; Amsterdam University Medical Centres, Department of Surgery<br>van Hooft, Jeanin; AMC
**Primary Subject Heading**:	Gastroenterology and hepatology
Secondary Subject Heading:	Evidence based practice, Research methods
Keywords:	Endoscopy < GASTROENTEROLOGY, Hepatobiliary disease < GASTROENTEROLOGY, Pancreatic disease < GASTROENTEROLOGY

SCHOLARONE™ Manuscripts

I, the Submitting Author has the right to grant and does grant on behalf of all authors of the Work (as defined in the below author licence), an exclusive licence and/or a non-exclusive licence for contributions from authors who are: i) UK Crown employees; ii) where BMJ has agreed a CC-BY licence shall apply, and/or iii) in accordance with the terms applicable for US Federal Government officers or employees acting as part of their official duties; on a worldwide, perpetual, irrevocable, royalty-free basis to BMJ Publishing Group Ltd ("BMJ") its licensees and where the relevant Journal is co-owned by BMJ to the co-owners of the Journal, to publish the Work in this journal and any other BMJ products and to exploit all rights, as set out in our licence.

The Submitting Author accepts and understands that any supply made under these terms is made by BMJ to the Submitting Author unless you are acting as an employee on behalf of your employer or a postgraduate student of an affiliated institution which is paying any applicable article publishing charge ("APC") for Open Access articles. Where the Submitting Author wishes to make the Work available on an Open Access basis (and intends to pay the relevant APC), the terms of reuse of such Open Access shall be governed by a Creative Commons licence – details of these licences and which Creative Commons licence will apply to this Work are set out in our licence referred to above.

Other than as permitted in any relevant BMJ Author's Self Archiving Policies, I confirm this Work has not been accepted for publication elsewhere, is not being considered for publication elsewhere and does not duplicate material already published. I confirm all authors consent to publication of this Work and authorise the granting of this licence.

For peer review only

The role of endoscopic ultrasonography in the diagnostic work-up of idiopathic acute pancreatitis (PICUS): study protocol for a nationwide prospective cohort study

Devica S Umans[1, 2], Hester C Timmerhuis[2, 3], Nora DL Hallensleben[2, 4], Stefan A Bouwense[5], Marie-Paule GF Anten[6], Abha Bhalla[7], Rina A Bijlsma[8], Marja A Boermeester[9], Menno A Brink[10], Lieke Hol[11], Marco J Bruno[4], Wouter L Curvers[12], Hendrik M van Dullemen[13], Brechje C van Eijck[14], G Willemien Erkelens[15], Paul Fockens[1], Erwin JM van Geenen[16], Wouter L Hazen[17], Chantal V Hoge[18], Akin Inderson[19], Liesbeth M Kager[20], Sjoerd D Kuiken[21], Lars E Perk[22], Jan-Werner Poley[4], Rutger Quispel[23], Tessa EH Römkens[24], Hjalmar C van Santvoort[3, 25], Adriaan CITL Tan[26], Annemieke Y Thijssen[27], Niels G Venneman[28], Frank P Vleggaar[29], Annet MCJ Voorburg[30], Roy LJ van Wanrooij[31], Ben J Witteman[32], Robert C Verdonk[33], Marc G Besselink[8], Jeanin E van Hooft[1], for the Dutch Pancreatitis Study Group

[1] Department of Gastroenterology and Hepatology, Amsterdam Gastroenterology and Metabolism, Amsterdam UMC, University of Amsterdam, Amsterdam, the Netherlands

[2] Department of Research and Development, St. Antonius Hospital, Nieuwegein, the Netherlands

[3] Department of Surgery, St. Antonius Hospital, Nieuwegein, the Netherlands

[4] Department of Gastroenterology and Hepatology, Erasmus Medical Center, Rotterdam, the Netherlands

[5] Department of Surgery, Maastricht UMC+, Maastricht, the Netherlands

[6] Department of Gastroenterology and Hepatology, Franciscus Gasthuis & Vlietland, Rotterdam, the Netherlands

[7] Department of Gastroenterology and Hepatology, Haga Hospital, Den Haag, the Netherlands

[8] Department of Gastroenterology and Hepatology, Martini Hospital, Groningen, the Netherlands

[9] Department of Surgery, Amsterdam UMC, University of Amsterdam, Amsterdam, the Netherlands

For peer review only

[10] Department of Gastroenterology and Hepatology, Meander Medical Center, Amersfoort, the Netherlands

[11] Department of Gastroenterology and Hepatology, Maasstad Hospital, Rotterdam, the Netherlands

[12] Department of Gastroenterology and Hepatology, Catharina Hospital, Eindhoven, the Netherlands

[13] Department of Gastroenterology and Hepatology, University Medical Center Groningen, Groningen, the Netherlands

[14] Department of Gastroenterology and Hepatology, Spaarne Gasthuis, Haarlem, the Netherlands

[15] Department of Gastroenterology and Hepatology, Gelre Hospital, Apeldoorn, the Netherlands

[16] Department of Gastroenterology and Hepatology, Radboud university medical center, Nijmegen, the Netherlands

[17] Department of Gastroenterology and Hepatology, Elisabeth-Tweesteden Hospital, Tilburg, the Netherlands

[18] Department of Gastroenterology and Hepatology, Maastricht University Medical Center, Maastricht, the Netherlands

[19] Department of Gastroenterology and Hepatology, Leiden University Medical Center, Leiden, the Netherlands

[20] Department of Gastroenterology and Hepatology, Noordwest Ziekenhuisgroep, Alkmaar, the Netherlands

[21] Department of Gastroenterology and Hepatology, OLVG, Amsterdam, the Netherlands

[22] Department of Gastroenterology and Hepatology, Medical Center Haaglanden, Den Haag, the Netherlands

[23] Department of Gastroenterology and Hepatology, Reinier de Graaf Gasthuis, Delft, the Netherlands

[24] Department of Gastroenterology and Hepatology, Jeroen Bosch Hospital, Den Bosch, the Netherlands

[25] Department of Surgery, UMC Utrecht, Utrecht, the Netherlands

[26] Department of Gastroenterology and Hepatology, Canisius-Wilhelmina Hospital, Nijmegen, the Netherlands

[27] Department of Gastroenterology and Hepatology, Albert Schweitzer Hospital, Dordrecht, the Netherlands

[28] Department of Gastroenterology and Hepatology, Medisch Spectrum Twente, Enschede, the Netherlands

[29] Department of Gastroenterology and Hepatology, University Medical Center Utrecht, the Netherlands

[30] Department of Gastroenterology and Hepatology, Diakonessenhuis, Utrecht, the Netherlands

[31] Department of Gastroenterology and Hepatology, Amsterdam Gastroenterology and Metabolism, Amsterdam UMC, Vrije Universiteit van Amsterdam, Amsterdam, the Netherlands

[32] Department of Gastroenterology and Hepatology, Hospital Gelderse Vallei, Ede, the Netherlands

For peer review only

For peer review only - http://bmjopen.bmj.com/site/about/guidelines.xhtml

[33] Department of Gastroenterology and Hepatology, St. Antonius Hospital, Nieuwegein, the Netherlands

Correspondence

Dr. Jeanin E. van Hooft, MD, PhD, MBA

Amsterdam UMC, location AMC

Department of Gastroenterology and Hepatology, C2-115

PO Box 22660

1100 DD Amsterdam

The Netherlands

Telephone: +31 20 566 7918

E-mail: j.e.vanhooft@amsterdamumc.nl / j.e.van_hooft@lumc.nl

Correspondence during review phase

Devica S Umans

Telephone: +31 88 320 7051

E-mail: d.s.umans@amsterdamumc.nl

For peer review only

List of abbreviations

ALT = alanine aminotransferase

BMI = body mass index

CI = confidence interval

CRF = case report form

CT = computed tomography

ERCP = endoscopic retrograde cholangiopancreaticography

EUS = endoscopic ultrasonography

GRADE = Grading of Recommendations Assessment, Development and Evaluation

IAP = idiopathic acute pancreatitis

IAP/APA = International Association of Pancreatology/American Pancreatic Association

IPMN = intraductal papillary mucinous neoplasm

IQR = interquartile range

MRCP = magnetic resonance cholangiopancreaticography

MRI = magnetic resonance imaging

Word count

Word count excluding title page, abstract, article summary (strengths and limitations), key words, tables, figure legend, author statement and references: 3573

For peer review only

Abstract

Introduction

Idiopathic acute pancreatitis (IAP) remains a dilemma for physicians as it is uncertain whether patients with IAP may actually have an occult etiology. It is unclear to what extent additional diagnostic modalities such as endoscopic ultrasonography (EUS) are warranted after a first episode of IAP in order to uncover this etiology. Failure to timely determine treatable etiologies delays appropriate treatment and might subsequently cause recurrence of acute pancreatitis. Therefore, the aim of the "Pancreatitis of Idiopathic origin: Clinical added value of endoscopic UltraSonography" (PICUS) study is to determine the value of routine EUS in determining the etiology of pancreatitis in patients with a first episode of IAP.

Methods and analysis

PICUS is designed as a multicenter prospective cohort study of 106 patients with a first episode of IAP after complete standard diagnostic work-up, in whom a diagnostic EUS will be performed. Standard diagnostic work-up will include a complete personal and family history, laboratory tests including serum alanine aminotransferase, calcium and triglyceride levels, and imaging by transabdominal ultrasound, magnetic resonance imaging or magnetic resonance cholangiopancreaticography after clinical recovery from the acute pancreatitis episode. The primary outcome measure is detection of etiology by EUS. Secondary outcome measures include pancreatitis recurrence rate, severity of recurrent pancreatitis, readmission, additional interventions, complications, length of hospital stay, quality of life, mortality and costs, during a follow-up period of 12 months.

For peer review only

For peer review only - http://bmjopen.bmj.com/site/about/guidelines.xhtml

Ethics and dissemination

PICUS is conducted according to the Declaration of Helsinki and Guideline for Good Clinical Practice. Five Medical Ethics Review Committees assessed PICUS (Medical Ethics Review Committee of Academic Medical Center, University Medical Center Utrecht, Radboud university medical center, Erasmus Medical Center and Maastricht University Medical Center). The results will be submitted for publication in an international peer-reviewed journal.

Trial registration

Netherlands Trial Register: NL7066, June 9th 2018. Prospectively registered.

Article summary: strengths and limitations

- This is the first prospective cohort study of only patients with a single episode of presumed IAP.
- This is the first prospective cohort study which only includes patients after complete standard diagnostic work-up (including exclusion based on blood serum ALT and imaging after clinical recovery).
- The multicenter nature of this study reduces the risk of patient selection bias.
- This study has a follow-up time of a year, and thus this study could elucidate the previously hypothesized association between EUS, detection of etiology and subsequent treatment of etiology, and pancreatitis recurrence.
- As the timing of the EUS is set to be after clinical recovery from pancreatitis in this trial, no conclusions on the diagnostic yield of EUS in a different time frame can be drawn from this study.

For peer review only - http://bmjopen.bmj.com/site/about/guidelines.xhtml

Keywords

Idiopathic acute pancreatitis; endoscopic ultrasonography, etiology

Background

Acute pancreatitis can be induced by numerous causes. Gallstone disease (approximately 50%) and alcohol (approximately 20%) are the most frequent causes (1-6), although the prevalence of etiologies of acute pancreatitis is dependent on, among other things, age and geographical factors (7-10). There is, however, a considerable group of patients of approximately 25% in whom no etiology can be found after routine diagnostic work-up (i.e. medical history, laboratory investigations and transabdominal ultrasound). These patients are considered to have presumed idiopathic acute pancreatitis (IAP) (3).

When IAP is presumed, guidelines recommend repeat transabdominal ultrasound after discharge (11, 12). This repeat ultrasonography has an additional diagnostic yield of 20% for the detection of gallstones or sludge in these patients (13). Undetected microlithiasis and biliary sludge are generally considered to be the major cause of presumed IAP (14, 15). Undetected and subsequently untreated gallstone disease poses a risk for recurrent acute pancreatitis and other biliary events, e.g. cholecystitis, biliary colic's and cholangitis.

Therefore, when previous diagnostics failed to uncover an etiology, endoscopic ultrasonography (EUS) should be considered for the detection of biliary disease or other abnormalities causing pancreatitis, such as neoplasms and chronic pancreatitis (11, 12, 16, 17). EUS is advised as the first step in presumed IAP, followed by (secretin-enhanced) magnetic resonance cholangiopancreaticography (MRCP) to identify rare morphologic abnormalities (11), as EUS is considered to have a higher diagnostic yield than MRCP for clinically relevant causes (18).

For peer review only - http://bmjopen.bmj.com/site/about/guidelines.xhtml

Although guidelines do recommend performing EUS after a first or second attack of presumed IAP, this recommendation is scored as a mere grade 2C, according to the Grading of Recommendations Assessment, Development and Evaluation (GRADE) classification (19) (indicating a weak recommendation based on evidence of low quality, with weak agreement among experts in this field) (11). Therefore, EUS is not routinely performed as the exact significance in this patient group is unclear (11, 16).

The PICUS study was designed to determine whether routine EUS should be incorporated in the standard diagnostic work-up of a first episode of presumed IAP.

Methods and analysis

Study aim

The objective of this study is to determine the diagnostic yield of EUS for the detection of etiology in patients with a first episode of presumed IAP.

Depending on the diagnostic yield of EUS observed in the PICUS study, incorporation of EUS in routine diagnostic work-up of patients with a first episode of presumed IAP will be considered. A minimal diagnostic yield of 10% for any etiology will be regarded as reasonable to justify implementing routine EUS in the standard diagnostic work-up of a first episode of presumed IAP.

Study design and setting

PICUS is a multicenter prospective cohort study. A total of 106 patients will be included from 28 participating Dutch centers, including all 8 university centers and 20 large teaching hospitals. A listing

For peer review only

For peer review only - http://bmjopen.bmj.com/site/about/guidelines.xhtml

of the participating centers is included in the Authors' information. An overview of the study design, including screening procedures and follow-up, is provided in figure 1.

Study population

The subjects of this study have had a first episode of acute pancreatitis, as defined by the 2012 Revised Atlanta criteria (20), with an unknown origin after standard diagnostic work-up, according to the 2013 International Association of Pancreatology/American Pancreatic Association (IAP/APA) evidence-based guidelines on management of acute pancreatitis (11). The diagnostic modalities that constitute standard diagnostic work-up are listed in table 1 and additional file 1. The diagnostic tests as laid out in table 1 are to be performed in all subjects and these tests cannot show any signs of an etiology in all subjects. Potential etiologies and their definitions are listed in table 2 and additional file 1.

Eligibility criteria

The inclusion criteria are:

1. Patients of 18 years or older
2. First episode of presumed IAP after standard diagnostic work-up, as defined by the IAP/APA evidence-based guidelines on management of acute pancreatitis (11)
3. Informed consent for participation

The exclusion criteria are:

1. Known etiology
2. Chronic pancreatitis, as defined by the M-ANNHEIM criteria (21)
3. Recurrent pancreatitis

For peer review only - http://bmjopen.bmj.com/site/about/guidelines.xhtml

4. Altered anatomy which prohibits the endosonographist from visualizing the gall bladder, bile ducts, pancreas or pancreatic duct via EUS (e.g. gastric bypass surgery)
5. Diagnostic EUS aimed to determine etiology before inclusion

Endoscopic ultrasonography

EUS will be performed in routine clinical practice by an endosonographist. Use of linear or radial EUS will be at the discretion of the endosonographist. All Dutch endosonographists are trained to perform EUS according to the technique of Hawes and Fockens (22).

The endosonographist will systematically report, using a standardized Case Report Form (CRF), the experience of the endosonographist, visualization of anatomical structures (i.e. gall bladder, common bile duct and pancreatic duct), presence of local complications of acute pancreatitis, characteristics of biliary etiology (i.e. gallstones, microlithiasis and/or biliary sludge), characteristics of chronic pancreatitis, presence of (a) pancreatic or peri-ampullary benign or malignant tumor(s), characteristics of auto-immune pancreatitis, anatomic variations (e.g. pancreas divisum) or other anomalies (e.g. cholecystitis, vascular, renal, splenic or hepatic anomalies or ascites), and performance of fine needle aspiration or fine needle biopsy. Additionally, the type of endoscope, use of sedation, procedure related complications and results of the fine needle aspiration or biopsy will be systematically recorded by the study coordinator in a separate CRF.

Primary outcome measure

The primary outcome measure is the number and ratio of patients with presumed IAP in whom EUS detects a cause for the pancreatitis episode.

A positive EUS is defined as an EUS during which a definitive cause for the acute pancreatitis episode has been found; or during which abnormalities are visualized constituting a definitive cause,

For peer review only - http://bmjopen.bmj.com/site/about/guidelines.xhtml

after obtaining tissue and pathological examination. An overview of the exact findings scored as positive imaging is provided in table 3.

If during EUS pancreatic abnormalities are found, yet not enough to make a certain diagnosis of chronic pancreatitis according to the M-ANNHEIM classification (21), this imaging is considered to be negative, even though it did show abnormalities. This approach is chosen because the aim of this study is to determine the rate of which EUS can find a cause for the presumed IAP episode. For the same reason, report of an anatomical abnormality during EUS after a first episode of acute pancreatitis is not scored as positive imaging as pancreatic morphological changes are very common in IAP and not necessarily clinically relevant, as is elaborated on in the discussion (23).

Secondary outcome measures

The secondary outcome measures are recurrence rate of acute pancreatitis, severity of recurrent pancreatitis (20), readmission, performance of additional invasive procedures (e.g. cholecystectomy, endoscopic sphincterotomy), complications of EUS and of additional interventions, according to the Clavien-Dindo classification (24), length of hospital stay, quality of life, mortality and costs. Relevant definitions are reported in Additional File 2.

Sample size calculation

The sample size calculation was based on the primary outcome measure, diagnostic yield of EUS. Based on two previous studies reporting yield in patients with a first episode of presumed IAP (25, 26), adjusted for the PICUS study criteria for inclusion (i.e. requiring negative imaging after clinical recovery) and for positive imaging (i.e. excluding pancreas divisum as etiology), diagnostic yield was assumed to be 30%. Using a two-sided significance level (α) of 0.05, a power ($1 - \beta$) of 80%, 95 patients are needed to attain a 95% confidence interval (CI) with a range smaller than 10% above and below

251 the assumed yield of 30% (95% CI: 20.8, 39.2). Assuming a drop-out rate of 10%, a total of 106 patients
252 will be included (27). The sample size was calculated using the software programs RStudio (28) and
253 nQuery (29).

254

255 ## Follow-up

256 Data from patient records on primary and secondary outcome measures will be collected until 1 year
257 after inclusion. Outpatient care and follow-up after the EUS is at the discretion of the treating
258 physician, but an outpatient clinic visit after EUS to discuss the results of the EUS and potential
259 subsequent appropriate treatment can be considered standard care.

260 In case of biliary disease, the patient will be considered for endoscopic retrograde
261 cholangiopancreaticography (ERCP) with sphincterotomy when choledocho(-micro-)lithiasis or sludge
262 in the common bile duct is present, and cholecystectomy, as is standard care for biliary pancreatitis. A
263 (secretin-enhanced) MRCP will be recommended, if not performed earlier, if a patient is readmitted
264 for a recurrent episode of acute pancreatitis after a negative EUS for etiology, in order to rule out
265 structural anomalies such as pancreas divisum. This is in accordance with current guidelines (11).

266 Patients will be asked to fill out the Short Form-36 questionnaire in the validated Dutch
267 translation on day 3 after inclusion, after 6 months and after 1 year. This questionnaire in both English
268 and Dutch is included in additional file 3.

269

270 ## Statistical aspects

271 All included subjects will be evaluated for primary and secondary endpoints until 1 year after inclusion.
272 The primary analysis will be based on intention-to-treat principles. For exploratory reasons a per-
273 protocol analysis will be performed too.

For peer review only

For peer review only - http://bmjopen.bmj.com/site/about/guidelines.xhtml

The intention-to-treat population comprises all patients included in the study, regardless of adherence to study protocol. The per-protocol population is the subset of included patients who were treated with the guidelines of the protocol (i.e. meeting all eligibility criteria including all of the diagnostic tests required for the diagnosis of IAP, undergoing EUS as described in the "Endoscopic ultrasonography section"). A tabular listing of all patients excluded from the intention-to-treat population will be provided together with the reasons for exclusion.

All analyses will be performed in SPSS for Microsoft Windows. All data handling and analysis will be saved in a syntax-file. Results will be presented with all centers combined. A two-tailed p-value of < 0.05 is considered statistically significant.

Baseline variables

The reported baseline characteristics consist of age, sex, body mass index (BMI), previous cholecystectomy, nicotine and alcohol use, severity of pancreatitis, length of hospital stay, amylase, lipase, C-reactive protein, alanine transaminase, calcium, albumin and triglycerides levels in blood serum on admission, imaging modalities before EUS and their findings. Baseline characteristics of EUS will include timing of EUS, experience of endosonographist and type of sedation and type of endoscope used. Data will be presented in percentages or as mean with standard deviation, or in case of a skewed distribution as median with interquartile range (IQR).

Primary outcome measure: etiology detection rate

Overall detection rate of an etiology for the episode of acute pancreatitis will be presented as percentage with a 95% CI. Predefined subgroup analyses will be made for patients with and without obesity (cut-off at a BMI of 30), a previous cholecystectomy, alcohol use and local complications from the IAP episode. A subgroup analysis will also be made for patients with a transabdominal ultrasound

For peer review only - http://bmjopen.bmj.com/site/about/guidelines.xhtml

as imaging after clinical recovery and with magnetic resonance imaging (MRI) or MRCP as imaging after clinical recovery. Finally, a subgroup analysis will be made for EUS performed by endosonographists with and without extensive experience (cut-off at 400 endosonographies performed), use of linear or radial scope and type of sedation used. In subgroup analyses, the Chi-square test or the Fisher's exact test will be used, as appropriate, to compare etiology detection rate between subgroups. In subgroup analyses, comparability between groups regarding baseline variables will be checked. If the subgroups differ statistically significantly in one or more baseline variables, this will be corrected in a logistic regression analysis.

Secondary outcome measures

Secondary outcome measures will be described as percentages with 95% CI, as mean with standard deviation or median with IQR, as appropriate.

For recurrence rate, subgroup analyses will be made for patients with a positive and negative EUS, and in patients with a positive EUS, for patients who were and were not treated adequately. The same subgroup analyses as in the primary outcome measure, will also be applied on the recurrence rate. The Chi-square test or the Fisher's exact test will be used for comparison between subgroups, as appropriate.

For quality of life, subgroup analyses will be made for baseline versus follow-up quality of life and for patients with a positive and negative EUS, and with and without pancreatitis recurrence during follow-up. The (un-)paired T-test, Wilcoxon signed rank test or the Mann-Whitney U test will be used for comparisons between subgroups, as appropriate.

For peer review only - http://bmjopen.bmj.com/site/about/guidelines.xhtml

Cost analysis

The cost analysis will comprise direct medical costs, which are generated by healthcare utilization and include hospital admission periods and therapeutic and diagnostic procedures (30). Estimates of unit costs will be based on Dutch reference data from the cost guide of the Dutch Health Council (31). If this guide is an inappropriate determination of unit costs, the costs will be based on data provided by two hospital administrations (one university center and one general hospital) to account for the actual input of personnel, material and overhead over hospital resources used. Cost calculations will be used to determine cost of interventions (surgical, endoscopic or radiological) and diagnostic imaging. The cost analysis will be reported separately from the main study manuscript.

For peer review only

Patient and public involvement

The patient advocacy organization *'Alvleeskliervereniging Nederland'* was involved in the design of the PICUS study. The experience of the patient advocacy organization with IAP and participation in scientific research has driven the research question and design of the study with regards to patient burden. The patient advocacy organization will also be involved in the dissemination and implementation of the study results.

All patients eligible for participation will be asked to give written informed consent.

Ethics and dissemination

The PICUS study is conducted according to the principles of the Declaration of Helsinki (October 2013) and to the Guideline for Good Clinical Practice by the International Council for Harmonization (November 9 2016).

For peer review only - http://bmjopen.bmj.com/site/about/guidelines.xhtml

343 The need for ethical approval was waived by the Medical Ethics Review Committee of the Academic Medical Center on May 28, 2018 (W18_161 # 18.199), by the Medical Research Ethics Committee of the University Medical Center Utrecht on July 04, 2018 (18-469), by the Research Ethics Committee of Radboud university medical center on July 23, 2018 (2018-4520), by the Medical Ethics Review Committee of the Erasmus Medical Center on July 30, 2018 (MEC-2018-1293) and by the Medical Ethics Review Committee of the Maastricht University Medical Center on September 7, 2018 (2018-0685). Before start of inclusion, local board approval will be obtained in all participating centers.

350 The results of the PICUS study will be submitted for publication in an international peer-reviewed scientific journal, regardless of study outcomes.

352

353

354 ## Discussion

355 Previous research has suggested that EUS might be beneficial in the detection of an etiology in presumed IAP. However, data lacks on the efficacy of routine EUS in patients with a first episode of presumed IAP, after repeat imaging after clinical recovery is negative for an etiology. The PICUS study aims to determine whether routine EUS is warranted in a first episode of acute pancreatitis where no cause could be uncovered after complete standard diagnostic work-up.

360 Currently, guidelines do not clearly define criteria for biliary origin (11). However, it is generally agreed upon that cholelithiasis, microlithiasis or biliary sludge constitute biliary etiology. Several previous studies have shown an association between elevated ALT levels and acute biliary pancreatitis (32-35), with a positive predictive value of 85% for an ALT > 150 U/L within 48 hours after onset of symptoms (11, 32, 33, 35). Therefore, an elevated blood serum ALT level at admission is considered to entail a high probability of biliary etiology, and pancreatitis with an elevated ALT is treated as being of biliary origin (32-34, 36). However, the majority of current literature on EUS did not exclude patients

For peer review only - http://bmjopen.bmj.com/site/about/guidelines.xhtml

based on ALT level at admission (15, 25, 26, 32, 37-46). As these patients have a higher a priori chance of confirmation of biliary etiology on EUS, the etiology detection rate of EUS might be overestimated in these studies. In PICUS, biliary etiology is defined as either the signs of cholelithiasis, microlithiasis or biliary sludge on transabdominal ultrasonography, or transient elevation of the blood serum ALT level of more than twice the upper limit of normal at admission in the absence of ALT elevating comorbidity. By only including patients with normal or slightly elevated ALT levels at admission, the etiology detection rate as reported in PICUS will reflect the detection rate in patients who are truly considered as having presumed IAP after standard diagnostic work-up.

Multiple definitions for IAP are maintained in literature (47). For PICUS, the definition provided by the IAP/APA evidence-based guidelines on management of acute pancreatitis was used (11). These guidelines advise a repeat transabdominal ultrasound after clinical recovery in the work-up of presumed IAP because the index transabdominal ultrasound is less sensitive during the acute phase of pancreatitis. The subpar visualization of gall bladder, bile ducts and pancreas is often due to excessive amounts of air in the intestines caused by pancreatitis-induced ileus and/or suboptimal cooperation of painful patients (48). After the first episode of acute pancreatitis, repeating a transabdominal ultrasound may be able to detect biliary stones where it could not during index admission (49). Of the current literature on EUS in IAP, however, only a minority of studies included repeat imaging in the diagnostic work-up before EUS (15, 40, 41, 43). Previous research has shown that a repeat transabdominal ultrasound has a diagnostic yield of 20% in patients with a first episode of IAP (13). Omitting repeat imaging from diagnostic work-up before EUS may lead to an overestimation of the diagnostic yield of EUS. In PICUS, all patients are required to undergo imaging after clinical recovery, i.e. transabdominal ultrasound or MRI/MRCP. Computed tomography (CT) is not considered sufficient imaging as biliary disease, the most common underlying etiology in presumed IAP, cannot always be adequately detected using CT.

For peer review only

It is well documented that the overall diagnostic yield of EUS in patients with recurrent pancreatitis is superior to the diagnostic yield of both secretin-enhanced MRCP (s-MRCP) and non-secretin-enhanced MRCP (18, 44, 46, 50). In the subgroup of patients with a pancreas divisum, however, s-MRCP is considered to be superior in diagnostic yield to both EUS and MRCP (18). The role of pancreas divisum in the etiology of pancreatitis is unclear. Epidemiological studies have shown that the prevalence of pancreas divisum in the general population is equal to the prevalence in patients with presumed IAP (23). In patients with a pancreas divisum and acute pancreatitis, potentially other disease modifying factors add to the occurrence of pancreatitis, such as increased sensitivity to toxins or genetic susceptibility. Because of this ambiguity, pancreas divisum in patients with a first episode of acute pancreatitis is mostly left untreated in clinical practice. However, if patients with a pancreas divisum present with multiple episodes of presumed IAP, the divisum is often considered to be related to the pancreatitis and is subsequently treated, often with ERCP with endoscopic sphincterotomy, although evidence supporting this practice is limited (23). Because of both the diagnostic superiority of EUS in recurrent pancreatitis as well as the lack of clinical consequences of (s-)MRCP in patients with a first episode of pancreatitis, EUS is preferred to (s-)MRCP as the first choice for additional diagnostic testing for etiology in patients with presumed IAP (18, 44, 46, 50). Subsequently, current guidelines advise performing MRCP in case of recurrent IAP after EUS fails to determine an etiology (11). Therefore, in PICUS, we have chosen not to systematically include (s-)MRCP in the diagnostic work-up before EUS of first episode IAP.

Current guidelines advise consideration of EUS after a first or second attack of IAP (11). However, there is a paucity of evidence on the efficacy of EUS in first episode IAP. Three previous studies prospectively reported on EUS in patients with first episode IAP (25, 26, 38). However, in these studies, patients were not excluded based on liver enzymes abnormalities suggestive of biliary disease and no repeat imaging after clinical recovery was performed. PICUS will be the first prospective cohort study in which EUS will be performed in patients with a first episode of IAP after complete standard diagnostic work-up before EUS according to current guidelines (11).

For peer review only - http://bmjopen.bmj.com/site/about/guidelines.xhtml

A diagnostic yield of 10% for any etiology will be considered reasonable to justify incorporating routine EUS after a first episode of presumed IAP. This cut-off value was determined during a multidisciplinary meeting of the Dutch Pancreatitis Study Group, which included the principal investigators of several trials being executed by the Dutch Pancreatitis Study Group. Considering the expectation that the majority of uncovered etiologies by EUS will be treatable (e.g. biliary disease) and adequate treatment could prevent pancreatitis recurrence, while in a minority of uncovered etiologies diagnosis before progression of disease might be crucial for prognosis (e.g. malignancy), a positive result in 10% of patients was deemed sufficient to warrant routine EUS after a first episode of presumed IAP.

In conclusion, the PICUS study is the first prospective cohort study of patients with a single episode of presumed IAP after complete standard diagnostic work-up (including exclusion based on blood serum ALT and imaging after clinical recovery). The results of the PICUS study will establish whether routine EUS should be incorporated in the guidelines for standard diagnostic work-up after a first episode of presumed IAP.

For peer review only

Acknowledgements

The authors would like to acknowledge:

1. all of the members of the Dutch Pancreatitis Study Group, for their continuous efforts in recruitment of patients,
2. dr. S. van Dieren, for providing her expertise in the calculation of the sample size,
3. dr. M. van der Vlugt, MD, PhD, for her willingness to act as independent physician for the PICUS study, and

For peer review only - http://bmjopen.bmj.com/site/about/guidelines.xhtml

4. the pancreatic disease patient advocacy organization *Alvleeskliervereniging Nederland*, for their effort in representing the perspective of (participating) patients during the study design.

References

1. Nesvaderani M, Eslick GD, Vagg D, Faraj S, Cox MR. Epidemiology, aetiology and outcomes of acute pancreatitis: A retrospective cohort study. Int J Surg. 2015;23(Pt A):68-74.
2. van Brunschot S, van Grinsven J, van Santvoort HC, Bakker OJ, Besselink MG, Boermeester MA, et al. Endoscopic or surgical step-up approach for infected necrotising pancreatitis: a multicentre randomised trial. The Lancet. 2018;391(10115):51-8.
3. Bakker OJ, van Brunschot S, van Santvoort HC, Besselink MG, Bollen TL, Boermeester MA, et al. Early versus on-demand nasoenteric tube feeding in acute pancreatitis. N Engl J Med. 2014;371(21):1983-93.
4. Besselink MGH, van Santvoort HC, Buskens E, Boermeester MA, van Goor H, Timmerman HM, et al. Probiotic prophylaxis in predicted severe acute pancreatitis: a randomised, double-blind, placebo-controlled trial. The Lancet. 2008;371(9613):651-9.
5. Bakker OJ, van Santvoort HC, van Brunschot S, Geskus RB, Besselink MG, Bollen TL, et al. Endoscopic Transgastric vs Surgical Necrosectomy for Infected Necrotizing Pancreatitis A Randomized Trial. JAMA. 2012;307(10):1053-61.
6. Van Santvoort HC, Besselink MG, Bakker OJ, Hofker HS, Boermeester MA, dejong CH, et al. A Step-up Approach or Open Necrosectomy for Necrotizing Pancreatitis. N Engl J Med. 2010;362:1491-502.
7. Sadr-Azodi O, Oskarsson V, Discacciati A, Videhult P, Askling J, Ekbom A. Pancreatic Cancer Following Acute Pancreatitis: A Population-based Matched Cohort Study. Am J Gastroenterol. 2018;113(11):1711-9.
8. Zheng Y, Zhou Z, Li H, Li J, Li A, Ma B, et al. A Multicenter Study on Etiology of Acute Pancreatitis in Beijing During 5 Years. Pancreas. 2015;44(3):409-14.
9. Zhu Y, Pan X, Zeng H, He W, Xia L, Liu P, et al. A Study on the Etiology, Severity, and Mortality of 3260 Patients With Acute Pancreatitis According to the Revised Atlanta Classification in Jiangxi, China Over an 8-Year Period. Pancreas. 2017;46(4):504-9.
10. Roberts SE, Morrison-Rees S, John A, Williams JG, Brown TH, Samuel DG. The incidence and aetiology of acute pancreatitis across Europe. Pancreatology. 2017;17(2):155-65.
11. Working Group IAP/APA Acute Pancreatitis Guidelines. IAP/APA evidence-based guidelines for the management of acute pancreatitis. Pancreatology. 2013;13(4 Suppl 2):e1-15.
12. UK Working Party on Acute Pancreatitis. UK guidelines for the management of acute pancreatitis. Gut. 2005;54 Suppl 3:iii1-9.
13. Hallensleben ND, Umans DS, Bouwense SA, Verdonk RC, Romkens TE, Witteman BJ, et al. The diagnostic work-up and outcomes of 'presumed' idiopathic acute pancreatitis: A post-hoc analysis of a multicentre observational cohort. United European Gastroenterol J. 2020;8(3):340-50.
14. Lee SP, Nicholls JF, Park HZ. Biliary Sludge as a Cause of Acute Pancreatitis. N Engl J Med. 1992;326:589-93.
15. Garg PK, Tandon RK, Madan K. Is biliary microlithiasis a significant cause of idiopathic recurrent acute pancreatitis? A long-term follow-up study. Clin Gastroenterol Hepatol. 2007;5(1):75-9.
16. Tenner S, Baillie J, DeWitt J, Vege SS, American College of G. American College of Gastroenterology guideline: management of acute pancreatitis. Am J Gastroenterol. 2013;108(9):1400-15; 16.

For peer review only

For peer review only - http://bmjopen.bmj.com/site/about/guidelines.xhtml

17. Banks PA, Freeman ML, Practice Parameters Committee of the American College of G. Practice guidelines in acute pancreatitis. Am J Gastroenterol. 2006;101(10):2379-400.
18. Wan J, Ouyang Y, Yu C, Yang X, Xia L, Lu N. Comparison of EUS with MRCP in idiopathic acute pancreatitis: a systematic review and meta-analysis. Gastrointest Endosc. 2018.
19. Schünemann H BJ, Guyatt G, Oxman A, editors. GRADE handbook for grading quality of evidence and strength of recommendations. Group TGW, editor2013.
20. Banks PA, Bollen TL, Dervenis C, Gooszen HG, Johnson CD, Sarr MG, et al. Classification of acute pancreatitis--2012: revision of the Atlanta classification and definitions by international consensus. Gut. 2013;62(1):102-11.
21. Schneider A, Lohr JM, Singer MV. The M-ANNHEIM classification of chronic pancreatitis: introduction of a unifying classification system based on a review of previous classifications of the disease. J Gastroenterol. 2007;42(2):101-19.
22. Hawes RH, Fockens P. Endosonography. Second edition ed. Philadelphia: Elsevier Inc.; 2011.
23. DiMagno MJ, Wamsteker EJ. Pancreas divisum. Curr Gastroenterol Rep. 2011;13(2):150-6.
24. Dindo D, Demartines N, Clavien P-A. Classification of Surgical Complications. Annals of Surgery. 2004;240(2):205-13.
25. Yusoff IF, Raymond G, Sahai AV. A prospective comparison of the yield of EUS in primary vs. recurrent idiopathic acute pancreatitis. Gastrointest Endosc. 2004;60(5):673-8.
26. Vila JJ, Vicuna M, Irisarri R, de la Higuera BG, Ruiz-Clavijo D, Rodriguez-Gutierrez C, et al. Diagnostic yield and reliability of endoscopic ultrasonography in patients with idiopathic acute pancreatitis. Scand J Gastroenterol. 2010;45(3):375-81.
27. Whitley E, Ball J. Statistics review 4: Sample size calculations. Critical Care 2002;6(4):335-41.
28. R-Core-Team. R: A Language and Environment for Statistical Computing. Vienna, Austria: The R Foundation for Statistical Computing; 2017.
29. Oliver MR, Yeghiazarian K, Zheng M, Jamshidian M, Koyfman I. nQuery Advisor. 1995-2007.
30. Gold MR, Siegel JE, Russel LB. Cost-effectiveness in Health and Medicine. New York: Oxford University Press; 1996.
31. Zorginstituut Nederland (Health Institute Netherlands). Kostenhandleiding: Methodologie van kostenonderzoek en referentieprijzen voor economische evaluaties in de gezondheidszorg (Cost Guide: Methology of cost analysis and reference prices for economical evaluations in health care) 2015.
32. Liu CL, Fan ST, Lo CM, Tso WK, Wong Y, Poon RT, et al. Clinico-biochemical prediction of biliary cause of acute pancreatitis in the era of endoscopic ultrasonography. Aliment Pharmacol Ther. 2005;22(5):423-31.
33. Moolla Z, Anderson F, Thomson SR. Use of amylase and alanine transaminase to predict acute gallstone pancreatitis in a population with high HIV prevalence. World J Surg. 2013;37(1):156-61.
34. Ammori BJ, Boreham B, Lewis P, Roberts SA. The Biochemical Detection of Biliary Etiology of Acute Pancreatitis on Admission: A Revisit in the Modern Era of Biliary Imaging. Pancreas. 2003;26:e32-5.
35. Tenner SD, H.; Steinberg, W. Predicting gallstone pancreatitis with laboratory parameters: a meta-analysis. Am J Gastroenterol. 1994;89:1863-6.
36. Schepers NJ, Bakker OJ, Besselink MG, Bollen TL, Dijkgraaf MG, van Eijck CH, et al. Early biliary decompression versus conservative treatment in acute biliary pancreatitis (APEC trial): study protocol for a randomized controlled trial. Trials. 2016;17:5.
37. Frossard JL, Sosa-Valencia L, Amouyal G, Marty O, Hadengue A, Amouyal P. Usefulness of Endoscopic Ultrasonography in Patients with "Idiopathic" Acute Pancreatitis. Am J Med. 2000;109:196-200.
38. Govil A, Agrawal MK, Agrawal D, Udawat H. Role of endoscopic ultrasonography in patients with first episode of idiopathic acute pancreatitis. Indian J Gastroenterol. 2014;33(3):241-8.
39. Mariani A, Arcidiacono PG, Curioni S, Giussani A, Testoni PA. Diagnostic yield of ERCP and secretin-enhanced MRCP and EUS in patients with acute recurrent pancreatitis of unknown aetiology. Dig Liver Dis. 2009;41(10):753-8.

For peer review only

For peer review only - http://bmjopen.bmj.com/site/about/guidelines.xhtml

40. Morris-Stiff G, Al-Allak A, Frost B, Lewis WG, Puntis MC, Roberts A. Does Endoscopic Ultrasound Have Anything to Offer in the Diagnosis of Idiopathic Acute Pancreatitis? JOP. 2009;10(2):143-6.

41. Norton SA, Alderson D. Endoscopic ultrasonography in the evaluation of idiopathic acute pancreatitis British Journal of Surgery. 2000;97:1650-5.

42. Queneau P-E, Zeeh S, Lapeyre V, Thibault P, Heyd B, Carayon P, et al. Feasibility of and Interest in Combined Endoscopic Ultrasonography and Biliary Drainage in Unexplained Acute Biliopancreatic Disorders. Dig Dis Sci. 2002;47(9):2020-4.

43. Rana SS, Bhasin DK, Rao C, Singh K. Role of endoscopic ultrasound in idiopathic acute pancreatitis with negative ultrasound, computed tomography, and magnetic resonance cholangiopancreatography. Ann Gastroenterol. 2012;25(2):133-7.

44. Repiso Ortega A, Gómez-Rodríguez R, Romero M, Fernández-Zapardiel S, del Mar Céspedes M, Carrobles J-M. Prospective Comparison of Endoscopic Ultrasonography and Magnetic Resonance Cholangiopancreatography in the Etiological Diagnosis of ‘‘Idiopathic’’ Acute Pancreatitis. Pancreas. 2011;40:289-94.

45. Tandon M, Topazian M. Endoscopic Ultrasound in Idiopathic Acute Pancreatitis. Am J Gastroenterol. 2001;96(3):705–9.

46. Thevenot A, Bournet B, Otal P, Canevet G, Moreau J, Buscail L. Endoscopic ultrasound and magnetic resonance cholangiopancreatography in patients with idiopathic acute pancreatitis. Dig Dis Sci. 2013;58(8):2361-8.

47. Somani P, Sunkara T, Sharma M. Role of endoscopic ultrasound in idiopathic pancreatitis. World J Gastroenterol. 2017;23(38):6952-61.

48. Lara LF, Levy MJ. Idiopathic Recurrent Acute Pancreatitis. MedGenMed. 2004;6(4):10.

49. Signoretti M, Baccini F, Piciucchi M, Iannicelli E, Valente R, Zerboni G, et al. Repeated Transabdominal Ultrasonography Is a Simple and Accurate Strategy to Diagnose a Biliary Etiology of Acute Pancreatitis. Pancreas. 2014;43(7):1106-10.

50. Meeralam Y, Al-Shammari K, Yaghoobi M. Diagnostic accuracy of EUS compared with MRCP in detecting choledocholithiasis: a meta-analysis of diagnostic test accuracy in head-to-head studies. Gastrointest Endosc. 2017;86(6):986-93.

51. Stolle M, Sack PM, Thomasius R. Binge drinking in childhood and adolescence: epidemiology, consequences, and interventions. Dtsch Arztebl Int. 2009;106(19):323-8.

52. Sadr Azodi O, Orsini N, Andren-Sandberg A, Wolk A. Effect of type of alcoholic beverage in causing acute pancreatitis. Br J Surg. 2011;98(11):1609-16.

53. Midanik LT. Drunkenness, feeling the effects and 5+ measures. Addiction. 1999;94(6):887- 97.

54. Ratjen F, Döring G. Cystic fibrosis. The Lancet. 2003;361(9358):681-9.

55. Spanier B, Bruno MJ, Dijkgraaf MG. Incidence and mortality of acute and chronic pancreatitis in the Netherlands: a nationwide record-linked cohort study for the years 1995-2005. World J Gastroenterol. 2013;19(20):3018-26.

56. CBS StatLine. Households; size, position in household, January 1st 1995-2013 StatLine: Central Bureau for Statistics 2015 [Available from: http://statline.cbs.nl/Statweb/publication/?VW=T&DM=SLNL&PA=37312&D1=a&D2=0,5,10,(l-2)-l&HD=180221-1109&HDR=G1&STB=T.

57. Whitcomb DC. Genetic aspects of pancreatitis. Annu Rev Med. 2010;61:413-24.

58. Whitcomb DC. Hereditary diseases of the pancreas. In: Yamada T AD, Kaplowitz N, Laine L, Owyang C, Powell DW, editors, editor. Textbook of Gastroenterology. 4th ed. Philadelphia: Lippincott Williams & Wilkins; 2003. p. 2147–65.

59. Shane E, Dinaz I. Hypercalcemia: Pathogenesis, clinical manifestations, differential diagnosis and management. In: Favus M, editor. Primer on the metabolic bone diseases and disorders of mineral metabolism. 6th ed. Philadelpiha: Kippincott, Williams, and Wilkins; 2006. p. 26-176.

60. Berglund L, Brunzell JD, Goldberg AC, Goldberg IJ, Sacks F, Murad MH, et al. Evaluation and treatment of hypertriglyceridemia: an Endocrine Society clinical practice guideline. J Clin Endocrinol Metab. 2012;97(9):2969-89.

For peer review only

For peer review only - http://bmjopen.bmj.com/site/about/guidelines.xhtml

61. Nitsche C, Maertin S, Scheiber J, Ritter CA, Lerch MM, Mayerle J. Drug-induced pancreatitis. Curr Gastroenterol Rep. 2012;14(2):131-8.
62. Lankisch PG, Apte M, Banks PA. Acute pancreatitis. The Lancet. 2015;386(9988):85-96.
63. Tummala P, Tariq SH, Chibnall JT, Agarwal B. Clinical Predictors of Pancreatic Carcinoma Causing Acute Pancreatitis. Pancreas. 2013;42(1):108-13.
64. Cotton PB, Lehman G, Vennes J, Geenen JE, Russell RCG, Meyers WC, et al. Endoscopic sphincterotomy complications and their management: an attempt at consensus. Gastrointest Endosc. 1991;37:383-93.
65. Connor S. Defining post-operative pancreatitis as a new pancreatic specific complication following pancreatic resection. HPB (Oxford). 2016;18(8):642-51.
66. Booth F, Flint L. Pancreatoduodenal trauma. In: ed BJ, editor. Blunt multiple trauma. New York: Marcel Dekker; 1990. p. 497-509.
67. Smith I, Monkemuller K, Wilcox CM. Incidentally Identified Common Bile Duct Dilatation. A Systematic Review of Evaluation, Causes, and Outcome. J Clin Gastroenterol. 2015;49:810-5.
68. Mortelé KJ, Rocha TC, Streeter JL, Taylor AJ. Multimodality Imaging of Pancreatic and Biliary Congenital Anomalies. Radiographics. 2006;26(3):715-31.

Author Statement

Authors' contributions

DSU drafted the manuscript. HCT, RCV, SAB, MGB and JEvH co-authored the writing of the manuscript.

DSU, RCV, SAB, MABo, MJB, PF, EJMvG, JWP, HCvS, FPV, MGB and JEvH were involved in the design of the study during several meetings of the Dutch Pancreatitis Study Group. NDHL, MPGFA, AB, RAB, MABr, LH, WLC, HMvD, BCvE, GWE, WLH, CVH, AI, LMK, SDK, LEP, RQ, TEHR, ACITLT, AYT, NGV, AMCJV, RLJvW and BJW critically assessed the study design, during several meetings, and edited the manuscript. All authors read and approved the final manuscript.

Funding statement

This work was supported by the Dutch Digestive Disease Foundation (*Maag Lever Darm Stichting*, grant number D17-25). The PICUS study is an investigator-initiated study. The sponsor had no influence on design, implementation and conduct of the study, as well as on collection, analysis and interpretation of data, construction of the manuscript and decision to publish.

For peer review only - http://bmjopen.bmj.com/site/about/guidelines.xhtml

Competing interests statement

The authors declare that they have no competing interests.

Data Availability Statement

The datasets used and/or analyzed during the current study are available from the corresponding author on reasonable request.

For peer review only

Figure legend

Figure 1: Overview of screening and study procedures. MRI = magnetic resonance imaging. MRCP = magnetic resonance cholangiopancreaticography. CRF = Case Report Form. EUS = endoscopic ultrasonography.

For peer review only - http://bmjopen.bmj.com/site/about/guidelines.xhtml

Table 1

**Standard diagnostic work-up**	
Detailed personal and family history, including questions on:	Alcohol use
	Recent ERCP
	Recent start or changes in use of drugs associated with acute pancreatitis
	Recent major abdominal trauma
	Recent abdominal surgery
	Familial and hereditary pancreatitis
	Cystic fibrosis-related pancreatitis
Laboratory tests, including:	Blood serum triglycerides level
	Blood serum calcium level, corrected for the blood serum albumin level
	Blood serum ALT level on admission
Imaging:	Transabdominal ultrasound, MRI or MRCP after clinical recovery

Table 1: Standard diagnostic work-up *Standard diagnostic work-up according to the 2013 IAP/APA evidence-based guideline on management of acute pancreatitis. A listing of the drugs considered to be associated with acute pancreatitis are listed in additional file 1. ERCP = endoscopic retrograde cholangiopancreaticography; ALT = alanine aminotransferase; MRI = magnetic resonance imaging; MRCP = magnetic resonance cholangiopancreaticography.*

For peer review only

651 Table 2

Etiology	Definition
Alcohol	> 4 units of alcohol in the 24 hours prior to start of abdominal complaints (51-53)
Biliary disease	1. A transient elevated ALT level of >2 times the upper limit of normal at diagnosis of acute pancreatitis, in the absence of other ALT elevating comorbidity (34), OR<br>2. Gallstones, microlithiasis and/or biliary sludge, OR<br>3. A dilated CBD of >8 mm in patients <76 years or >10 mm in patients >75 years at diagnosis of acute pancreatitis (36)
Cystic fibrosis	history of cystic fibrosis in the absence of another origin (54)
Familial	two or more direct blood-related family members (parents, children or siblings) who have had an episode of acute pancreatitis (55-57)
Hereditary	mutation in the PRSS1, SPINK1, CFTR, CTRC, CLDN2 or CPA1 gene, or direct family member (parents, children, siblings) with one or more of the above mentioned mutations and at least one direct family member who has (had) acute or chronic pancreatitis (57, 58)
Hypercalcemia	blood serum calcium level ≥12 mg/dl (3 mmol/l), corrected for serum albumin level, as first measured during admission (59)
Hypertriglyceridemia	blood serum triglyceride level of ≥1000 mg/dl (11.2 mmol/l) under fasting conditions, as first measured during admission (60)
Medication	use of drug(s) listed in additional file 1, which has or have been started or increased in dosage within a reasonable temporal sequence, in principle 1

For peer review only - http://bmjopen.bmj.com/site/about/guidelines.xhtml

	month before onset of pancreatitis, and has or have a positive dechallenge (a drug reaction that is confirmed by stopping the drug) (61, 62)
Neoplasm	Known hepatopancreatobiliary malignancy or known malignancy with metastases causing obstruction of the pancreatic duct (63)
ERCP	ERCP within 24 hours before diagnosis of pancreatitis (64)
Surgical	abdominal surgery within 24 hours prior to diagnosis of pancreatitis (65)
Trauma	typical blunt trauma to the upper abdomen and pancreatic trauma visible on imaging (66)

Table 2: potential etiologies and their definitions *Potential etiologies and their definitions. Side branch or mixed type intraductal papillary mucinous neoplasms without dilatation of the pancreatic duct and pancreas divisum will not be considered to be a causative factor for the pancreatitis episode. If imaging is not able to discriminate between gall bladder polyps or concrements, lesions smaller than 10 mm will not be considered an exclusion criterion. Lesions above 10 mm, irrespective of whether they are a polyp or a concrement, are an immediate indication for cholecystectomy, and these patients will be excluded from PICUS. ALT = alanine transaminase. CBD = common bile duct. ERCP = endoscopic retrograde cholangiopancreaticography.*

For peer review only

For peer review only - http://bmjopen.bmj.com/site/about/guidelines.xhtml

Table 3

Biliary pancreatitis	Presence of biliary stones, microlithiasis, or sludge
	Widened CBD, >8 mm in patients <76 years, or >10 mm in patients >75 years, in the absence of other CBD dilating factors (e.g. opioid use, distal stenosis, obstruction of external compression of CBD or papilla (67))
Chronic pancreatitis	Pancreatic calcifications
	> 4 of the following abnormal features of the pancreas:<br>1. Enlarged gland size<br>2. Cysts<br>3. Echo-poor lesions (focal areas of reduced echogenicity)<br>4. Echo-rich lesions (> 3 mm in diameter)<br>5. Accentuation of lobular pattern<br>6. Increased duct wall echogenicity<br>7. Irregularity of the main pancreatic duct<br>8. Dilation of the main pancreatic duct > 3.5 mm (68)<br>9. Visible side branches<br>10. Calcifications of the pancreatic duct
Neoplasms	Definitive diagnosis of pathological tissue after histological or cytological evaluation of specimen of an anomaly observed during EUS, e.g. hyperplastic or malignant tissue, or auto-immune inflammatory disease
	Main duct IPMN or mixed type IPMN causing dilatation of the pancreatic duct

Table 3: positive imaging *Definition of positive imaging. For each diagnosis, presence of one of the separately mentioned abnormalities is required to be considered as positive imaging. Specimen is not required to be obtained during EUS. Anatomical anomalies (e.g. divisum) are not considered a certain etiology in first episode IAP and therefore not considered as positive imaging. CBD = common bile duct. EUS = endoscopic ultrasonography. IPMN = intraductal papillary mucinous neoplasm.*

For peer review only

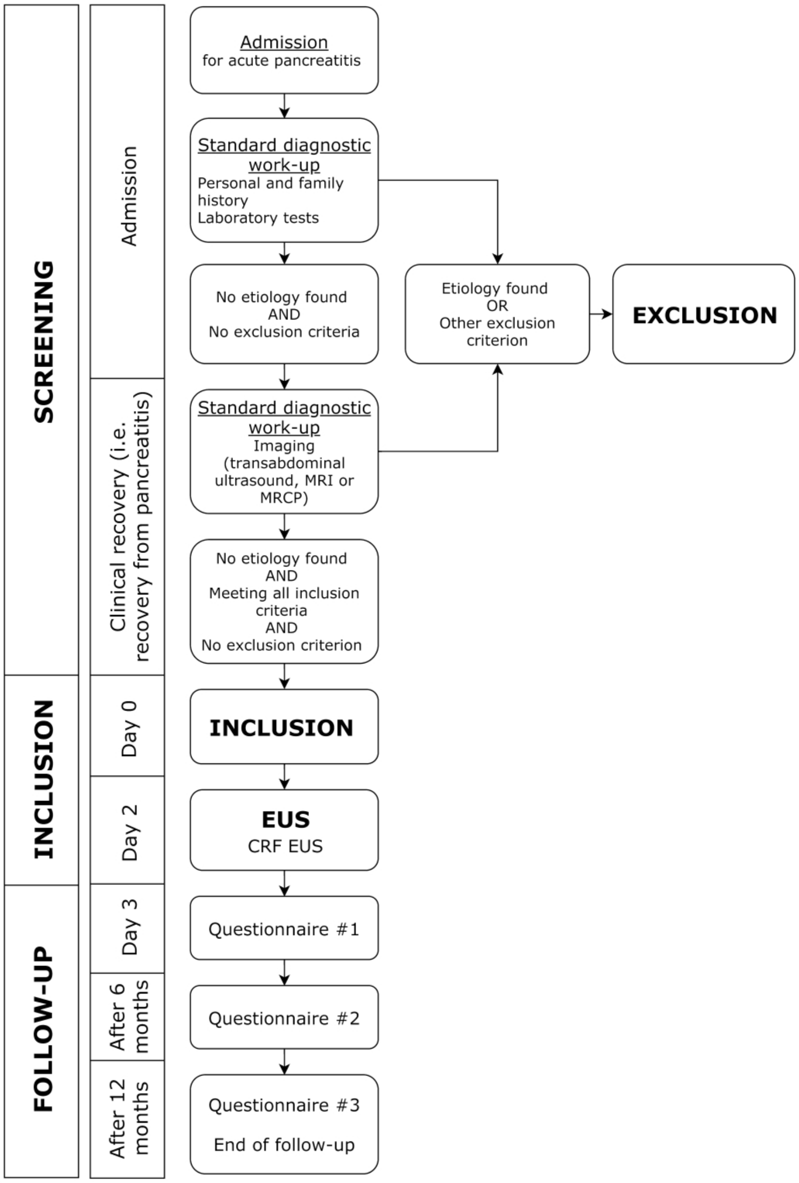

Overview of screening and study procedures. MRI = magnetic resonance imaging. MRCP = magnetic resonance cholangiopancreaticography. CRF = Case Report Form. EUS = endoscopic ultrasonography.

105x154mm (1200 x 1200 DPI)

Additional file 1: Table S1 Drugs associated with acute pancreatitis

Drugs associated with acute pancreatitis				
Acetaminophen	Cisplatin	Hydrochlorothiazide	Methyldopa	Pentavalent antimony compounds
Asparaginase	Cytarabine	Interferon alpha	Metronidazole	
Azathioprine	Didanosine	Itraconazole	Octreotide	Phenformin
Bortezomib	Enalapril	Lamivudine	Olanzapine	Simvastatin
Capecitabine	Erythromycin	Mercaptopurine	Opiates	Steroids
Carbamazepine	Estrogens	Mesalazine	Oxyphenbutazone	Sulfasalazine
Cimetidine	Furosemide	Olsalazine	Pentamidine	co-trimoxazole

Drugs with a definite association with acute pancreatitis (1, 2)

For peer review only

References

1. Nitsche C, Maertin S, Scheiber J, Ritter CA, Lerch MM, Mayerle J. Drug-induced pancreatitis. Curr Gastroenterol Rep. 2012;14(2):131-8.
2. Lankisch PG, Apte M, Banks PA. Acute pancreatitis. The Lancet. 2015;386(9988):85-96.

Additional file 2: Relevant definitions

Acute pancreatitis: an acute inflammation of the pancreatic parenchyma, diagnosed when at least two of the three following characteristics are present (1):

1. Clinical features of acute pancreatitis, such as upper abdominal pain
2. Elevated serum amylase or lipase levels of at least three times the upper limit of normal (ULN)
3. Signs of acute pancreatitis on imaging

Note: no value of the required serum amylase or lipase level is provided as every participating center has a local laboratory, which is why each center may use different normal range values.

Idiopathic acute pancreatitis is considered to be present if no etiology is found in standard work-up, according to the IAP/APA evidence-based guidelines on management of acute pancreatitis (2), which comprises at least the following tests:

1. A detailed personal and family history, including questions on:
    a. Alcohol use
    b. Recent endoscopic retrograde cholangiopancreaticography (ERCP)
    c. Recent start of or changes in use of drugs associated with acute pancreatitis
    d. Recent major abdominal trauma
    e. Recent abdominal surgery
    f. Familial pancreatitis
    g. Hereditary pancreatitis

For peer review only

    h. Cystic fibrosis related pancreatitis

2. Laboratory tests, including:
    a. Blood serum triglycerides level on admission
    b. Blood serum calcium level, corrected for the serum albumin level, on admission
    c. Blood serum alanine transaminase (ALT) level on admission
3. Imaging via transabdominal ultrasound, magnetic resonance imaging (MRI) or magnetic resonance cholangiopancreaticography (MRCP) after clinical recovery

Note: side branch or mixed type intraductal papillary mucinous neoplasms (IPMN) without dilatation of the pancreatic duct will not be considered to be a causative factor for the pancreatitis episode.

Note: if the imaging is not able to discriminate between gall bladder polyps or concrements, lesions smaller than 10 mm will not be considered an exclusion criterion. Lesions above 10 mm, irrespective of whether they are a polyp or a concrement, are an immediate indication for cholecystectomy, and will be excluded from PICUS.

Alcoholic pancreatitis: pancreatitis caused by an excess intake of alcohol, diagnosed when biliary etiology is not demonstrated by standard work-up and the patient has indicated (either by direct or indirect personal history or by findings during physical examination) to have drank at least five units of alcohol in the 24 hours prior to start of abdominal complaints (or in asymptomatic acute pancreatitis: prior to diagnosis) (3-5)

For peer review only

Biliary pancreatitis: pancreatitis caused by biliary stones, microlithiasis or sludge, diagnosed when one of the following features is present:

1. A transient elevated ALT level of more than two times the ULN at diagnosis of acute pancreatitis, in the absence of ALT elevating comorbidity (6)
2. Signs of presence of gallstones, microlithiasis or sludge on imaging, defined as follows:
    a. Gallstones, microlithiasis and/or biliary sludge, either in the gall bladder, ductus cysticus, intrahepatic bile ducts or in the common bile duct (CBD), and/or
    b. A CBD of more than eight mm in patients 75 years old or younger or more than ten mm in patients older than 75 years at diagnosis of acute pancreatitis (7)

Note: no value of the required serum ALT level is provided as the normal range values depend on the sex of the patient and as every participating center has a local laboratory, which is why each center may use different normal range values.

Chronic pancreatitis**:** a chronic inflammation of the pancreatic parenchyma, defined as typical clinical history of chronic pancreatitis (such as recurrent pancreatitis or abdominal pain, except for primary painless pancreatitis) and one or more of the following (8):

1. Pancreatic calcifications
2. Moderate or marked ductal lesions, defined as two or more of the following abnormal features on transabdominal ultrasound, computed tomography (CT) or MRI/MRCP, according to the Cambridge classification (9):
    a. Main pancreatic duct abnormalities, either enlargement or increased echogenicity of the duct wall (mandatory)
    b. Pancreatic enlargement

For peer review only

   c. Cavities
   d. Duct irregularities including intraductal fillings defects, calculi or duct obstruction
   e. Focal acute pancreatitis
   f. Parenchymal heterogeneity
   g. Irregularities of pancreatic head or body contour

3. Moderate or marked ductal lesions, defined as five or more of the following abnormal features on endoscopic ultrasonography (EUS):
   a. Enlarged gland size
   b. Cysts
   c. Echo-poor lesions (focal areas of reduced echogenicity)
   d. Echo-rich lesions (more than three mm in diameter)
   e. Accentuation of lobular pattern (e.g., echo-poor normal parenchyma surrounded by hyperechoic strands)
   f. Increased duct wall echogenicity
   g. Irregularity of the main pancreatic duct (e.g., with narrowing of the duct)
   h. Dilation of the main pancreatic duct
   i. Visible side branches (e.g., with dilation)
   j. Calcification (of the pancreatic duct)
4. Marked and persistent exocrine insufficiency defined as pancreatic steatorrhea markedly reduced by enzyme supplementation
5. Typical histology of an adequate histological specimen

For peer review only

Note: during initial diagnostic work-up during admission 'marked and persistent exocrine insufficiency' cannot be evaluated properly. Therefore this part of the definition of chronic pancreatitis will not be applicable during standard work-up. However, if the patient does show marked and persistent exocrine insufficiency during follow-up (either during the outpatient clinic visit after repeat transabdominal ultrasound or after the EUS), this will be considered to be diagnostic for chronic pancreatitis. The same is applicable for histology of an adequate histological specimen: this is not part of standard work-up, however, if a typical histological specimen is obtained during follow-up, this will be considered to be diagnostic for chronic pancreatitis.

Clinical recovery from acute pancreatitis: resolution of pancreatic inflammation, present when one of the following criteria is met:

1. Discharge from the hospital
2. Normal inflammation parameters in laboratory tests
3. No signs of pancreatic inflammation on imaging

Cystic fibrosis: an autosomal recessive disorder caused by a mutation in the CFTR gene, resulting in defective chloride channels in epithelial cells, diagnosed by either a concentration in sweat of chloride greater than 60 mmol/L on repeated analysis, confirmation of a CFTR gene mutation, or both (10).

Cystic fibrosis related pancreatitis: pancreatitis caused by defective ductular and acinar pancreatic secretion, diagnosed when a patient with a history of cystic fibrosis presents with an acute pancreatitis in the absence of another origin (10).

For peer review only

Familial pancreatitis: acute pancreatitis from any cause that occurs in a family with an incidence that is greater than would be expected by chance alone, given the size of the family and the standardized incidence of pancreatitis within the Dutch population, defined as acute pancreatitis in patients who have two or more direct blood-related family members (parents, children or siblings) who have had an episode of acute pancreatitis (11-13).

Fever: a body temperature of 38.5˚C or higher.

Hereditary pancreatitis: otherwise unexplained pancreatitis in an individual from a family in which the pancreatitis phenotype appears to be inherited through a disease-causing gene mutation expressed in an autosomal dominant pattern, defined as pancreatitis in patients with a known mutation in the PRSS1 gene, the SPINK1 gene, the CFTR gene, the CTRC gene, the CLDN2 gene or the CPA1 gene, or if the patient has a direct family member (parents, children, siblings) with one or more of the above mentioned mutations and has at least one direct family member who has had an episode of acute pancreatitis or has chronic pancreatitis (13, 14).

Hypercalcemic pancreatitis: acute pancreatitis caused by hypercalcemia and diagnosed when no signs of a biliary pancreatitis are found in standard work-up and the patient has a blood serum calcium level of at least 12 mg/dl or 3 mmol/l, corrected for the serum albumin level, as first measured during admission (15).

For peer review only

Hypertriglyceridemic pancreatitis: acute pancreatitis based on hypertriglyceridemia and diagnosed if a biliary etiology is not demonstrated by standard work-up and the patient has a blood serum triglyceride level of at least 1000 mg/dl (or 11.2 mmol/l) under fasting conditions, as first measured during admission (16).

Hypothermia: a body temperature of 35.9˚C or lower.

Infected (extra)pancreatic necrosis: presence of microorganisms in (extra-)pancreatic necrosis, confirmed by a positive culture obtained by means of fine needle aspiration or from the first drainage procedure or necrosectomy, the presence of gas in the (extra-)pancreatic collection on CT, or the presence of clinical signs of persistent sepsis or progressive clinical deterioration despite maximal support on the intensive care unit (ICU) without other causes for infection (ruled out should be: pneumonia, urinary tract infection, wound infection, endocarditis, abdominal sepsis or any other infection which could be suspected based on the individual patient's clinical presentation) (17).

Medication associated pancreatitis: acute pancreatitis is considered to be caused by drugs when a biliary cause is not demonstrated by standard work-up, the patient uses one or multiple drug(s) listed in table S1 in additional file 1, the drug has been started or increased in dosage within a reasonable temporal sequence, in principle 1 month before the onset of the pancreatitis, and has a positive dechallenge (a drug reaction that is confirmed by stopping the drug) (18, 19).

Microlithiasis: stones or concrements, smaller than four mm, in the gall bladder or the bile ducts (20).

For peer review only

154 Murphy's sign: the phenomenon where compression of the right upper quadrant causes the patient to catch their breath due to pain when taking a deep breath (21).

157 Pancreas divisum: a congenital malformation of the main pancreatic duct (Wirsung's duct) with two separate ducts (a separate ventral duct of Wirsung and a dorsal duct of Santorini) as opposed to one main duct (of Wirsung) (22).

161 Positive imaging: positive imaging is defined as imaging during which a definitive cause for the acute pancreatitis episode can be found; or during which abnormalities are visualized constituting a definitive cause, after obtaining tissue and pathological examination. So, if during EUS ductal abnormalities are found, yet not enough to make a certain diagnosis of chronic pancreatitis according to the M-ANNHEIM classification (8), this imaging is considered to be negative, even though it did show abnormalities. This approach is chosen because the aim of this study is to determine the rate of which EUS can find a causative factor for a previous acute pancreatitis episode. For the same reason, finding of an anatomical abnormality after a first episode of acute pancreatitis is not scored as positive imaging. An overview of the exact findings scored as positive imaging is provided in table 3 of the main manuscript.

172 Post-ERCP pancreatitis: pancreatitis caused by mechanical injury from instrumentation and hydrostatic injury from contrast injection during ERCP, diagnosed if a patient develops a pancreatitis within 24 hours of an ERCP without indications of another origin (23).

For peer review only

Postoperative pancreatitis: pancreatitis caused by perioperative hypoperfusion of the pancreas, diagnosed if a patient develops a pancreatitis within 24 hours of abdominal surgery in the absence of indications for another origin (24).

Posttraumatic pancreatitis: pancreatitis caused by pancreatic injury due to trauma to the abdomen, diagnosed when the patient describes a typical blunt trauma to the upper abdomen and pancreatic trauma is visible on imaging (25).

Recurrence rate: the risk of a recurrent episode of acute pancreatitis.

Sludge: solid material which results from the slow settling of particles dispersed in bile (20).

For peer review only

Standard work-up:

1. A detailed personal and family history, including questions on:
    a. Alcohol use
    b. Recent ERCP
    c. Recent start of or changes in use of drugs associated with acute pancreatitis
    d. Recent major abdominal trauma
    e. Recent abdominal surgery
    f. Familial pancreatitis
    g. Hereditary pancreatitis
    h. Cystic fibrosis related pancreatitis
2. Laboratory tests, including:
    a. Blood serum triglycerides level, first measured during admission
    b. Blood serum calcium level, corrected for the serum albumin level, first measured during admission
    c. Blood serum ALT level on admission
3. Imaging via transabdominal ultrasound, MRI or MRCP after clinical recovery

Biliary events: acute cholecystitis; biliary colic's requiring readmission; biliary pancreatitis; cholangitis; or obstructive choledocholithiasis needing ERCP.

For peer review only

Acute cholecystitis: an acute inflammation of the gall bladder, diagnosed when one item in A, B and C is present:

A) Local signs of inflammation

1. Murphy's' sign, or
2. Right upper abdominal quadrant mass, pain or tenderness

B) Systemic signs of inflammation

1. Fever or hypothermia, or
2. Elevated C-reactive protein CRP), or
3. Elevated white blood cell count

C) Imaging findings characteristic of acute cholecystitis (26, 27)

Note: acute cholecystitis and cholangitis (see definition below) are defined according to the Tokyo classification which defines fever as a body temperature of 38˚C or higher; however, fever will be defined in this study as hyperthermia of 38.5˚C or higher and hypothermia will be added as a systemic sign of inflammation, as this more accurately reflects clinical practice in the Netherlands.

Biliary colic: upper abdominal pain (either right upper quadrant or epigastric pain) lasting at least 30 minutes, often associated with restlessness (28).

For peer review only

Cholangitis: an inflammation of the bile duct(s), diagnosed when one item in each of the following categories is present:

1. Systemic inflammation
    a. Fever, hypothermia and/or shaking chills
    b. Laboratory data: evidence of inflammatory response (abnormal white blood cell counts (defined as smaller than 4,000/µl or larger than 10,000/µl), increase of serum CRP levels (defined as 1 mg/dl or higher), and other changes indicating inflammation)
2. Cholestasis
    a. Jaundice (defined as a total bilirubin of 2 mg/dl or higher)
    b. Laboratory data: abnormal liver function tests (increased serum alkaline phosphatase, gamma-glutamyltransferase (gamma-GT), aspartate transaminase (AST) and ALT levels (defined as more than 1.5 times the ULN))
3. Imaging
    a. Biliary dilatation
    b. Evidence of the etiology on imaging (stricture, stone, stent etc.) (26)

Obstructive choledocholithiasis: presence of gallstones, microlithiasis or biliary sludge in the CBD on imaging, requiring an ERCP, according to the treating physician.

For peer review only

References

1. Banks PA, Bollen TL, Dervenis C, Gooszen HG, Johnson CD, Sarr MG, et al. Classification of acute pancreatitis--2012: revision of the Atlanta classification and definitions by international consensus. Gut. 2013;62(1):102-11.

2. Working Group IAP/APA Acute Pancreatitis Guidelines. IAP/APA evidence-based guidelines for the management of acute pancreatitis. Pancreatology. 2013;13(4 Suppl 2):e1-15.

3. Stolle M, Sack PM, Thomasius R. Binge drinking in childhood and adolescence: epidemiology, consequences, and interventions. Dtsch Arztebl Int. 2009;106(19):323-8.

4. Sadr Azodi O, Orsini N, Andren-Sandberg A, Wolk A. Effect of type of alcoholic beverage in causing acute pancreatitis. Br J Surg. 2011;98(11):1609-16.

5. Midanik LT. Drunkenness, feeling the effects and 5+ measures. Addiction. 1999;94(6):887- 97.

6. Ammori BJ, Boreham B, Lewis P, Roberts SA. The Biochemical Detection of Biliary Etiology of Acute Pancreatitis on Admission: A Revisit in the Modern Era of Biliary Imaging. Pancreas. 2003;26:e32-5.

7. Schepers NJ, Bakker OJ, Besselink MG, Bollen TL, Dijkgraaf MG, van Eijck CH, et al. Early biliary decompression versus conservative treatment in acute biliary pancreatitis (APEC trial): study protocol for a randomized controlled trial. Trials. 2016;17:5.

8. Schneider A, Lohr JM, Singer MV. The M-ANNHEIM classification of chronic pancreatitis: introduction of a unifying classification system based on a review of previous classifications of the disease. J Gastroenterol. 2007;42(2):101-19.

9. Sarner M, Cotton PB. Classification of pancreatitis. Gut. 1984;25:756-9.

10. Ratjen F, Döring G. Cystic fibrosis. The Lancet. 2003;361(9358):681-9.

11. Spanier B, Bruno MJ, Dijkgraaf MG. Incidence and mortality of acute and chronic pancreatitis in the Netherlands: a nationwide record-linked cohort study for the years 1995-2005. World J Gastroenterol. 2013;19(20):3018-26.

12. CBS StatLine. Households; size, position in household, January 1st 1995-2013 StatLine: Central Bureau for Statistics 2015 [Available from: http://statline.cbs.nl/Statweb/publication/?VW=T&DM=SLNL&PA=37312&D1=a&D2=0,5,10,(l-2)-l&HD=180221-1109&HDR=G1&STB=T.

For peer review only

13. Whitcomb DC. Genetic aspects of pancreatitis. Annu Rev Med. 2010;61:413-24.

14. Whitcomb DC. Hereditary diseases of the pancreas. In: Yamada T AD, Kaplowitz N, Laine L, Owyang C, Powell DW, editors, editor. Textbook of Gastroenterology. 4th ed. Philadelphia: Lippincott Williams & Wilkins; 2003. p. 2147–65.

15. Shane E, Dinaz I. Hypercalcemia: Pathogenesis, clinical manifestations, differential diagnosis and management. In: Favus M, editor. Primer on the metabolic bone diseases and disorders of mineral metabolism. 6th ed. Philadelpiha: Kippincott, Williams, and Wilkins; 2006. p. 26-176.

16. Berglund L, Brunzell JD, Goldberg AC, Goldberg IJ, Sacks F, Murad MH, et al. Evaluation and treatment of hypertriglyceridemia: an Endocrine Society clinical practice guideline. J Clin Endocrinol Metab. 2012;97(9):2969-89.

17. van Brunschot S, van Grinsven J, van Santvoort HC, Bakker OJ, Besselink MG, Boermeester MA, et al. Endoscopic or surgical step-up approach for infected necrotising pancreatitis: a multicentre randomised trial. The Lancet. 2018;391(10115):51-8.

18. Nitsche C, Maertin S, Scheiber J, Ritter CA, Lerch MM, Mayerle J. Drug-induced pancreatitis. Curr Gastroenterol Rep. 2012;14(2):131-8.

19. Lankisch PG, Apte M, Banks PA. Acute pancreatitis. The Lancet. 2015;386(9988):85-96.

20. Jungst C, Kullak-Ublick GA, Jungst D. Gallstone disease: Microlithiasis and sludge. Best Pract Res Clin Gastroenterol. 2006;20(6):1053-62.

21. Murphy JB. The diagnosis of gall-stones. Am Med News. 1903;82:825.33.

22. Cotton P. Congenital anomaly of pancreas divisum as cause of obstructive pain and pancreatitis. Gut. 1980;21:105-14.

23. Cotton PB, Lehman G, Vennes J, Geenen JE, Russell RCG, Meyers WC, et al. Endoscopic sphincterotomy complications and their management: an attempt at consensus. Gastrointest Endosc. 1991;37:383-93.

24. Connor S. Defining post-operative pancreatitis as a new pancreatic specific complication following pancreatic resection. HPB (Oxford). 2016;18(8):642-51.

25. Booth F, Flint L. Pancreatoduodenal trauma. In: ed BJ, editor. Blunt multiple trauma. New York: Marcel Dekker; 1990. p. 497-509.

For peer review only

26. Miura F, Okamoto K, Takada T, Strasberg SM, Asbun HJ, Pitt HA, et al. Tokyo Guidelines 2018: initial management of acute biliary infection and flowchart for acute cholangitis. J Hepatobiliary Pancreat Sci. 2018;25(1):31-40.

27. Yokoe M, Takada T, Strasberg SM, Solomkin JS, Mayumi T, Gomi H, et al. TG13 diagnostic criteria and severity grading of acute cholecystitis (with videos). Journal of Hepato-Biliary-Pancreatic Sciences. 2013;20(1):35-46.

28. French E, Robb W. Biliary and renal colic. Br Med J. 1963:135-8.

For peer review only

Additional file 3: Short Form 36 Questionnaire

English version

1. In general, would you say your health is:
   - ☐ excellent
   - ☐ very good
   - ☐ good
   - ☐ fair
   - ☐ poor

2. **Compared to one ear ago**, how would you rate your health in general **now**?
   - ☐ much better now than one year ago
   - ☐ somewhat better now than one year ago
   - ☐ about the same
   - ☐ somewhat worse now than one year ago
   - ☐ much worse now than one year ago

For peer review only

3. The following items are about activities you might do during a typical day. Does **your health now limit you** in these activities? If so, how much?

	Yes, limited a lot	Yes, limited a little	No, not limited at all
a. **Vigorous activities**, such as running, lifting heavy objects, participating in strenuous sports	☐	☐	☐
b. **Moderate activities**, such as moving a table, pushing a vacuum cleaner, bowling, or playing golf	☐	☐	☐
c. Lifting or carrying groceries	☐	☐	☐
d. Climbing **several** flights of stairs	☐	☐	☐
e. Climbing **one** flight of stairs	☐	☐	☐
f. Bending, kneeling, or stooping	☐	☐	☐
g. Walking **more than a mile**	☐	☐	☐
h. Walking **several blocks**	☐	☐	☐
i. Walking **one block**	☐	☐	☐
j. Bathing or dressing yourself	☐	☐	☐

For peer review only

4. During the **past 4 weeks**, have you had any of the following problems with your work or other regular daily activities **as a result of your physical health**?

	Yes	No
a. Cut down the **amount of time** you spent on work or other activities	☐	☐
b. **Accomplished less** than you would like	☐	☐
c. Were limited in the **kind** of work or other activities	☐	☐
d. Had **difficulty** performing the work or other activities (for example, it took extra effort)	☐	☐

5. During the **past 4 weeks**, have you had any of the following problems with your work or other regular daily activities **as a result of any emotional problems** (such as feeling depressed or anxious)?

	Yes	No
a. Cut down the **amount of time** you spent on work or other activities	☐	☐
b. **Accomplished less** than you would like	☐	☐
c. Didn't do work or other activities as **carefully** as usual	☐	☐

For peer review only

6. During the **past 4 weeks**, to what extent has your physical health or emotional problems interfered with your normal social activities with family, friends, neighbors, or groups?

☐ not at all
☐ slightly
☐ moderately
☐ quite a bit
☐ extremely

7. How much **bodily** pain have you had during the **past 4 weeks**?

☐ none
☐ very mild
☐ mild
☐ moderate
☐ severe
☐ very severe

8. During the **past 4 weeks**, how much did **pain** interfere with your normal work (including both work outside the home and housework)?

☐ not at all
☐ a little bit
☐ moderately
☐ quite a bit
☐ extremely

For peer review only

9. These questions are about how you feel and how things have been with you **during the past 4 weeks**. For each question, please give the one answer that comes closest to the way you have been feeling.

How much of the time during the **past 4 weeks**...	**All of the time**	**Most of the time**	**A good bit of the time**	**Some of the time**	**A little of the time**	**None of the time**
a. Did you feel full of pep?	☐	☐	☐	☐	☐	☐
b. Have you been a very nervous person?	☐	☐	☐	☐	☐	☐
c. Have you felt so down in the dumps that nothing could cheer you up?	☐	☐	☐	☐	☐	☐
d. Have you felt calm and peaceful?	☐	☐	☐	☐	☐	☐
e. Did you have a lot of energy?	☐	☐	☐	☐	☐	☐
f. Have you felt downhearted and blue?	☐	☐	☐	☐	☐	☐
g. Did you feel worn out?	☐	☐	☐	☐	☐	☐
h. Have you been a happy person?	☐	☐	☐	☐	☐	☐
i. Did you feel tired?	☐	☐	☐	☐	☐	☐

For peer review only

10. During the **past 4 weeks**, how much of the time has **your physical health or emotional problems** interfered with your social activities (like visiting with friends, relatives, etc.)?

- ☐ all of the time
- ☐ most of the time
- ☐ some of the time
- ☐ a little of the time
- ☐ none of the time

11. How TRUE or FALSE is **each** of the following statements for you?

	Definitely true	Mostly true	Don't know	Mostly false	Definitely false
a. I seem to get sick a little easier than other people.	☐	☐	☐	☐	☐
b. I am as healthy as anybody I know.	☐	☐	☐	☐	☐
c. I expect my health to get worse.	☐	☐	☐	☐	☐
d. My health is excellent.	☐	☐	☐	☐	☐

Space for additional remarks with the questionnaire:

For peer review only

Dutch version

1. Wat vindt u, over het algemeen genomen, van uw gezondheid?
   - ☐ uitstekend
   - ☐ zeer goed
   - ☐ goed
   - ☐ matig
   - ☐ slecht

2. In vergelijking *met 1 jaar geleden*, hoe zou u *nu* uw gezondheid in het algemeen beoordelen?
   - ☐ veel beter dan een jaar geleden
   - ☐ iets beter dan een jaar geleden
   - ☐ ongeveer hetzelfde als een jaar geleden
   - ☐ iets slechter dan een jaar geleden
   - ☐ veel slechter dan een jaar geleden

For peer review only

3. De volgende vragen gaan over dagelijkse bezigheden. Wordt u door **uw gezondheid** *op dit moment* beperkt bij deze bezigheden? Zo ja, in welke mate?

	**Ja, ernstig**	**Ja, een beetje beperkt**	**Nee, helemaal niet beperkt**
a. *Forse inspanning* (zoals hardlopen, zware voorwerpen tillen, inspannend sporten)	☐	☐	☐
b. *Matige inspanning* (zoals het verplaatsen van een tafel, stofzuigen, fietsen)	☐	☐	☐
c. Tillen of boodschappen dragen	☐	☐	☐
d. *Een paar* trappen oplopen	☐	☐	☐
e. *Eén* trap oplopen	☐	☐	☐
f. Buigen, knielen of bukken	☐	☐	☐
g. *Meer dan een kilometer* lopen	☐	☐	☐
h. Een halve kilometer lopen	☐	☐	☐
i. *Honderd meter* lopen	☐	☐	☐
j. Uzelf wassen of aankleden	☐	☐	☐

For peer review only

4. Had u, ten gevolge van uw **lichamelijke gezondheid**, *de afgelopen 4 weken* één van de volgende problemen bij uw werk of andere bezigheden?

	Ja	Nee
a. U heeft *minder tijd* kunnen besteden aan werk of andere bezigheden.	☐	☐
b. U heeft *minder bereikt* dan u zou willen.	☐	☐
c. U was beperkt in het *soort* werk of soort bezigheden.	☐	☐
d. U had moeite met het werk of andere bezigheden (het kostte u bijvoorbeeld extra inspanning).	☐	☐

5. Had u, ten gevolge van een **emotioneel probleem** (bijvoorbeeld doordat u zich depressief of angstig voelde), *de afgelopen 4 weken* één van de volgende problemen bij uw werk of andere bezigheden?

	Ja	Nee
a. U heeft *minder tijd* kunnen besteden aan werk of andere bezigheden.	☐	☐
b. U heeft *minder bereikt* dan u zou willen.	☐	☐
c. U heeft het werk of andere bezigheden niet zo zorgvuldig gedaan als u gewend bent.	☐	☐

For peer review only

6. In hoeverre heeft uw **lichamelijke gezondheid** of hebben uw **emotionele problemen** u *de afgelopen 4 weken* belemmerd in uw normale sociale bezigheden met gezin, vrienden, buren of anderen?

☐ helemaal niet
☐ enigszins
☐ nogal
☐ veel
☐ heel erg veel

7. Hoeveel **pijn** had u *de afgelopen 4 weken?*

☐ geen
☐ heel licht
☐ licht
☐ nogal
☐ ernstig
☐ heel ernstig

8. In welke mate heeft **pijn** u *de afgelopen 4 weken* belemmerd bij uw normale werkzaamheden (zowel werk buitenshuis als huishoudelijk werk)?

☐ helemaal niet
☐ enigszins
☐ nogal
☐ veel
☐ heel erg veel

For peer review only

12. De volgende vragen gaan over hoe u zich *de afgelopen 4 weken* heeft **gevoeld**. Wilt u bij elke vraag het antwoord aankruisen dat het beste aansluit bij hoe u zich heeft gevoeld?

Hoe vaak gedurende *de afgelopen 4 weken:*	**Voortdurend**	**Meestal**	**Vaak**	**Soms**	**Zelden**	**Nooit**
a. voelde u zich levenslustig?	☐	☐	☐	☐	☐	☐
b. voelde u zich erg zenuwachtig?	☐	☐	☐	☐	☐	☐
c. zat u zo erg in de put dat niets u kon opvrolijken?	☐	☐	☐	☐	☐	☐
d. voelde u zich kalm en rustig?	☐	☐	☐	☐	☐	☐
e. voelde u zich erg energiek?	☐	☐	☐	☐	☐	☐
f. voelde u zich neerslachtig en somber?	☐	☐	☐	☐	☐	☐
g. voelde u zich uitgeblust?	☐	☐	☐	☐	☐	☐
h. voelde u zich gelukkig?	☐	☐	☐	☐	☐	☐
i. voelde u zich moe?	☐	☐	☐	☐	☐	☐

For peer review only

10. *Hoe vaak* hebben uw **lichamelijke gezondheid** of **emotionele problemen** gedurende *de afgelopen 4 weken* uw sociale activiteiten (zoals bezoek aan vrienden of naaste familieleden) belemmerd?

- ☐ voortdurend
- ☐ meestal
- ☐ soms
- ☐ zelden
- ☐ nooit

11. Wilt u het antwoord kiezen dat het beste weergeeft hoe juist of onjuist u elk van de volgende uitspraken voor uzelf vindt?

	**Volkomen juist**	**Grotendeels juist**	**Weet ik niet**	**Grotendeels onjuist**	**Volkomen onjuist**
a. Ik lijk gemakkelijker ziek te worden dan andere mensen.	☐	☐	☐	☐	☐
b. Ik ben net zo gezond als andere mensen die ik ken.	☐	☐	☐	☐	☐
c. Ik verwacht dat mijn gezondheid achteruit zal gaan.	☐	☐	☐	☐	☐
d. Mijn gezondheid is uitstekend.	☐	☐	☐	☐	☐

Ruimte voor aanvullende opmerkingen bij de vragenlijst:

For peer review only
